# Hypoxia differentially affects coronary vessel formation during heart development

**DOI:** 10.1093/cvr/cvag124

**Published:** 2026-06-08

**Authors:** Sophie Payne, Susann Bruche, Dorota Szumska, Lucija Fleisinger, Alice Neal, Mark D Preston, Sarah De Val

**Affiliations:** Department of Physiology, Anatomy and Genetics, Institute of Developmental & Regenerative Medicine (IDRM), University of Oxford, Oxford OX3 7TY, UK; Department of Physiology, Anatomy and Genetics, Institute of Developmental & Regenerative Medicine (IDRM), University of Oxford, Oxford OX3 7TY, UK; Department of Physiology, Anatomy and Genetics, Institute of Developmental & Regenerative Medicine (IDRM), University of Oxford, Oxford OX3 7TY, UK; Department of Physiology, Anatomy and Genetics, Institute of Developmental & Regenerative Medicine (IDRM), University of Oxford, Oxford OX3 7TY, UK; Department of Physiology, Anatomy and Genetics, Institute of Developmental & Regenerative Medicine (IDRM), University of Oxford, Oxford OX3 7TY, UK; Prismea Limited, 7 Bell Yard, London WC2A 2JR, UK; Department of Physiology, Anatomy and Genetics, Institute of Developmental & Regenerative Medicine (IDRM), University of Oxford, Oxford OX3 7TY, UK

**Keywords:** Hypoxia, Coronary vasculature, Heart development, VEGFA, Angiogenesis

## Abstract

**Aims:**

The coronary vessel system is a dense and diverse network of arteries, veins, and capillaries formed by endothelial cells from a variety of sources. While hypoxia is a known stimulus for angiogenic sprouting generally, the exact mechanisms by which hypoxia, and resultant increased VEGFA, influences vessel growth in the heart are not clearly delineated.

**Methods and results:**

We used a genetic model to mimic hypoxia through ectopic stabilization of myocardial HIFα. This enabled us to study the consequences of hypoxia without vascular depletion. Changes in coronary endothelial cells (ECs) in these hearts relative to littermate controls were assessed by single-cell RNA sequencing, and by examining the activity of enhancer:reporter transgenes active in different coronary vessel beds downstream of distinct vascular regulatory pathways. Analysis of hypoxia-mimic hearts found increased angiogenic gene expression alongside expanded activity of the VEGFA-MEF2-driven angiogenic regulatory pathway in a pattern that indicated increased endocardial-derived angiogenic sprouting. Conversely, regulatory pathways specifically active in the sinus venosus (SV)-derived plexus showed little variance, and sprouting from the SV was not expanded. Although hypoxia and increased VEGFA levels have been previously linked to increased arterial differentiation, we saw little change in initial arterial EC differentiation in the experimental hypoxia-mimic hearts. However, mature coronary arterial formation was delayed.

**Conclusion:**

These observations further emphasize a direct and specific link between the hypoxia pathway and endocardial coronary vessel sprouting and suggest a role of hypoxia/VEGFA in guiding coronary arterial coalescence.

## Graphical Abstract

**Graphical Abstract:**
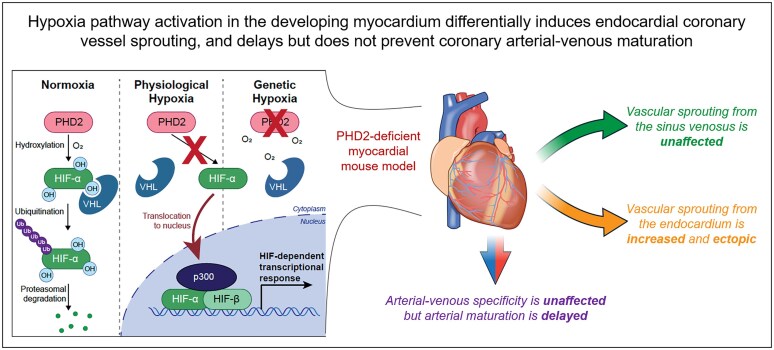

**Time of primary review: 34 days**

**See the editorial comment for this article ‘Different responses of coronary endothelial populations to hypoxia during heart development’, by A. Łoboda and J. Dulak, https://doi.org/10.1093/cvr/cvag144.**

## 1. Introduction

Endothelial cells (ECs) form the inner layer of all blood vessels and are the first component of the vascular system to form. Whilst the initial endothelial plexus in the early embryo is established by EC differentiation from mesodermal progenitors (vasculogenesis), most subsequent growth of new blood vessels occurs from existing ones, a process known as angiogenesis. During sprouting angiogenesis, the most intensely investigated form of angiogenesis, ECs take on a filopodia-rich migratory tip cell phenotype and invade the interstitial space, followed by highly proliferative ECs known as stalk cells.^1–3^ Sprouting angiogenesis can be induced by hypoxic conditions and is stimulated by growth factors such as VEGFA (via VEGFR2) and chemokines such as CXCL12 (via CXCR4).^1–3^

Although initially avascular, the high metabolic demands of the heart necessitate the formation of a coronary vasculature during embryonic development, a process that begins on embryonic day 11 (E11) in mice. Coronary vessels are formed by ECs from existing vascular structures and therefore grow via angiogenesis by definition. However, the ECs within the coronary vasculature come from multiple developmental origins, with the endocardium and sinus venosus (SV) representing the two main sources.^4–7^ The endocardium, an inner layer of specialized ECs within the heart, contributes to coronary vessels via endothelial sprouting outwards into the myocardium, a process which has been linked to hypoxia-induced myocardial VEGFA.^2,6^ Concurrently, venous ECs from the SV, a transient developmental structure at the venous pole of the heart, de-differentiate, sprout and migrate down the dorsal aspect of the heart to form a second coronary vascular plexus.^5,7,8^ However, although canonical Pdgfb-positive tip cells form during angiogenesis from both the endocardium and the subepicardial SV-origin plexus,^9^ SV-derived coronary sprouting is primarily linked to VEGFC and Elabela signalling rather than VEGFA.^5,8^ The developmental and functional heterogeneity of coronary ECs during development is also reflected by distinct expression profiles of some vascular genes (e.g. *Aplnr*/*Apj* in SV-derived vessels, *Fabp5* in endocardial-derived^10–12^), and by differential activity of vascular enhancers (e.g. enhancers independently activated by VEGFA-MEF2, SOXF/RBPJ and BMP-SMAD1/5 are expressed in different compartments of the coronary vasculature^13^). However, despite their independent origins and stimuli, endocardial-derived and SV-derived coronary networks share expression of some angiogenic-associated genes and ECs from both origins contribute to coronary artery and vein formation.^9,12,14^ Additionally, these two vascular networks merge at late foetal stages to ensure the entire heart is vascularized, and ECs from the two origins are genetically and functionally indistinguishable from each other in the adult heart.^15^

It has been hypothesized that endocardial-derived coronary vascularization is selectively physiologically responsive to microenvironmental cues including hypoxia, whilst SV-derived coronary vascularization is instead developmentally timed and genetically driven.^8^ This grew out of observations that endocardial-derived vessels compensate for the loss of SV-derived vasculature during embryonic development, a redundancy attributed to increased myocardial hypoxia and subsequent VEGFA expression.^8^ However, these experiments were unable to directly assess whether hypoxia had a similar ability to activate SV-derived coronaries, as these were depleted in order to drive hypoxia in the first place. Additionally, coronary vessel growth in the adult heart, closely associated with hypoxia, does not originate from endocardial ECs but instead predominantly involves angiogenesis from established coronary vessels within the myocardial wall.^16^ It is therefore unclear whether this occurs via the same pathways involved in embryonic coronary formation. This is particularly important because the angiogenic response to myocardial infarction and ischaemic injuries in the adult mammalian heart is limited and insufficient. Efforts to improve new vessel growth therapeutically have focused on reactivating canonical angiogenic pathways such as VEGFA-VEGFR2 but have shown little success,^17^ with the responsiveness of coronary ECs to pro-angiogenic signals appearing reduced compared to ECs in other tissues.^18^

Here, we investigate the consequences of a hypoxic myocardium on the transcriptome of coronary ECs in the developing heart, and on different aspects of vascular growth in the heart as it becomes vascularized. This analysis uses myocardial deletion of *Phd2* to stabilize HIF and therefore mimic hypoxia without directly ablating any vascular beds. It takes advantage of the spatially distinct nature of coronary vascular development, allowing us to determine the effects of myocardial hypoxia on sprouting angiogenesis from different sources and driven by different pathways, and on arteriovenous differentiation and maturation.

## 2. Methods

### 2.1 Animals

All animal procedures were approved by a local ethical review committee at Oxford University, licensed by the UK Home Office, and followed ARRIVE guidelines. The published mouse alleles used were: *Phd2^flox/flox^*^19^; *Nkx2.5-Cre*^20^; *Rosa26-tdTomato*^21^*; ROSA26-LacZ*^22^; *HLX-3:LacZ,*^23^  *NOTCH1 + 16:LacZ,*^24^  *Dll4-12*:*LacZ,*^25^ and *Ephb4-2*:*LacZ.*^26^ All mice were back-crossed at least five generations onto a pure C57BL/6J background. Euthanasia was performed by cervical dislocation, and no experiments required anaesthetic agents.

For embryo collection, matings were set up and female mice checked for vaginal plugs every morning. The date of observation of vaginal plug was considered embryonic day (E)0.5. Embryos were harvested into phosphate-buffered saline (PBS) on ice, embryonic hearts then dissected and processed for X-gal staining, cryo-embedding in OCT Embedding Medium (Thermo Scientific), or paraffin embedding. Analysis of enhancer:reporter activity was limited to litters including both control and experimental embryos, as the coronary vasculature grows quickly over a short period so small differences in developmental timepoint between different litters can impact analysis.

Genotyping for *Phd2^flox^*, *Cre,* and *LacZ* was performed by PCR. Genomic DNA was extracted from ear biopsy (pups) or tail tip/yolk sac (embryos) samples by incubation for 1 h at 98°C in 100 µL 25 mM NaOH/0.2 mM EDTA, followed by addition of 100 µL 40 mM Tris-HCl (pH = 5.5). 1.5–2 µL of this digestion mix was used directly for the PCR reaction.

### 2.2 Single-cell RNA sequencing

*Phd2^fl/fl^;*Rosa26-*tdTomato^hom^* adult female mice were mated with *Phd2^fl/fl^;Nkx2.5-Cre^het^* males and plug checked each morning. Four *Phd2^fl/fl^* and four *Phd2^fl/fl^;Nkx2.5-Cre* embryos were harvested at E13.5 from a single litter, and hearts dissected on ice with outflow tracts and atria removed. Fluorescent microscopy was used to distinguish between control *Phd2^fl/fl^*;*Rosa26*-*tdTomato^het^* and *Phd2^fl/fl^;Nkx2.5-Cre*;*Rosa26*-*tdTomato^het^* hearts, with all genotypes later confirmed by PCR on tail tips. Each heart was processed as an individual sample for the scRNA-seq experiment, and all samples were processed together to minimize batch effects in the bioinformatic analysis.

For enzymatic and mechanical dissociation of the hearts, the Neonatal Heart Dissociation Kit (Miltenyi Biotec, 130-098-373) was used according to manufacturer’s instructions in combination with the gentleMACS™ Octo Dissociator with Heaters (Miltenyi Biotec), including the red blood cell lysis steps. The resulting cell pellets were then resuspended in 100 µL of 3% FBS in PBS containing 1:100 anti-mouse-CD31 conjugated to FITC (BioLegend, 102406) and incubated on ice for 25 min. 100 µL of 1 µg/mL DAPI solution was then added to each sample and incubated on ice for a further 5 min before cell suspensions were centrifuged at 600 × g for 5 min at 10°C. Cell pellets were then resuspended in 500 µL of 2% FBS in PBS, transferred to FACS tubes and kept on ice in the dark. Single-stained and unstained cells were also prepared as controls for setting up the FACS machine.

Cells were sorted using a Sony MA900 FACS Sorter, with gating set up first using an FSC-A vs. BSC-A plot to exclude cell debris, then an FSC-A vs. FSC-H plot for the exclusion of doublet cells. Cell viability was determined using DAPI staining, with low DAPI cells designated as ‘Live’ cells, and unstained/single-stained samples used to determine a suitable threshold for CD31-FITC staining. CD31-positive ECs from each sample were sorted into 100 µL 3% FBS in PBS. The FACS cell counts ranged from 5919 to 10580 per heart, and when all samples had been run on the Sorter the collected cells were centrifuged at 600 × g for 5 min at 10°C, then the supernatant was aspirated, and the cell pellets resuspended in 30 µL 0.04% BSA in PBS. Samples were then immediately processed for sequencing using the Chromium Next GEM Single Cell 3’ GEM, Library & Gel Bead Kit v3.1 from 10X Genomics according to manufacturer’s instructions. Each sample was processed in a separate GEM well, and then samples were pooled to a single flow cell and run on a NextSeq2000 sequencer. The raw read data was downloaded from Illumina BaseSpace for quality control and bioinformatic analysis.

### 2.3 Bioinformatic analysis

We followed the standard Cell Ranger pipelines to demultiplex the raw base call (BCL) files, align reads to the mouse mm10 reference genome, generate feature-barcode matrices, and aggregate the samples.^27^ The resulting sequencing and alignment statistics were used as quality controls (see Supplementary material online, *Figure S3A*, *B*). The dataset was used to create a SeuratObject using Seurat version 4.3 in R for further processing and analysis: following normalization across samples, cells were excluded if they had fewer than 200 or more than 7000 genes, contained more than 10% mitochondrial RNA, or were assessed as doublets using DoubletFinder.^28^ Datasets were projected into two-dimensional space using the uniform manifold approximation and projection (UMAP) algorithm, followed by unsupervised graph-based clustering.

Cluster annotation was performed manually based on the most upregulated genes per cluster and previously reported marker genes. Cell cycle status in cells from control and hypoxia-mimic hearts was assigned using the Seurat CellCycleScoring() function, and differentially expressed genes were assigned using the FindMarker() function with default Wilcoxon Rank Sum test. The gprofiler2 package was used for pathway analysis on genes upregulated (p_val_adj < 0.05 and avg_log2FC > 0.2) in all cells in hypoxic-like conditions.

For analysis on coronary ECs only, clusters 9 and 15 were selected as a subset before repeating UMAP projection, graph-based clustering, and annotation. For lists of top 10 up/downregulated genes per cluster ranked by *P* value/log2FC, only genes with a value of >0.1 in both pct.1 and pct.2 were included.

Raw and processed data are deposited in GEO, accession number GSE292395, https://www.ncbi.nlm.nih.gov/geo.

### 2.4 Wholemount immunostaining

After dissection in cold PBS, embryonic hearts were fixed for 30–60 min in 4% paraformaldehyde (PFA) in PBS at 4°C, before dehydration through a methanol/PBS series to 100% methanol and storage at −20°C. For immunostaining, hearts were rehydrated back through the methanol series to PBS, permeabilized for 1 h in 1% Triton-X100 in PBS at room temperature, and then incubated with primary antibodies diluted in 0.5% Triton-X100 in PBS at 4° for 1–4 days. Primary antibodies used were Armenian hamster anti-CD31 (Abcam, ab119341, diluted 1:200), rat anti-Endomucin (Santa Cruz, sc-65495, diluted 1:50), rabbit anti-NR2F2 (Abcam, ab211777, diluted 1:200), rabbit anti-smooth muscle Myosin heavy chain 11 (Abcam, ab125884, diluted 1:400), and goat anti-SOX17 (R&D, AF1924, diluted 1:500). After primary antibody incubation, hearts were washed 5 × 30 min in PBS, followed by overnight incubation at 4°C with Alexa Fluor^®^-conjugated secondary antibodies diluted 1:500 in 0.2% TritonX-100 in PBS. Hearts were finally washed for a further 5 × 30 min in PBS, fixed in 4% PFA in PBS for 30 min, rinsed twice in PBS and then mounted onto concave cavity microscope slides with Fluoromount™ aqueous mounting medium (Sigma-Aldrich). Confocal stacks were obtained using a Zeiss 710 MP confocal microscope and processed using Zen and ImageJ software.

### 2.5 Whole-mount X-gal staining

β-galactosidase expression from the *LacZ* gene was detected by X-gal staining. Following dissection of embryonic hearts in cold PBS, samples were fixed in 2% PFA/0.2% glutaraldehyde in PBS at 4°C for 30–60 min depending on embryonic stage. They were then washed twice for 20–30 min in Rinse solution (2 mM MgCl_2_, 0.2% Nonidet P40, 0.1% sodium deoxycholate in PBS) before incubation in Staining solution (Rinse solution containing 1 mg/mL 5-bromo-4-chloro-3-indolyl β-d-galactopyranoside (X-gal), 5 mM K_4_Fe(CN)_6_ and 5 mM K_3_Fe(CN)_6_). For visualizing the HLX-3:*LacZ* and Dll4-12:*LacZ* transgenes, hearts were stained at room temperature for 3 h, and for visualizing the NOTCH1 + 16:*LacZ*, EphB4-2:*LacZ* and Rosa26-*LacZ* Cre reporter samples were incubated in the Stain solution overnight. Imaging of whole hearts was performed using a stereo microscope (Leica M165C) equipped with a ProgRes CF Scan camera and ProgRes CapturePro software (Jenoptik). Only *Phd2^fl/fl^;Nkx2.5-Cre* hearts carrying one of the enhancer:*LacZ* transgenes with at least one *LacZ*-positive *Phd2^fl/fl^* control littermate were included in analysis, due to small changes in embryonic stage reached between litters and the rapid development the coronary vasculature.

Following X-gal staining, some hearts and embryos were dehydrated through an ethanol series, cleared using Histo-Clear (National Diagnostics) and paraffin-embedded for sectioning. 10 μm sections were de-waxed, counter-stained using Nuclear Fast Red (Electron Microscopy Services) and imaged using a NanoZoomer S210 slide scanner with NDP.view2 viewing software (Hamamatsu).

### 2.6 Immunostaining on cryosections

Dissected embryonic hearts were fixed for 45 min in 4% PFA in PBS at 4°C, then incubated overnight in 30% sucrose in PBS at 4°C. Following washes in a 50/50 mix of 30% sucrose/OCT Embedding Medium (Thermo Scientific) and then OCT, hearts were mounted in OCT over dry ice and stored at −80°C. Transverse cryosections were cut at a thickness of 10–15 µm and stored at −80°C until ready to use.

Cryosections were thawed at room temperature, before removal of the OCT by immersion in PBS, permeabilization in 0.5% Triton-X100 in PBS for 15 min, and further PBS washes. Sections were then incubated for 1 h in Blocking solution (4% FBS; 10% donkey serum; 0.2% Triton X-100 in PBS) at room temperature, before overnight incubation at 4°C in primary antibodies diluted in Blocking solution. Following washes in PBS then 0.1% Triton-X100 in PBS, sections were incubated in Alexa Fluor^®^-conjugated secondary antibodies diluted 1:500 in Blocking solution for 1 h at room temperature, before further PBS washes, nuclear counter-staining using DAPI, and mounting in Fluoromount™ aqueous mounting medium (Sigma-Aldrich). Immunostained sections were imaged using a Zeiss 710 MP confocal microscope and processed using Zen and ImageJ software.

Primary antibodies used were: rat anti-Endomucin (Santa Cruz, sc-65495, diluted 1:50), rabbit anti-RFP (Rockland, 600-401-379, diluted 1:1000), rabbit anti-NR2F2 (Abcam, ab211777, diluted 1:200), goat anti-SOX17 (R&D, AF1924, diluted 1:500), rabbit anti-Cx40 (Alpha Diagnostic International, CX40-A, 1:200), and rabbit anti-GLUT1 (Abcam, ab115730, diluted 1:100); as well as Isolectin GS-IB4 From Griffonia simplicifolia, Alexa Fluor™ 488 Conjugate (Invitrogen, I21411).

### 2.7 EdU incorporation assay

To assess endothelial cell proliferation, we used the Click-iT™ Plus EdU Cell Proliferation Kit for Imaging (Alexa Fluor™ 647 dye) from Invitrogen, combined with immunostaining for the endothelial-specific nuclear ERG protein. Pregnant dams were administered with 1.25 mg EdU dissolved in 150 µL sterile PBS via intraperitoneal injection, for an approximate dose of 50 mg/kg, when embryos were E11.5. Embryonic hearts were harvested 48 h later at E13.5, and torsos fixed in 4% PFA before paraffin embedding and sectioning at 8 µm thickness. The fluorescent EdU assay was performed as per the kit instructions, with an additional antigen retrieval step required for the ERG co-staining. Briefly, paraffin sections were de-paraffinized in Histo-Clear (National Diagnostics), re-hydrated through an ethanol series to PBS, and then antigen retrieval performed using Antigen Unmasking Citrate Buffer (Vector Labs) in a pressure cooker for 2 min at full temperature. Slides were rinsed in tap water, washed in 3% BSA in PBS, and then incubated for 30 min in the Click-iT^®^ Plus reaction cocktail protected from light. They were rinsed again in 3% BSA, blocked for 1 h in Blocking solution (1% BSA/2% FBS in PBS) and then incubated at 4°C overnight in rabbit anti-ERG antibody (Abcam, AB92513) diluted 1:100 in Blocking solution. Following PBS washes, slides were incubated at room temperature for 1 h in anti-rabbit-546 secondary antibody diluted 1:500 in Blocking solution, washed in 3% BSA, and counterstained with Hoechst™ 33 342 dye for 30 min at room temperature. After final PBS washes, sections were mounted in Fluoromount™ and imaged using a Zeiss 710 MP confocal microscope. Quantification of the resulting images is described below.

### 2.8 X-gal staining on cryosections

X-gal staining was performed directly on E15.5 cryosections, which were prepared as for immunostaining. Following thawing and PBS washes, sections were incubated in Fix solution (4% PFA, 2 mM MgCl_2_, 5 mM EGTA in PBS) for 10 min at room temperature, before further washes in PBS. They were then incubated in Cryosection Staining solution (2 mM MgCl_2_, 0.02% Nonidet P40, 0.01% sodium deoxycholate, 5 mM K_4_Fe(CN)_6_, 5 mM K_3_Fe(CN)_6_, and 1 mg/mL X-gal in PBS) in a humidified chamber overnight at room temperature. Once the desired staining intensity was reached, sections were washed in PBS, fixed again in 4% PFA for 15 min, washed in PBS, and counter-stained with Nuclear Fast Red. After dehydration and mounting, slides were imaged using a NanoZoomer S210 slide scanner with NDP.view2 viewing software (Hamamatsu).

### 2.9 *In situ* hybridization

*In situ* hybridization for detection of mRNAs was performed on paraffin sections using RNAScope^®29^. *Phd2^fl/fl^* and *Phd2^fl/fl^;Nkx2.5-Cre* littermate embryos at E13.5 and E15.5 were dissected in cold PBS and the chest region fixed overnight in 4% PFA in PBS at 4°C. They were then dehydrated through an ethanol series, cleared in Histo-Clear (National Diagnostics) and paraffin-embedded. Sections were cut at 6 µm thickness, air dried overnight and stored at room temperature. *Phd2^fl/fl^* and *Phd2^fl/fl^;Nkx2.5-Cre* littermate hearts were processed together for each experiment comparing gene expression between the two genotypes.

The RNAscope^®^ Multiplex Fluorescent Reagent Kit v2 (Bio-Techne Ltd) was used according to manufacturer’s instructions, with mild-standard conditions used for target retrieval (15 min in the Target Retrieval Reagent in a steamer) and protease digestion in the Protease Plus reagent for 30 min at room temperature. The probes used were Mm-Vegfa-C3 (436961-C3) and Mm-Vegfc (492701), with Opal 570 and Opal 690 Dyes (Akoya Biosciences) used to detect C1 and C3 channels.

Immediately following the RNAScope protocol, slides were processed for immunohistochemical detection of Endomucin. Briefly, slides were washed twice in PBS before incubation in a humidified chamber at room temperature for 1 h in Blocking solution (4% FBS, 10% donkey serum, 0.2% Triton X-100 in PBS). They were then incubated overnight at 4°C in rat anti-Endomucin antibody (Santa Cruz, sc-65495) diluted 1:50 in Blocking solution. After further washes and incubation for 1 h in Alexa Fluor^®^488-conjugated anti-rat secondary antibody (Life Technologies) diluted 1:500 in Blocking solution, slides were counterstained with DAPI and mounted using Fluoromount™ medium (Sigma-Aldrich). Confocal images were obtained using a Zeiss 710 MP confocal microscope and processed using Zen and ImageJ software.

### 2.10 Hybridization chain reaction (HCR™)

HCR™ was performed on wholemount embryonic hearts at E13.5 for detection of *Aplnr* mRNA, using the v3.0 reagents from Molecular Instruments. Briefly, E13.5 embryos were harvested on ice and tail tips collected for genotyping, followed by dissection of the hearts, which were fixed overnight in 4% PFA at 4°C before being dehydrated through a methanol/PBST series and stored at −20°C. Once genotyped, 3 *Phd2^fl/fl^* and 3 *Phd2^fl/fl^;Nkx2.5-Cre* littermate hearts were rehydrated on ice, rinsed in PBST, then immersed in 10 μg/mL proteinase K solution for 10 min at room temperature. They were rinsed again in PBST, post-fixed in 4% PFA for 20 min, and rinsed in PBST. For the detection stage, hearts were incubated in Probe Hybridization buffer (v3.0, Molecular Instruments) for 5 min at room temperature then in fresh Hybridization buffer for 30 min at 37°C. Probe solution was prepared by adding 4 pmol Aplnr-B3 probe to 500 µL Hybridization buffer, and hearts were incubated in this solution overnight at 37°C. Excess probes were then removed by washing in Probe Wash Buffer (Molecular Instruments) at 37°C, followed by washes in SSCT at room temperature. Hearts were incubated in Amplification buffer (Molecular Instruments), whilst Hairpin solution was prepared by adding snap-cooled h1 and h2 hairpins to Amplification buffer according to the manufacturer’s instructions. Hearts were incubated overnight in the Hairpin solution in the dark, before washing in SSCT solution and imaging using a Zeiss 710 MP confocal microscope.

### 2.11 Quantification of imaging data

For all quantification analysis, significance was tested using an unpaired t test, with *P* < 0.05 determined as significant, and all *N* numbers are included in figure legends.

Ventricular wall thickness of Nuclear Fast Red stained transverse sections through E15.5 *Phd2^fl/fl^* and *Phd2^fl/fl^;Nkx2.5-CRE* hearts was measured using tools in the NDP.view2 software (Hamamatsu), with measurement at the most lateral point of the right and left ventricles.

Progression of the SV-derived vascular plexus on either CD31-immunostained or X-gal-stained wholemount hearts at E12.5 and E13.5 was calculated manually in ImageJ, by measurement of the area from the SV to the furthest tips of the plexus as a percentage of the total area of the dorsal aspect of the ventricles.

Quantification of HLX-3 or NOTCH1 + 16 enhancer activity on the ventral aspect of E12.5 or E13.5 wholemount hearts was performed using a Macro in ImageJ to create a mask for blue X-gal staining through colour analysis (based on methodology in ^30^) and measure its area, which was then converted to a percentage of the total area of the ventricles.

The same colour analysis pipeline was used to measure the area of blue X-gal stain on E13.5 transverse sections through *Phd2^fl/fl^* and *Phd2^fl/fl^;Nkx2.5-CRE* hearts expressing the HLX-3:*LacZ* transgene, which again was calculated as a percentage of the total section area.

Quantification of RFP expression in EMCN-positive ECs was performed in ImageJ by creating a mask for EMCN staining and then calculating the proportion of this area also positive for RFP signal.

Quantification of Fluorescent RNAscope^®^ results was performed in QuPath based on technical notes from ACD/Bio-Techne (QuPath Analysis Guidelines, MK 51-154/Rev A/Date 12/21/2020). Briefly, the ventricles were selected as the region of interest (ROI), and cell segmentation based on DAPI nuclear staining performed using an intensity threshold of 10 and the following nucleus parameters: Background radius 10 µm, Median filter radius 2 µm, Sigma 2 µm, Minimum area 10 µm^2^, Maximum are 300 µm^2^. Vegfa or Vegfc probe detection was then performed using the subcellular detection module, with the following parameters that gave the best detection results: Detection threshold 50, Expected spot size 0.5 µm^2^, Minimum spot size 0.3 µm^2^, Maximum spot size 5 µm^2^. The percentage of cells containing at least one spot was calculated for each section and results compared across control and hypoxic-like genotypes. Of these positive cells, the mean of the estimated number of spots for each cell was also calculated for control and Cre-positive hearts.

Quantification of EdU assay data was performed similarly in QuPath, with cells in the ROI defined using the same nucleus parameters, followed by defining cells as endothelial (ERG+, based on mean 546 channel threshold) and/or proliferating (EdU+, based on mean 647 channel threshold). The percentage of ERG + endothelial cells that were also EdU + was then calculated for each section, and results compared across control and hypoxic-like genotypes.

## 3. Results

### 3.1 Cardiomyocyte-specific knockdown of *Phd2* mimics hypoxia in the developing heart

**Figure 1:**
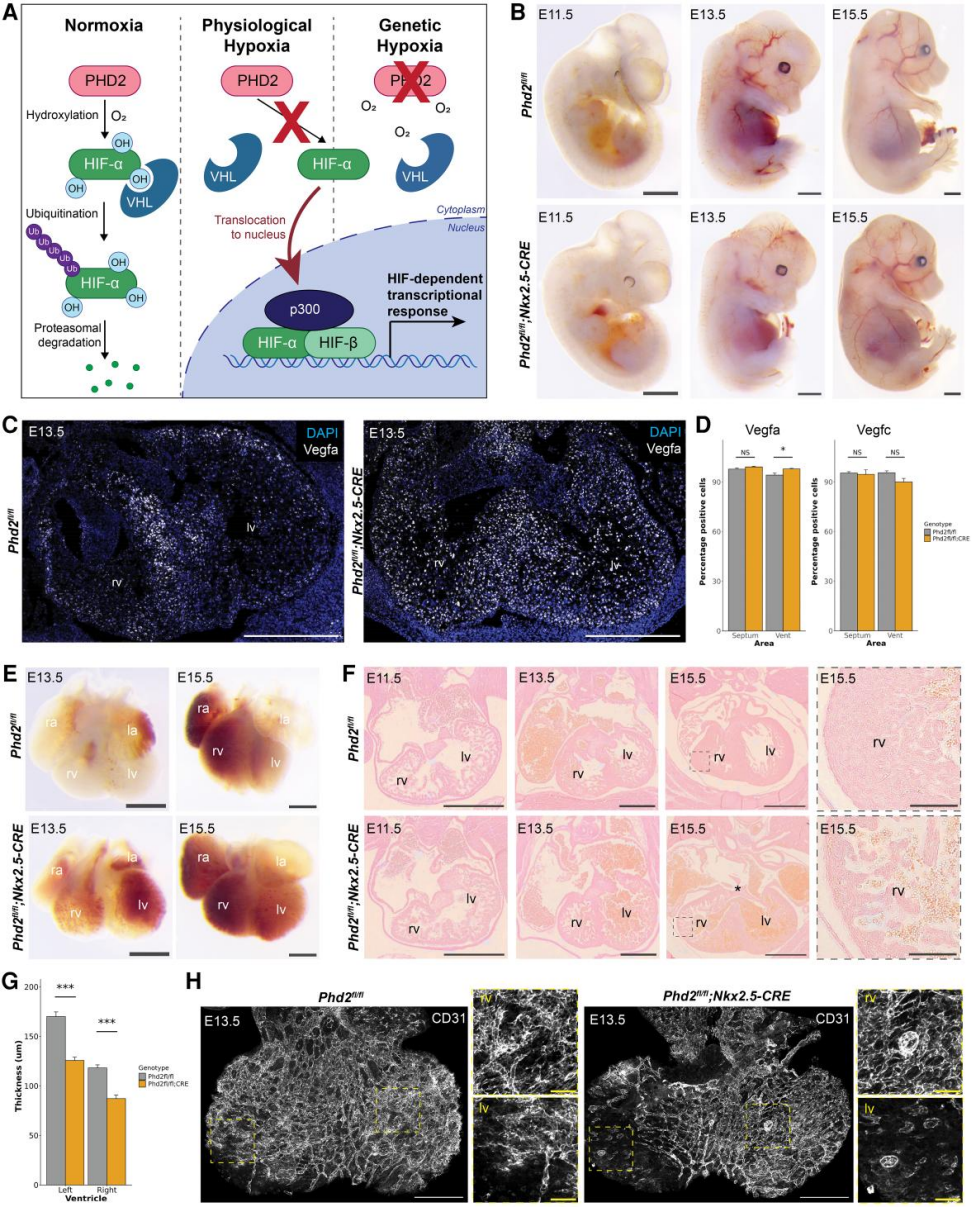
Myocardial-specific knockdown of *Phd2.* (*A*) Schematic summarizing use of genetic knockdown of *Phd2* to mimic hypoxia. (*B*) Representative littermate *Phd2^fl/fl^* and *Phd2^fl/fl^;Nkx2.5-Cre* embryos at embryonic day (*E*) 11.5, E13.5 and E15.5, scale bars = 1 mm. (*C*) *In situ* hybridization for *Vegfa* using RNAScope™ on transverse sections through littermate E13.5 *Phd2^fl/fl^* and *Phd2^fl/fl^;Nkx2.5-Cre* hearts, representative of two biological repeats. Scale bars = 0.5 mm. (*D*) Quantification of *Vegfa* and *Vegfc* transcripts in E13.5 hearts recorded as percentage of positive cells in either septum or free ventricular walls for each transcript. *P < 0.05, NS, not significant (unpaired *t* test), error bars show SEM, N = 2 biological replicates per genotype for each transcript. (*E*, *F*) Representative littermate *Phd2^fl/fl^* and *Phd2^fl/fl^;Nkx2.5-Cre* wholemount (*E*) or transversely sectioned hearts (*F*), scale bars = 0.5 mm, asterisk marks a ventricular septal defect, dashed grey boxes indicate magnified images (scale bars = 0.1 mm). (*G*) Quantification of myocardial wall thickness in left and right ventricles from *N* = 6 *Phd2^fl/fl^* and *Phd2^fl/fl^;Nkx2.5-Cre* hearts at E15.5, ***P < 0.0001 (unpaired *t* test), error bars show SEM. (*H*) Dorsal views of representative E13.5 *Phd2^fl/fl^* and *Phd2^fl/fl^;Nkx2.5-Cre* hearts immunostained for CD31, yellow boxes indicate magnified views. Additional images in Supplementary material online, *Figure S2*. Scale bars = 200 µm for main images and 50 µm for magnified views. rv, right ventricle; ra, right atrium; lv, left ventricle; la, left atrium.

Hypoxia-inducible factors (HIF) are transcription factors that act as the master regulators of the cellular response to hypoxia. Under normal oxygen conditions, prolyl hydroxylase domain 2 containing protein (PHD2, encoded by the *Phd2*/*Egln1* gene) targets HIF-1α and HIF-2α for degradation.^31^ Here, we crossed mice homozygous for the conditional *Phd2^flox^* allele^19^ with the constitutive *Nkx2.5-Cre* allele,^20^ which drives Cre expression in the cardiac crescent and cardiomyocytes from early in heart development prior to coronary vascular network formation. The activity of Nkx2.5-Cre was verified by crosses with ROSA26-*LacZ* and Rosa26-*tdTomato* alleles (see Supplementary material online, *Figure S1A*–*C*), which found widespread myocardial activity accompanied by little activity in cardiac ECs (e.g. 6% of EMCN-positive ECs in E13.5 hearts co-expressed expressing RFP, N = 4 biological replicates, 3–4 sections per heart). Therefore, our *Phd2^fl/fl^;Nkx2.5-Cre* mice lack PHD2 in the developing myocardium, which genetically mimics hypoxia by ectopically stabilizing HIFα proteins and inducing HIFα-dependent transcriptional programmes (*Figure 1A*).

*Phd2^fl/fl^;Nkx2.5-Cre* mice were viable and surviving adults showed no overt phenotype, although they were not born in expected Mendelian numbers (ratio of *Phd2^fl/fl^* to *Phd2^fl/fl^;Nkx2.5-Cre* mice surviving to P10-20 was 1.7:1, Chi squared equals 22.118 with 1 degree of freedom, two-tailed *P* value <0.0001, see Supplementary material online, *Figure S1E*). *Phd2^fl/fl^* females were crossed with *Phd2^fl/fl^;Nkx2.5-Cre* males, producing litters of control *Phd2^fl/fl^* and HIFα-stabilized, hypoxia-mimic *Phd2^fl/fl^;Nkx2.5-Cre* embryos (*Figure 1B*). Consistent with previous reports of hypoxia-induced increases in *Vegfa* transcription,^32,33^ loss of myocardial PHD2 resulted in increased *Vegfa* expression predominantly in the ventricular walls of *Phd2^fl/fl^;Nkx2.5-Cre* hearts (*Figure 1C, D*). In control (Cre-negative) hearts, *Vegfa* expression was highest in the interventricular septum (IVS) and lower in the ventricles (*Figure 1C*), consistent with previous reports.^6^ Conversely, *Vegfa* expression in *Phd2^fl/fl^;Nkx2.5-Cre* hearts was more uniform across the whole myocardium (*Figure 1C*). Quantification of these results showed a small but significant increase in the percentage of *Vegfa*-positive cells in the ventricular walls but not the IVS of *Phd2^fl/fl^;Nkx2.5-Cre* hearts (*Figure 1D*). Additionally, higher levels of *Vegfa* transcription were detected in *Phd2^fl/fl^;Nkx2.5-Cre* ventricles vs. controls (see Supplementary material online, *Figure S2A*), although this difference did not quite reach significance (*P* = 0.238, unpaired *t* test). *Vegfc* expression was not significantly altered between *Phd2^fl/fl^* and *Phd2^fl/fl^;Nkx2.5-Cre* heart sections (*Figure 1D*).

*Phd2^fl/fl^;Nkx2.5-Cre* embryonic hearts also exhibited some morphological defects. *Phd2^fl/fl^;Nkx2.5-Cre* hearts collected at E11.5 and E13.5 showed no overall gross phenotype, but by E15.5 the overall shape of the hearts was altered, with a more rounded apex and enlarged atria (*Figure 1E, F*). Additionally, the ventricular walls were significantly thinner than controls, indicating a failure of compaction of the myocardium (*Figure 1F, G*), and membranous ventricular septal defect were observed in two out of seven hearts (*Figure 1F*). Similar myocardial non-compaction defects have been previously reported in *Nkx2.5-Cre* and *Tnt-Cre-*driven deletion of *Vhl* (another critical component of hypoxia signalling, see *Figure 1A*), and were attributed to impaired cardiac maturation downstream of the disruption of a HIF1α-driven mid-gestational metabolic shift.^34^ These defects likely contributed to the reduced number of *Phd2^fl/fl^;Nkx2.5-Cre* mice surviving to postnatal stages. However, the myocardial non-compaction phenotype seen at E15.5 was able to resolve in some cases, as sections through *Phd2^fl/fl^;Nkx2.5-Cre* myocardium appear normal by P6 (see Supplementary material online, *Figure S1D*).

CD31 staining of the vasculature in embryonic hearts showed blood islands on the dorsal side of the heart exclusively in *Phd2^fl/fl^;Nkx2.5-Cre* hearts at E12.5 (1/2) and E13.5 (2/2), and slightly reduced expansion of the SV-derived vascular plexus on the left ventricle at E13.5 (*Figure 1H* and Supplementary material online, *Figure S2*, *B*, *C* and *E*). As expected, blood islands were also seen on the ventral aspect of all hearts at E13.5. Although there was a trend towards increased visible CD31-positive blood islands in *Phd2^fl/fl^;Nkx2.5-Cre* hearts (see Supplementary material online, *Figure S2*, *D* and *F*), this was non-significant.

### 3.2 Single cell RNA-sequencing analysis of ECs in *Phd2^fl/fl^;Nkx2.5-Cre* hearts

Next, we performed single cell RNA-sequencing on ECs isolated from *Phd2^fl/fl^* and hypoxia-mimic *Phd2^fl/fl^;Nkx2.5-Cre* hearts at E13.5. Briefly, littermate hearts were dissected and Cre positive hearts identified through recombination of the *Rosa26-tdTomato* reporter allele (*N* = 4 each of control and Cre-positive hearts). Each heart was processed as an individual sample for sequencing. Hearts were dissociated and CD31-positive ECs selected by FACS. Samples were then prepared for single-cell RNA sequencing using 10X Genomics Chromium Next GEM technology, and standard 10X Genomics bioinformatics analysis pipelines were used to process the resulting reads, see Supplementary material online, *Figure S3*, *A*, *B* for sequencing statistics and QC metrics.

**Figure 2:**
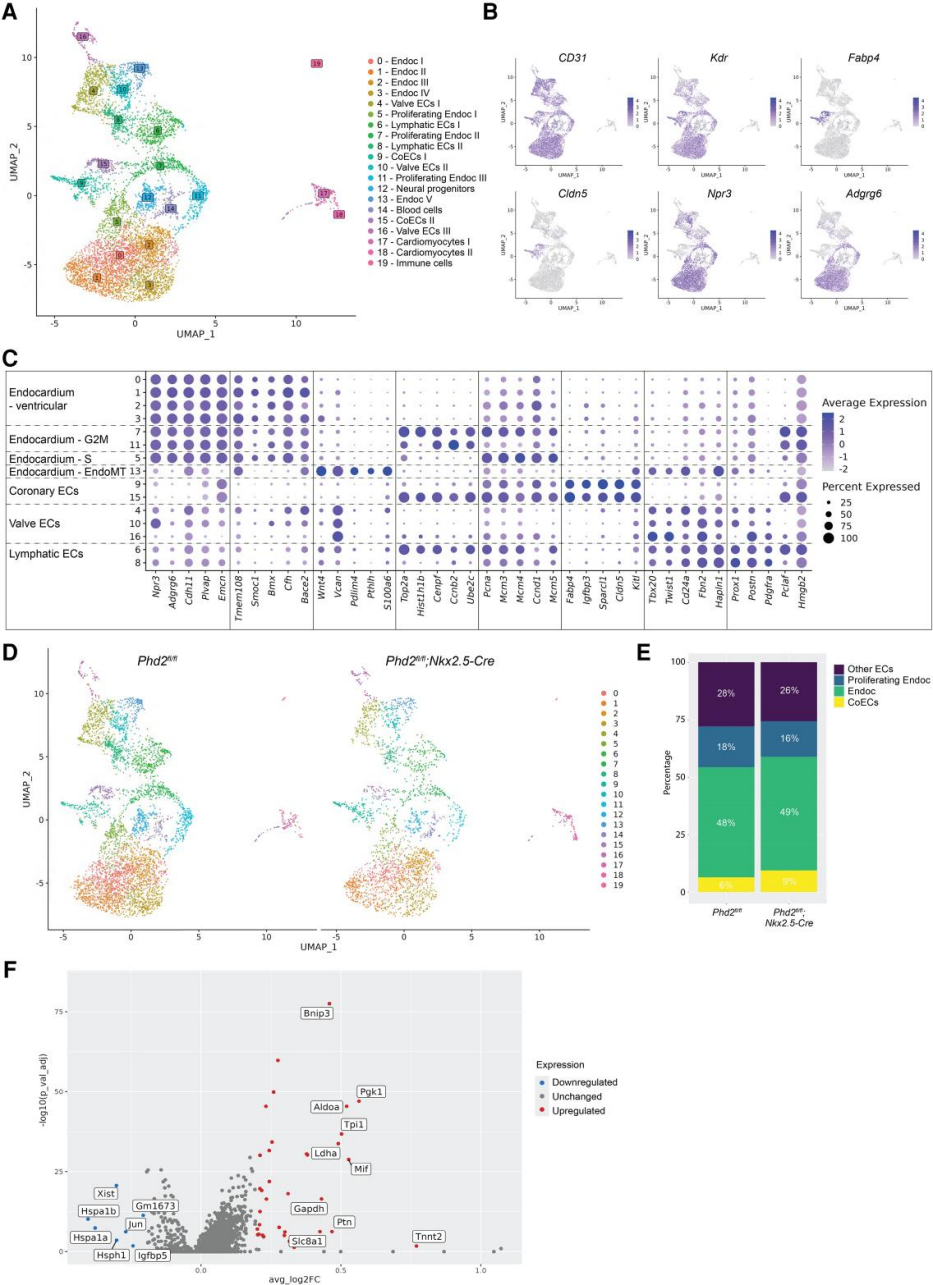
Single-cell sequencing analysis on ECs from *Phd2^fl/fl^* vs. *Phd2^fl/fl^;Nkx2.5-Cre* hearts at E13.5. (*A*) UMAP projection of the scRNAseq dataset showing 20 clusters. (*B*) Expression of key markers of endothelium (*CD31*, *Kdr*), coronary endothelium (*Fabp4*, *Cldn5*), and endocardium (*Npr3*, *Adgrg6*) visualized on UMAP projections. (*C*) Dot blot to show key genes defining each sub-group of EC clusters, plotted with relative expression in each cluster. (*D*) Side-by-side comparison of UMAP projections for the cells from control *Phd2^fl/fl^* and ‘hypoxic’ *Phd2^fl/fl^;Nkx2.5-Cre* hearts. (*E*) Bar chart to compare the percentage of cells in EC subtypes by genotype (coronary ECs (coECs) = clusters C9 and C15; Endocardium = C0, C1, C2, C3, and C13; Proliferating endocardium = C5, C11, and C13; Other ECs = C4, C6, C8, C10, and C16). n = 4336 *Phd2^fl/fl^* cells and 2400 *Phd2^fl/fl^;Nkx2.5-Cre* cells, percentage values rounded to nearest whole number. Chi squared test indicates significant difference in distribution of cell types between genotypes, *P* = 6.04 × 10^−6^. (*F*) Volcano plot to show up- and downregulated genes in *Phd2^fl/fl^;Nkx2.5-Cre* cells compared with *Phd2^fl/fl^* cells.

Cluster identification based on shared nearest neighbours resulted in 20 clusters (*Figure 2A*), 15 of which expressed common EC identity genes (*CD31, Kdr*) and were therefore assumed endothelial (*Figure 2B*). These EC clusters were split between endocardial (*Npr3* and *Adgrg6* positive) and coronary (*Fabp4* and *Cldn5* positive) ECs (*Figure 2B*): The four largest clusters (C0-3) all contained endocardial ECs, with high expression of markers for ventricular endocardium and transition to OFT, as defined by Cano *et al*.^9^ (*Figure 2C*). A fifth endocardial EC cluster (C13) was located closer to the three clusters of valve ECs (C4, C10 and C16), and contained markers for EndoMT endocardium. A further three clusters of endocardial ECs were identified as ‘proliferating endocardium’ due to the high expression of G2M (C7 and C11) or S phase (C5) cell cycle markers. In addition, we identified two *Fabp4+*; *Cldn5*+ clusters of coronary ECs (C9 and C15), the smaller of which (C15) also expressed G2M markers (*Figure 2C*).

Side by side comparison of the UMAP projections for *Phd2^fl/fl^* and *Phd2^fl/fl^;Nkx2.5-Cre* genotypes showed very similar distribution of cells across the clusters (*Figure 2D*), indicating that hypoxia-mimic myocardium neither results in the loss of an EC subtype or cell state, nor creates a new one. Dividing the EC clusters into broader groups representing endocardium, proliferating endocardium, coronary ECs and other ECs, we saw an increase of coronary ECs within the EC group in *Phd2^fl/fl^;Nkx2.5-Cre* hearts, rising approximately 50% from 6 to 9% (*Figure 2E*). Differential gene expression analysis across all ECs identified 45 differentially expressed genes (DEGs) in *Phd2^fl/fl^;Nkx2.5-Cre* cells compared with *Phd2^fl/fl^* cells (adjusted *P*-value after multiple testing correction <0.05 and log2 fold change >0.2, *Figure 2F* and Supplementary material online, *Table S1*). Of these, 37 genes were significantly upregulated in the hypoxia-mimic hearts, whilst 8 genes were significantly downregulated. Pathway analysis on the upregulated genes produced many terms associated with metabolic processes, particularly relating to glycolysis and carbon metabolism (see Supplementary material online, *Table S2*). This is consistent with previous reports that HIF1α expression in compact myocardium at E12.5 drives glycolytic metabolism, and of elevated oxidative phosphorylation activity in non-HIF1α-expressing trabeculae.^34^ In agreement, levels of the glucose transporter GLUT1, a direct HIF1 target gene, were increased in both the ventricles and inter-ventricular septum of *Phd2^fl/fl^;Nkx2.5-Cre* hearts at E13.5 compared to control *Phd2^fl/fl^* hearts (see Supplementary material online, *Figure S2G*).

We also ran a differential analysis on the cells from clusters C17 and C18, which were identified as cardiomyocytes. The total cell count was low (92 *Phd2^fl/fl^* cells and 122 *Phd2^fl/fl^;Nkx2.5-CRE* cells) so we increased the stringency of the parameters to adjusted *P*-value <0.01 and log2 fold change >1.2, producing 11 upregulated and 29 downregulated genes (see Supplementary material online, *Table S3*). *Phd2* (annotated here as *Egln1*) appears in the list of downregulated genes, validating our genetic model. Additionally, paracrine factors including *Cxcl12*, *Sema3a*, *Angpt1,* and *Slit2* were amongst the upregulated genes, indicating potential pathways that are affecting coronary EC growth.

### 3.3 Coronary ECs shift towards pro-angiogenic tip cell and non-cycling populations in *Phd2^fl/fl^;Nkx2.5-Cre* hearts

**Figure 3:**
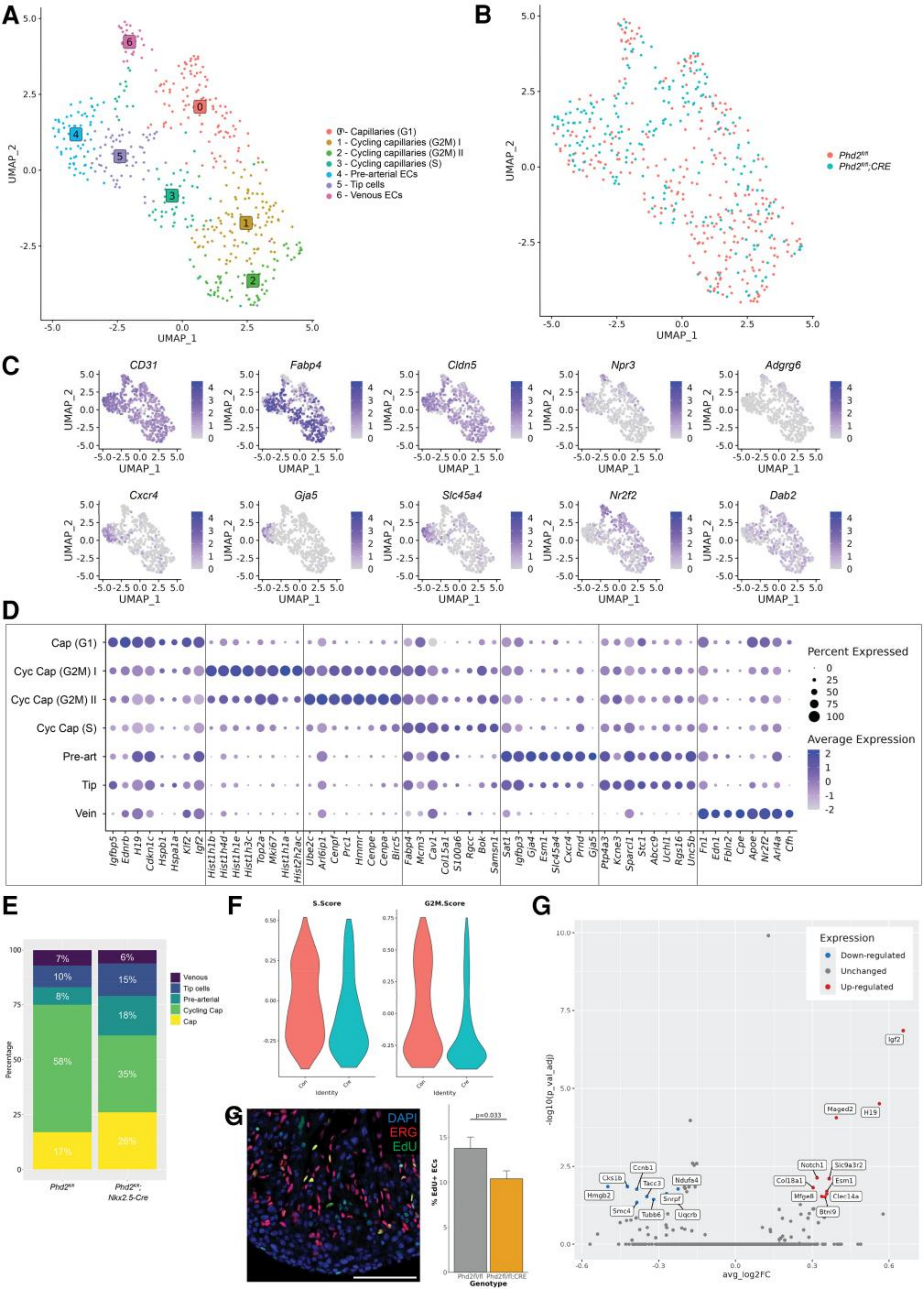
Single-cell sequencing analysis on coronary ECs only. (*A*) Re-running of UMAP projection and clustering of coronary EC cells resulted in 7 clusters. (*B*) Overlay comparison of UMAP projections for cells from *Phd2^fl/fl^* and *Phd2^fl/fl^;Nkx2.5-Cre* hearts. (*C*) Expression of key markers of endothelium (*CD31*), coronary endothelium (*Fabp4*, *Cldn5*), endocardium (*Npr3*, *Adgrg6*), arterial (*Cxcr4*, *Gja5*, *Slc45a4*), and venous (*Nr2f2*, *Dab2*) genes, visualized on UMAP projections. (*D*) Dot blot to show key genes defining each sub-group of coronary EC clusters, plotted with relative expression in each cluster. (*E*) Bar chart to compare the percentage of cells classified as G1 capillaries (Cap), G2/M/S cycling capillaries (Cycling cap), pre-arterial cells, tip cells and venous cells between *Phd2^fl/fl^* and *Phd2^fl/fl^;Nkx2.5-Cre* hearts. Percentage values rounded to nearest whole number. Chi squared test indicates significant difference in distribution of cell types between genotypes, *P* = 3.15 × 10^−6^. (*F*) Violin plots showing S and G2M cell cycle scores assigned to each cell from *Phd2^fl/fl^* (red) and *Phd2^fl/fl^;Nkx2.5-Cre* (blue) hearts. Wilcoxon test indicates significant differences in both S scores (*P* = 6.7 × 10^−5^) and G2M scores (*P* = 6.4 × 10^−9^). (*G*) Representative section through the ventricle of an E13.5 heart after 48 h exposure to EdU in utero, immunostained for EC-specific ERG and EdU. Yellow nuclei represent ERG + EdU + proliferating ECs, scale bar = 100 µm. Bar chart shows percentage of ERG + EdU + nuclei out of total number of ERG + nuclei for *Phd2^fl/fl^* and *Phd2^fl/fl^;Nkx2.5-Cre* E13.5 hearts. N = 4 biological replicates per genotype, 3–5 sections analysed per heart, *P* = 0.033 (unpaired t test), error bars represent SEM. (*H*) Volcano plot to show up- and downregulated genes in *Phd2^fl/fl^;Nkx2.5-Cre* cells compared with *Phd2^fl/fl^* cells.

To focus in on the coronary ECs, we selected the cells from clusters C9 and C15, and re-ran the UMAP generation and unsupervised clustering (see Supplementary material online, *Figure S3C* for QC metrics). There were 276 *Phd2^fl/fl^* and 225 *Phd2^fl/fl^;Nkx2.5-Cre* cells, which divided into 7 clusters (*Figure 3A*), and were positive for endothelial and coronary markers (*Figure 3C*). The four largest clusters were annotated as capillary coronary ECs, with cluster C0 showing G1 markers and clusters C1-3 showing cell cycle markers suggesting they are actively proliferating (*Figure 3D*). This reflects the relatively undifferentiated state of the coronary vasculature at E13.5, although smaller clusters were also identified containing *Gja5* and *Cxcr4*-positive pre-arterial cells, *Igfbp3* and *Kcne3*-positive tip cells, and *Nr2f2*-positive venous ECs, consistent with previously reported cell types at this stage (*Figure 3C, D*).

Overlaying the UMAP distributions of the coronary ECs from the *Phd2^fl/fl^* and *Phd2^fl/fl^;Nkx2.5-Cre* hearts again indicated that all clusters contained cells from both genotypes (*Figure 3B*). However, there was a drop in the proportion of cycling capillaries in *Phd2^fl/fl^;Nkx2.5-Cre* hearts (35%, compared with 58% of control), with an accompanying increase in non-cycling capillaries (26% compared with 17%) (*Figure 3E*). A greater proportion of coronary ECs were also assigned as tip cells and pre-arterial cells in the hypoxia-mimic hearts, whilst the proportion of venous cells remained similar. The differences in coronary ECs between *Phd2^fl/fl^* and *Phd2^fl/fl^;Nkx2.5-Cre* hearts was also reflected in cell cycle analysis of the cohorts: there is an overall decrease in the S and G2M scores of coronary ECs from the *Phd2^fl/fl^;Nkx2.5-Cre* hearts, consistent with the drop in cycling capillaries (*Figure 3F*). In order to validate this result, we performed an EdU assay alongside immunostaining for ERG to directly measure EC proliferation in *Phd2^fl/fl^* and *Phd2^fl/fl^;Nkx2.5-Cre* hearts. Following 48 h exposure to EdU, a significant reduction in the percentage of proliferating ECs was seen in the *Phd2^fl/fl^;Nkx2.5-Cre* hearts (10.4% vs. 13.8%, *P* = 0.0334, *Figure 3G*), in agreement with the scRNA-seq analysis.

Differential gene expression analysis of all coronary ECs identified 18 DEGs in the coronary ECs exposed to hypoxia-mimic myocardium that passed multiple testing corrections (*Table 1*, *Figure 3H*). The 10 up-regulated genes include genes linked with angiogenesis, EC migration and tube formation, such as *Esm1*, *Igf2*, *H19,* and *Clec14a.*^35^ Due to relatively small cell numbers, the same differential gene expression analysis on the individual EC clusters did not result in any significant DEGs after multiple testing correction. However, analysis of the most differentially altered genes (ranked by log2 fold change and *P* value) from each cluster suggested an increase in angiogenic-associated genes, particularly in the cycling capillaries and venous EC clusters, suggesting it is these coronary EC subtypes that are most likely to undergo transcriptional changes towards an angiogenic phenotype in response to environmental hypoxia or hypoxia mimicry (see Supplementary material online, *Table S4*).

**Table 1:** Differential gene expression analysis on all coronary ECs at E13.5

Gene	p_val	avg_log2FC	p_val_adj	Direction
***Igf2***	4.38E-12	0.655456846	1.41E-07	Upregulated
***H19***	9.71E-10	0.562078504	3.14E-05	Upregulated
***Maged2***	2.71E-09	0.393790579	8.75E-05	Upregulated
***Slc9a3r2***	2.48E-07	0.366018414	0.007991	Upregulated
***Esm1***	6.21E-07	0.356745856	0.020056	Upregulated
***Clec14a***	7.53E-07	0.356505389	0.024316	Upregulated
***Btnl9***	9.40E-07	0.349411029	0.030341	Upregulated
***Mfge8***	9.06E-07	0.337202444	0.029235	Upregulated
***Notch1***	2.32E-07	0.318960456	0.00749	Upregulated
***Col18a1***	4.73E-07	0.30355086	0.015269	Upregulated
***Snrpf***	7.49E-07	−0.269404535	0.024175	Downregulated
***Tubb6***	1.13E-06	−0.319949953	0.036531	Downregulated
***Tacc3***	9.20E-07	−0.346286681	0.029715	Downregulated
***Smc4***	1.42E-06	−0.38533039	0.045805	Downregulated
***Ccnb1***	5.31E-07	−0.385563554	0.017149	Downregulated
***Cks1b***	4.38E-07	−0.42225002	0.014144	Downregulated
***Hmgb2***	4.38E-07	−0.49867428	0.014147	Downregulated
***Rps2***	2.28E-40	−0.615811604	7.36E-36	Downregulated

Differential gene expression analysis results comparing all *Phd2^fl/fl^;Nkx2.5-Cre* with control *Phd2^fl/fl^* cells, showing 10 genes upregulated in *Phd2^fl/fl^;Nkx2.5-Cre* cells and 8 genes downregulated in *Phd2^fl/fl^;Nkx2.5-Cre* coronary ECs. Genes were included if adjusted *P*-value after multiple testing correction <0.05 and log2 fold change >0.2.

In conclusion, our scRNAseq analysis suggested an increase in angiogenic gene expression in coronary ECs in response to a hypoxic-like environment, with a shift towards the non-proliferative, migratory tip cell EC phenotype.

### 3.4 Hypoxia-mimic myocardium results in expanded and ectopic activation of the VEGFA-MEF2-dependent angiogenic pathway but does not affect SV-derived plexus formation

Our results so far indicate an increase in angiogenic ECs within the coronary vasculature in response to HIFα stabilization. These observations align with similar accelerated coronary vessel growth reported in hearts after myocardial deletion of *Vhl*, an alternative method of mimicking hypoxia.^36^ We next investigated whether the increased angiogenic ECs in *Phd2^fl/fl^;Nkx2.5-Cre* embryonic hearts were specific to endocardial-derived sprouting or instead represented an increase in angiogenesis from all coronary origins. Additionally, we sought to establish the vascular regulatory pathways targeted by these hypoxia-mimic conditions.

**Figure 4:**
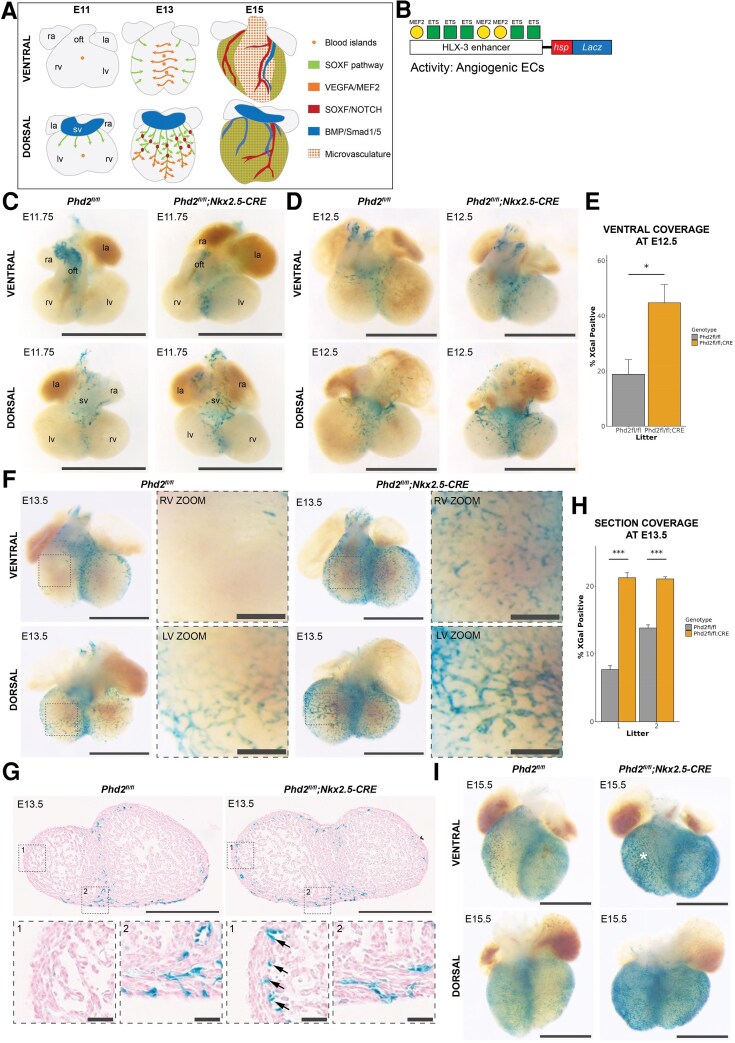
Expansion and ectopic activity of MEF2-dependent angiogenic pathway in hypoxic hearts. (*A*) Summary of regulatory pathways driving coronary vascular growth E11.5-E15.5.^13^ (*B*) Schematic of the HLX-3:*LacZ* transgene, with the human HLX-3 enhancer sequence cloned upstream of the hsp68 minimal promoter and *LacZ* reporter gene. Validated ETS and MEF2 TF binding sites within the enhancer are represented by coloured circles and squares.^23^ (*C*) Representative ventral and dorsal views of E11.75 littermate hearts with HLX-3:*LacZ* activity (blue X-gal stain) on *Phd2^fl/fl^* and *Phd2^fl/fl^;Nkx2.5-CRE* genetic backgrounds. N = 3 *Phd2^fl/fl^;Nkx2.5-CRE* hearts, all other hearts shown in Supplementary material online, *Figure S5A*. Scale bars = 1 mm. (*D*) Representative E12.5 littermate hearts with HLX-3:*LacZ* activity on *Phd2^fl/fl^* and *Phd2^fl/fl^;Nkx2.5-CRE* genetic backgrounds. N = 5 *Phd2^fl/fl^;Nkx2.5-CRE* hearts, all other hearts in Supplementary material online, *Figure S5B*. Scale bars = 1 mm. (*E*) Quantification of ventral area covered by HLX-3:*LacZ* activity on all hearts collected at E12.5, calculated as a percentage of the total ventral area. * = *P* < 0.05 (unpaired *t* test), error bars show SEM, N = 5 *Phd2^fl/fl^;Nkx2.5-CRE* hearts and N = 4 *Phd2^fl/fl^* hearts. (*F*) Representative E13.5 littermate *Phd2^fl/fl^* and *Phd2^fl/fl^;Nkx2.5-CRE* hearts with HLX-3:*LacZ* activity, with magnified views of the RV on ventral side, and LV on dorsal side, highlighting ectopic HLX-3:*LacZ* activity in *Phd2^fl/fl^;Nkx2.5-CRE* hearts. N = 7 *Phd2^fl/fl^;Nkx2.5-CRE* hearts, all other hearts shown in Supplementary material online, *Figure S6*. Scale bars = 1 mm in wholemount views and 0.2 mm in magnified images. (*G*) Transverse sections through E13.5 *Phd2^fl/fl^* and *Phd2^fl/fl^;Nkx2.5-CRE* hearts, showing blue HLX-3:*LacZ* activity. Zoom 1 illustrates ectopic endocardial activity in *Phd2^fl/fl^;Nkx2.5-CRE* hearts highlighted with black arrows, Zoom 2 shows similar X-gal-positive vessels migrating from the outer heart surface into the myocardium. Scale bars = 0.5 mm for main sections and 50 µm for magnified images. (*H*) Quantification of HLX-3:*LacZ* activity in two pairs of littermate E13.5 hearts, measuring the X-gal-positive area as a percentage of total area of transverse sections. *** = *P* < 0.0001 (unpaired *t* test), error bars show SEM. (*I*) Representative E15.5 littermate *Phd2^fl/fl^* and *Phd2^fl/fl^;Nkx2.5-CRE* hearts with HLX-3:*LacZ* activity, white asterisk highlights ectopic transgene activity on the ventral RV. N = 7 *Phd2^fl/fl^;Nkx2.5-CRE* hearts, all other hearts shown in Supplementary material online, *Figure S7*, including 3 *Phd2^fl/+^;Nkx2.5-CRE* hearts as CRE controls. Scale bars = 1 mm. rv, right ventricle; ra, right atrium; lv, left ventricle; la, left atrium; sv, sinus venosus; oft, outflow tract.

**Figure 5:**
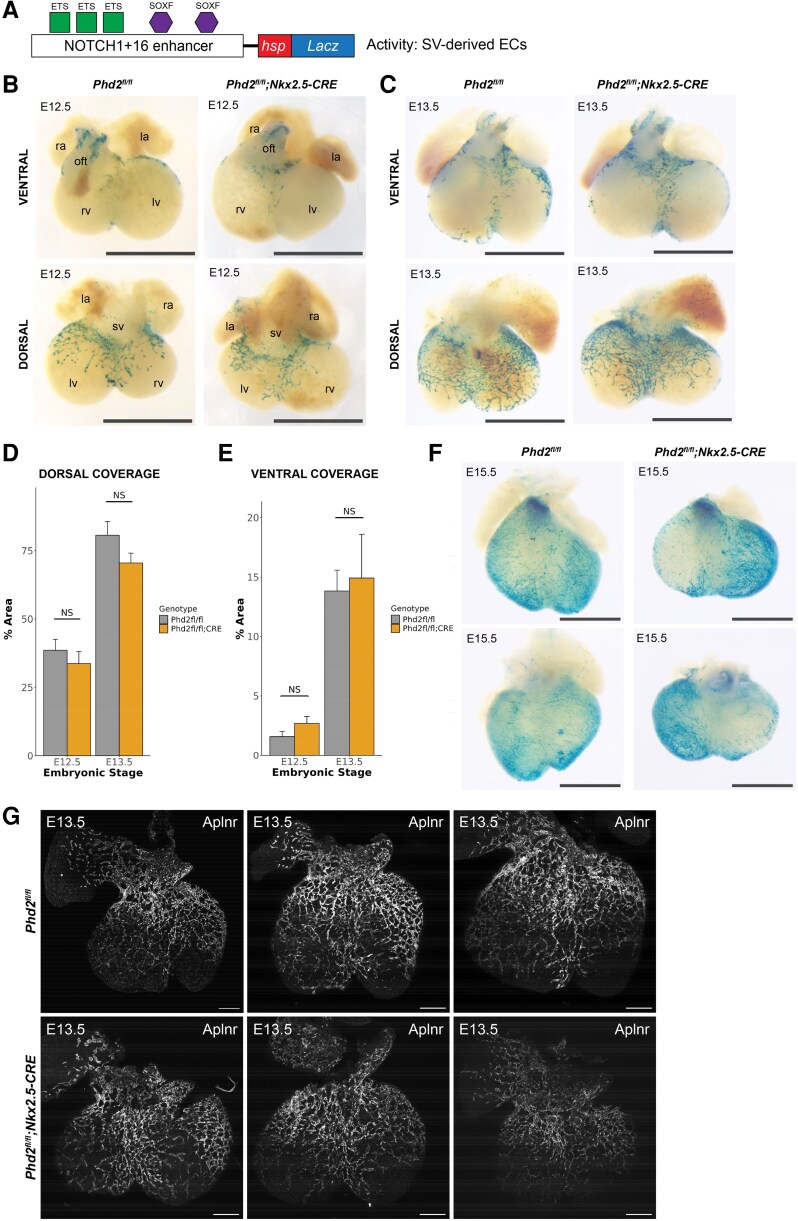
SV-derived vascular plexus formation is largely unaffected by hypoxic myocardium. (*A*) Schematic of the NOTCH1 + 16:*LacZ* transgene, with human NOTCH1­+16 enhancer sequence cloned upstream of the hsp68 minimal promoter and *LacZ* reporter gene. Validated ETS and SOXF TF binding sites within the enhancer are represented by coloured shapes.^24^ (*B)* Representative ventral and dorsal views of E12.5 littermate hearts with NOTCH1 + 16:*LacZ* activity (blue X-gal stain) on *Phd2^fl/fl^* and *Phd2^fl/fl^;Nkx2.5-CRE* genetic backgrounds. N = 7 *Phd2^fl/fl^;Nkx2.5-CRE* hearts, all other hearts shown in Supplementary material online, *Figure S8A*. Scale bars = 1 mm. ra = right atrium; la, left atrium; rv, right ventricle; lv, left ventricle; sv, sinus venosus; oft, outflow tract. (*C*) Representative E13.5 *Phd2^fl/fl^* and *Phd2^fl/fl^;Nkx2.5-CRE* hearts showing NOTCH1 + 16:*LacZ* activity. N = 5 *Phd2^fl/fl^;Nkx2.5-CRE* hearts, all other hearts shown in Supplementary material online, *Figure S8B*. Scale bars = 1 mm. (*D*, *E*) Quantification of the extension of the NOTCH1 + 16:*LacZ*-positive area as a percentage of the total dorsal (*D*) or ventral (*E*) area of *Phd2^fl/fl^* and *Phd2^fl/fl^;Nkx2.5-CRE* hearts at E12.5 and E13.5 hearts. NS, not significant (unpaired *t* test), error bars show SEM, N = 9 control and 7 *Phd2^fl/fl^;Nkx2.5-CRE* hearts at E12.5 and N = 3 control and 5 *Phd2^fl/fl^;Nkx2.5-CRE* hearts at E13.5. (*F*) E15.5 *Phd2^fl/fl^* and *Phd2^fl/fl^;Nkx2.5-CRE* hearts showing NOTCH1 + 16:*LacZ* activity. N = 6 *Phd2^fl/fl^;Nkx2.5-CRE* hearts, all other hearts shown in Supplementary material online, *Figure S9*. Scale bars = 1 mm. (*G*) Dorsal views of 3 *Phd2^fl/fl^* and 3 *Phd2^fl/fl^;Nkx2.5-CRE* hearts from the same litter, with *Aplnr* mRNA detected by Hybridization Chain Reaction (HCR™). Scale bars = 0.2 mm.

Our previous work on coronary vascular regulatory pathways identified two transcriptional pathways active during the sprouting of the coronary vasculature, differentially driven by MEF2 and SOXF factors.^13^ The MEF2 transcriptional pathway is downstream of VEGFA and is linked to expression of angiogenic genes such as *Hlx* and *Dll4* in the systemic vasculature, where it is specifically active in angiogenic ECs.^13^ In the developing embryonic heart, enhancers regulated by this VEGFA-MEF2 pathway are predominately active in ECs sprouting from the endocardium (*Figure 4A* and ^13^). Conversely, enhancers regulated by the SOXF pathway are predominantly active throughout the SV-derived endothelial plexus (*Figure 4A* and ^13^). Whilst these patterns of expression align with the concept of distinct VEGFA-driven endocardial sprouting (MEF2) and VEGFC/Elabella-driven SV-derived sprouting (SOXF), the activity patterns of enhancers downstream of these two pathways already hints at some level of greater complexity: VEGFA-MEF2-driven enhancers are also transiently active at the leading edge of the SV-derived plexus as it initially moves over the dorsal ventricle between E11.5-E13.5 (*Figure 4* and ^13^), suggesting that the classical angiogenic VEGFA-MEF2 pathways may be involved to some degree in both endocardial-derived and SV-derived sprouting. Conversely, whilst SOXF-driven enhancers are active throughout the SV-derived plexus as it sprouts down the dorsal side of the heart between E11.5-E13.5, they are also expressed in a subset of ECs emerging from the endocardium/septum region on the ventral side (*Figure 4A*, *Figure 5* and ^13^). This expression pattern closely mimics that of SOX17, which is widespread throughout the SV-derived vascular plexus but also in active ECs sprouting from the endocardium at these timepoints (see Supplementary material online, *Figure S4* and^8,12^), again suggesting that angiogenic sprouting from both endocardial and SV origins may incorporate multiple inputs via different regulatory pathways.

We first investigated the expression of the endocardial-sprout associated, VEGFA/MEF2-driven, angiogenic HLX-3 enhancer (*Figure 4B*) in *Phd2^fl/fl^;Nkx2.5-Cre* embryonic hearts relative to controls. Expression of the HLX-3:*LacZ* transgene in control *Phd2^fl/fl^* hearts was predominantly restricted to endocardial-derived vessels on the ventral aspect of the heart, in the septum, and in the ventricular free walls, with transient activity also seen at the angiogenic front of the developing SV-derived plexus. Conversely, *Phd2^fl/fl^;Nkx2.5-Cre* hearts showed expansion of HLX-3:*LacZ* activity throughout embryonic coronary vessel development (*Figure 4* and Supplementary material online, *Figures S5*–*S7*). At E11.75, a timepoint in which SV-derived and endocardial-derived EC sprouting first occurs, increased HLX-3:*LacZ* expression was detected in *Phd2^fl/fl^;Nkx2.5-Cre* hearts along the midline on both the ventral and dorsal sides of the heart in a pattern characteristic of endocardial vessel sprouting (*Figure 4C* and Supplementary material online, *Figure S5A*). This increased activity was more notable by E12.5, with X-gal expressing endocardial-associated sprouts on the ventral aspect significantly more widespread in *Phd2^fl/fl^;Nkx2.5-Cre* hearts compared to controls (*Figure 4D, E* and Supplementary material online, *Figure S5B*). By E13.5 more intense HLX-3:*LacZ* activity was also notable within the SV-derived plexus in the *Phd2^fl/fl^;Nkx2.5-Cre* hearts (*Figure 4F* and Supplementary material online, *Figure S6*). Enhancer activity was also seen in regions not usually populated by either endocardial-derived or SV-derived plexuses, particularly on the ventral aspect and the dorsal apex regions. Transverse sections through these hearts demonstrate an increase in HLX-3:*LacZ* activity in the endocardium and in ECs sprouting out of the endocardium (*Figure 4G*). Further, a greater proportion of the sections were populated by HLX-3:*LacZ-*positive vessels, indicating angiogenic expansion of the vascular network (*Figure 4G, H*). This correlates with the increased percentage of ‘tip cell’ ECs found in the scRNA-seq in *Phd2^fl/fl^;Nkx2.5-Cre* hearts relative to controls, and may be a consequence of ectopic activation of endocardial sprouting distal from the normal septum region in these hypoxia-mimic hearts.

*Phd2^fl/fl^;Nkx2.5-Cre* hearts continued to show increased HLX-3:*LacZ* activity at E15.5 across the entire surface of the ventricles, indicating sustained VEGFA-MEF2 pathway signalling and increased sprouting angiogenesis in the hypoxia-mimic heart compared with controls (*Figure 4I* and Supplementary material online, *Figure S7*). This was not detected in *Phd2^fl/+^;Nkx2.5-Cre* hearts, suggesting that Cre toxicity is not involved (see Supplementary material online, *Figure S7A*, litter 5). The expanded expression of HLX-3:*LacZ* in *Phd2^fl/fl^;Nkx2.5-Cre* hearts included a greater number of HLX-3:*LacZ*-expressing ECs in the septum region and an increase in HLX-3:*LacZ*-expressing ECs with blood island morphology on the ventral surface, both traditionally associated with endocardial-derived sprouting (*Figure 4I* and Supplementary material online, *Figure S7B*). Together, these results indicate that the VEGFA-MEF2 regulatory pathway is over-activated within both the developing endocardial-derived and SV-derived coronary EC populations in response to a hypoxia-mimic myocardial environment. However, while this results in expanded ectopic endocardial sprouting, the sustained VEGFA-MEF2 activity in the SV-derived plexus did not appear to lead to expanded SV-derived coronary sprouting.

To better examine the role of hypoxia in SV-derived sprouting, we next investigated the expression of the SOXF-driven Notch1 + 16 enhancer (*Figure 5A*) in *Phd2^fl/fl^;Nkx2.5-Cre* embryonic hearts relative to controls. In the heart, this SOXF-driven enhancer becomes active in the SV-derived vascular plexus as it sprouts from the SV at around E11.75 and remains active as this plexus expands to cover the dorsal ventricle at E13.5 before slowly becoming specific to arterial endothelium by birth (*Figure 5* and ^13^). This expression closely mimics that of SOX17 (see Supplementary material online, *Figure S4*,^8,37^). As expected, strong Notch1 + 16:*LacZ* activity was seen in the SV-derived plexus as it sprouts at E12.5-E13.5. However, the expression of the Notch1 + 16:*LacZ* transgene was not expanded in the SV-derived plexus in *Phd2^fl/fl^;Nkx2.5-Cre* hearts comparative to controls (*Figure 5B–D* and Supplementary material online, *Figure S8*). Additionally, we did not see any evidence of increased migration of Notch1 + 16:lacZ-positive vessels into the myocardium in *Phd2^fl/fl^;Nkx2.5-Cre* hearts (see Supplementary material online, *Figure S8C*). Although variations in the extent of SV-derived sprouting within and between litters at this stage could potentially mask minor differences, quantification of the area of the dorsal ventricles covered by Notch1 + 16:*LacZ*-positive vascular plexus at E12.5 and E13.5 found little difference between the hypoxia-mimic and control hearts, with a trend for reduced ventricular coverage by the SV-derived plexus (*Figure 5D*). Consistent with the Notch1 + 16:lacZ transgene, *Aplnr* expression in the SV-derived plexus also appeared similar in *Phd2^fl/fl^;Nkx2.5-Cre* hearts relative to littermate controls (*Figure 5G*). Notably, unlike for the MEF2-driven HLX-3 enhancer, there was no activation of the Notch1 + 16 enhancer in the regions beyond the SV-derived plexus on the dorsal ventricular surface, suggesting that the ectopic vessel growth detected here was not utilizing a SOXF-driven transcriptional pathway (*Figure 5D*). Indeed, by E15.5 we detected clear reduced, rather than increased, lateral growth of the SV-derived plexus along the surface of the ventricle in *Phd2^fl/fl^;Nkx2.5-Cre* hearts, with the right ventricle consistently showing less coverage by the SV-derived plexus in *Phd2^fl/fl^;Nkx2.5-Cre* hearts. This is most notable on the ventral aspect, where the SV-derived plexus can be seen extending around the lateral portion of the right ventricle on control *Phd2^fl/fl^* hearts, but was absent or reduced in *Phd2^fl/fl^;Nkx2.5-Cre* hearts (*Figure 5F* and Supplementary material online, *Figure S9*). Overall, these results are in stark contrast to expression of the angiogenic MEF2-driven enhancer at this timepoint, which was strongly increased (alongside increased blood islands) on the lateral portion of the right ventricle in *Phd2^fl/fl^;Nkx2.5-Cre* hearts.

In addition to the SV-derived plexus, in wildtype hearts the SOXF-driven Notch1 + 16:*LacZ* transgene is also active in a subset of ECs sprouting from the endocardium/septum onto the ventral surface along the septum from E12.5. This correlates with endogenous SOX17, whose expression in this region has previously been associated with ‘activated’ ECs sprouting from the endocardium/septum (see Supplementary material online, *Figure S4* and^8^). However, despite the association of hypoxia and VEGFA with increased endocardial-derived sprouting and SOX17-expressing activated ECs, and the increase in angiogenic ECs and MEF2-driven enhancer activity in *Phd2^fl/fl^;Nkx2.5-Cre* hearts, we found no significant qualitative or quantitative differences in Notch1 + 16:*LacZ* activity in the endocardial-sprout associated ventral vascular regions at E12.5 or E13.5 (*Figure 5E*). Collectively, these results suggest that stabilized HIFα and subsequent altered VEGFA expression in the heart differentially affected the MEF2-dependent and SOXF-dependent coronary regulatory pathways.

### 3.5 Arterial maturation is disrupted in *Phd2^fl/fl^;Nkx2.5-Cre* hearts

**Figure 6:**
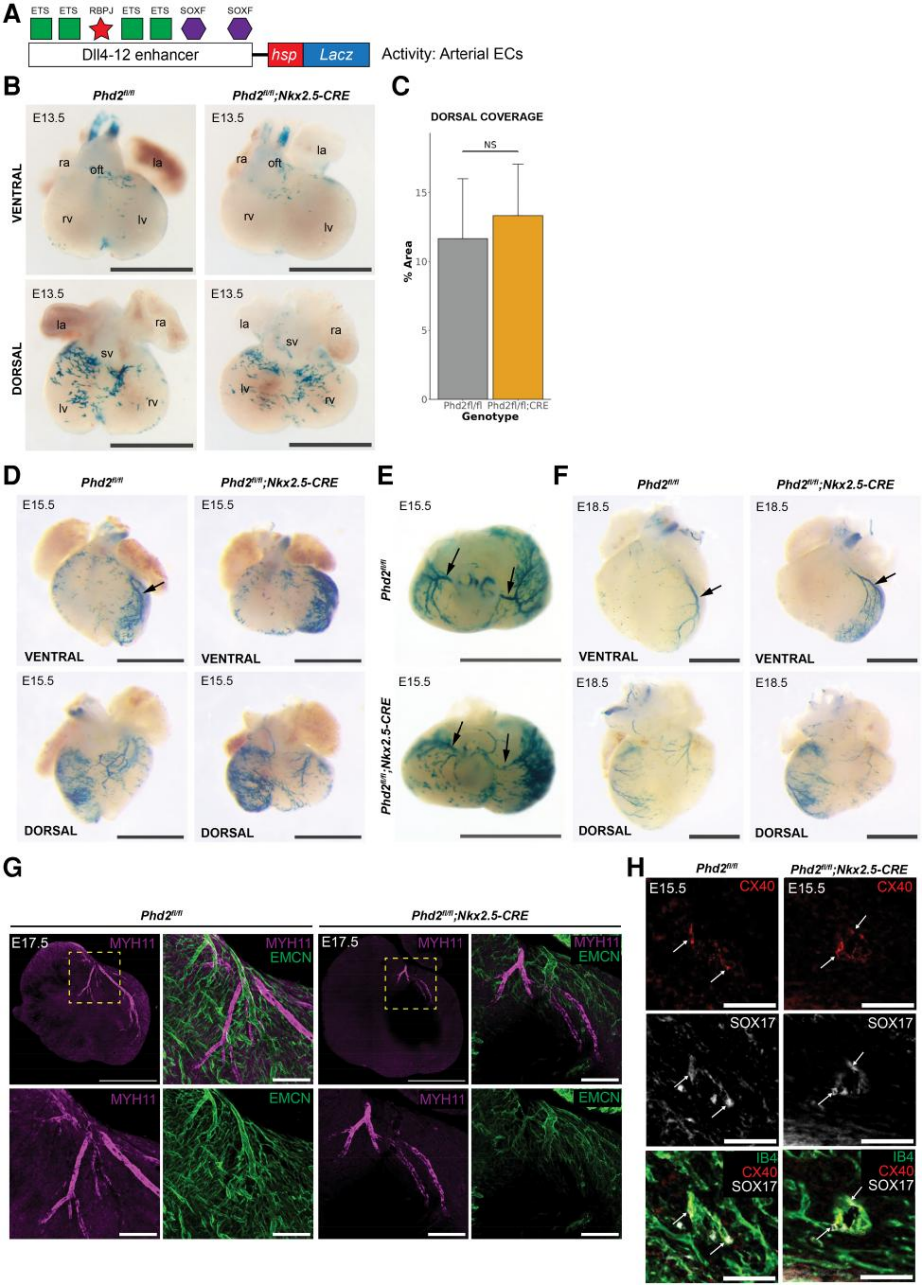
Arterial maturation, but not SOXF-dependent specification, is disrupted in hypoxia-mimic hearts. (*A*) Schematic of the Dll4-12:*LacZ* transgene, with the mouse Dll4-12 enhancer sequence cloned upstream of the hsp68 minimal promoter and *LacZ* reporter gene. Validated ETS, SOXF and RBPJ TF binding sites within the enhancer are represented by coloured shapes.^25^ (*B*) Representative ventral and dorsal views of E13.5 littermate hearts with Dll4-12:*LacZ* activity (blue X-gal stain) on *Phd2^fl/fl^* and *Phd2^fl/fl^;Nkx2.5-CRE* genetic backgrounds. N = 4 *Phd2^fl/fl^;Nkx2.5-CRE* hearts, all other hearts shown in Supplementary material online, *Figure S10*. Scale bars = 1 mm. ra, right atrium; la, left atrium; rv, right ventricle; lv, left ventricle; sv, sinus venosus; oft, outflow tract. (*C*) Quantification of Dll4-12:*LacZ*-positive area on dorsal aspect of the heart, as a percentage of total area. NS, not significant (unpaired *t* test), error bars show SEM, N = 4 *Phd2^fl/fl^;Nkx2.5-CRE* hearts and 5 *Phd2^fl/fl^* hearts. (*D*) E15.5 *Phd2^fl/fl^* and *Phd2^fl/fl^;Nkx2.5-CRE* hearts showing Dll4-12:*LacZ* activity. N = 8 *Phd2^fl/fl^;Nkx2.5-CRE* hearts, all other hearts shown in Supplementary material online, *Figure S11*, including heterozygous *Phd2^fl/+^;Nkx2.5-CRE* hearts as CRE controls. Scale bars = 1 mm. (*E*) Top view of E15.5 *Phd2^fl/fl^* and *Phd2^fl/fl^;Nkx2.5-CRE* hearts with atria removed, showing development of left and right coronary arteries (black arrows), additional hearts shown in Supplementary material online, *Figure S12A*. Scale bars = 1 mm. (*F*) E18.5 *Phd2^fl/fl^* and *Phd2^fl/fl^;Nkx2.5-CRE* hearts showing Dll4-12:*LacZ* activity. N = 5 *Phd2^fl/fl^;Nkx2.5-CRE* hearts, all other hearts shown in Supplementary material online, *Figure S12B*. Scale bars = 1 mm. (*G*) Wholemount immunostained E17.5 hearts showing smooth muscle marker MYH11, alongside venous EMCN. Additional biological repeats shown in Supplementary material online, *Figure S12C*. Scale bars = 1 mm for main images and 0.2 mm for magnified views. (*H*) Transverse sections through coronary arteries of *Phd2^fl/fl^* and *Phd2^fl/fl^;Nkx2.5-CRE* hearts showing expression of CX40 and SOX17, overlapping with the endothelial marker Isolectin GS-IB4 (IB4). Scale bars = 50 µm.

We next investigated whether early arterial development or patterning was altered by the increased HIFα stability and altered VEGFA expression in *Phd2^fl/fl^;Nkx2.5-Cre* hearts. ECs derived from both SV and endocardium contribute to the formation of the coronary arteries and cardiac veins.^12,14^ Coronary arterial differentiation begins at E12.5-E13.5 as a subset of ECs within both the SV-derived and endocardial-derived plexuses become pre-arterial ECs, reducing cell cycling and inducing the expression of some arterial genes (e.g. *Cxcr4, Efnb2, Dll4*). These pre-arterial ECs then gradually coalesce to form the coronary arteries, with clearly discernible arterial structures seen by E15.5.^11–13^ Analysis of similar arterial transitions in mouse retinal models have implicated hypoxia/VEGFA-induced angiogenic ECs as an essential precursor for this transition, alongside active Notch1 and high expression of SOX17 and DLL4.^3,13,38^ Supporting this, our own previous research identified an arterial enhancer for *Dll4* (named Dll4-12), regulated by a combination of VEGFA, SOXF, and NOTCH signalling and first active in pre-arterial ECs as they become specified (*Figure 6A*).^13,25^

In wildtype hearts, the Dll4-12:*LacZ* transgene becomes active in pre-arterial ECs at approximately E13.5, remaining specifically expressed in arterial ECs as they migrate and coalesce into mature arterial networks during later embryonic development and into the neonatal stages (*Figure 6B–F* and Supplementary material online, *Figures S10*–*S13*^13^). While our scRNA-seq detected a slight increase in the ratio of pre-arterial ECs in *Phd2^fl/fl^;Nkx2.5-Cre* hearts at E13.5 (*Figure 3E*), we did not see significant differences in Dll4-12:*LacZ* transgene activity in *Phd2^fl/fl^;Nkx2.5-Cre* hearts compared to littermate *Phd2^fl/fl^* controls at E13.5 (*Figure 6B, C* and Supplementary material online, *Figure S10*). However, increased Dll4-12:*LacZ* activity was clearly evident in *Phd2^fl/fl^;Nkx2.5-Cre* hearts by E15.5 (*Figure 6D, E* and Supplementary material online, *Figures S11* and *S12*), with the need for β-gal accumulation potentially explaining the delay relative to the scRNA-seq results. Strikingly, this increased Dll4-12:*LacZ* activity occurred alongside a reduced number of differentiated arterial structures in *Phd2^fl/fl^;Nkx2.5-Cre* hearts. For example, the developing left anterior descending artery was consistently under-developed or missing in *Phd2^fl/fl^;Nkx2.5-Cre* hearts when compared with littermate controls at both E15.5 and E18.5, whilst the intensity of Dll4-12:*LacZ* in surrounding ECs was much stronger (*Figure 6D–F* and Supplementary material online, *Figures S11* and *S12*). This arterial truncation defect was also seen after wholemount immunostaining for MYH11, which marks the smooth muscle layer of mature arterial structures (*Figure 6G* and Supplementary material online, *Figure S12D*), although both *Phd2^fl/fl^;Nkx2.5-Cre* and littermate control arteries correctly expressed canonical arterial EC markers CX40 and SOX17 (*Figure 6H*). The dorsal side of the E15-E18 heart also had fewer Dll4-12:*LacZ*-positive arterial ECs organizing into larger vessels, with greater numbers of Dll4-12:*LacZ*-positive ECs remaining in more discontinuous plexus (*Figure 6D–F* and Supplementary material online, *Figures S11* and *S12*). Overall, this data suggests that while early fate allocation of arterial ECs was not overly impacted by stabilized HIF/increased VEGFA levels, the ability of these ECs to migrate and coalesce into mature arteries was compromised.

The arterial patterning defects seen in *Phd2^fl/fl^;Nkx2.5-Cre* hearts did not persist into postnatal life. Hearts collected from surviving pups at postnatal days (P)6 and P8 similarly expressed Dll4-12:*LacZ* in mature coronary arteries in both *Phd2^fl/fl^;Nkx2.5-Cre* and control *Phd2^fl/fl^* hearts (see Supplementary material online, *Figure S13*), indicating that surviving *Phd2^fl/fl^;Nkx2.5-Cre* pups are able to compensate for the developmental disruption and form a mature vascular network that enables survival. Post-natal recovery from embryonic coronary vascular defects has been previously reported in *Apj^EC/EC^* mice, in this case driven by increased endocardial-derived sprouting compensating for defects in SV-derived coronary vessels.^8^ Endocardial-derived sprouting compensation may also explain the ability of these *Phd2^fl/fl^;Nkx2.5-Cre* mice to recover their coronary vasculature. Additionally, the increased haemodynamic burden applied to post-natal hearts may contribute to the stabilization of the coronary arterial tree in mice that survive to this stage, as fluid shear stress is known to play a role in arterial maintenance and remodelling.^39,40^

In conclusion, our results suggest that early arterial EC differentiation from the plexus is not significantly impacted in a HIFα-stabilized high-VEGFA myocardial environment, despite increased angiogenesis and higher numbers of pre-arterial ECs. However, coronary artery maturation, patterning and remodelling are disrupted by this altered microenvironment. While this results in smaller and fewer coronary arteries by the end of gestation, this can be compensated for after birth in surviving mice.

### 3.6 Venous patterning is affected in *Phd2^fl/fl^;Nkx2.5-Cre* hearts

**Figure 7:**
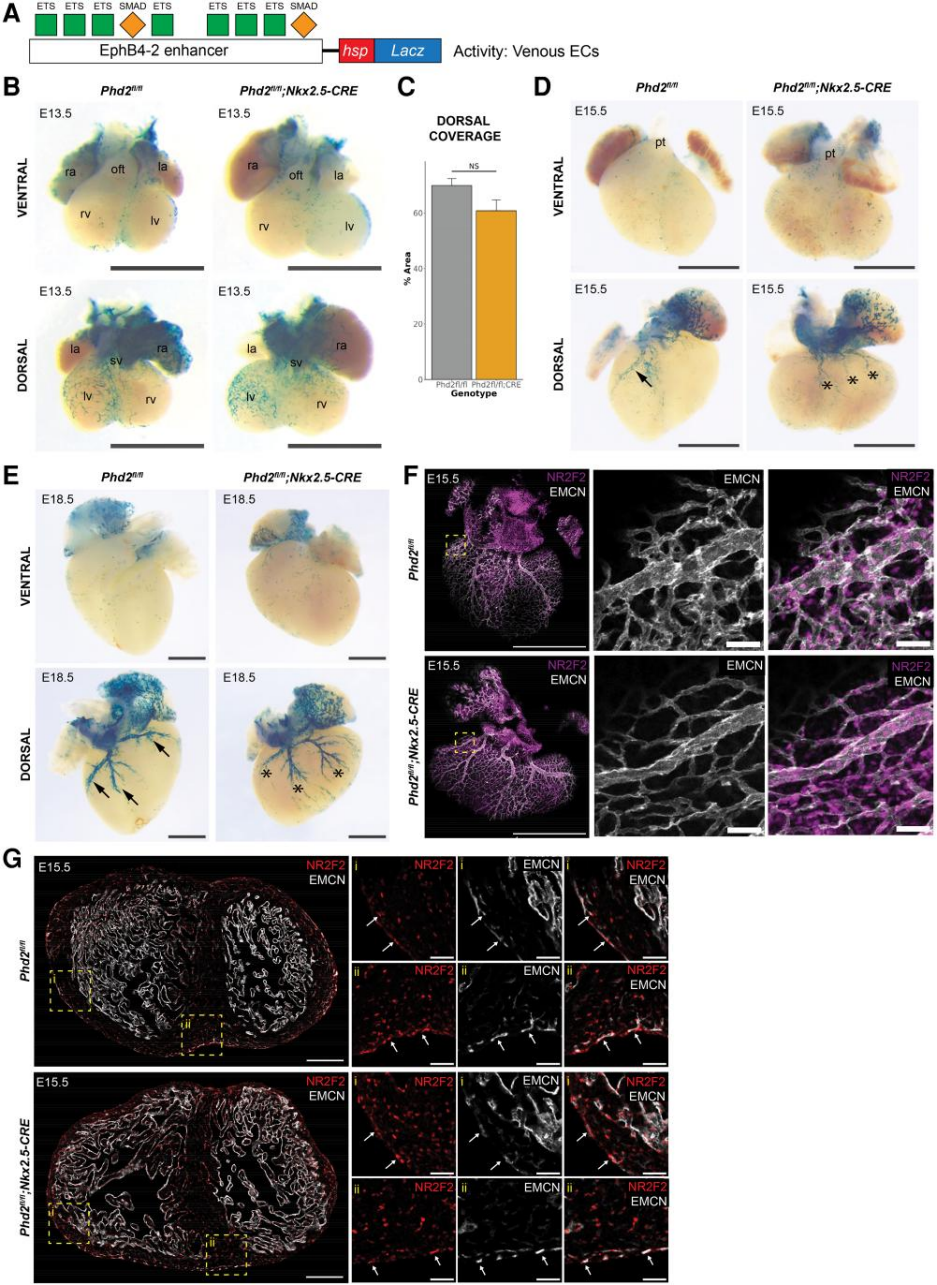
Venous specification and patterning in hypoxia-mimic myocardium. (*A*) Schematic of the EphB4-2:*LacZ* transgene, with the mouse EphB4-2 enhancer sequence cloned upstream of the hsp68 minimal promoter and *LacZ* reporter gene. Validated ETS and SMAD TF binding sites within the enhancer are represented by coloured shapes.^26^ (*B*) Representative E13.5 littermate hearts with EphB4-2:*LacZ* activity (blue X-gal stain) on *Phd2^fl/fl^* and *Phd2^fl/fl^;Nkx2.5-CRE* genetic backgrounds. *N* = 6 *Phd2^fl/fl^;Nkx2.5-CRE* hearts, all other hearts shown in Supplementary material online, *Figure S14A*. Scale bars = 1 mm. ra, right atrium; la, left atrium; rv, right ventricle; lv, left ventricle; sv, sinus venosus; oft, outflow tract. (*C*) Quantification of the extension of the EphB4-2:*LacZ*-positive SV-derived vascular plexus as a percentage of the total dorsal area of *Phd2^fl/fl^* and *Phd2^fl/fl^;Nkx2.5-CRE* hearts at E13.5. NS, not significant (unpaired *t* test), error bars show SEM, N = 6 hearts for each genotype. (*D*) E15.5 *Phd2^fl/fl^* and *Phd2^fl/fl^;Nkx2.5-CRE* hearts showing EphB4:*LacZ* activity—major cardiac veins are highlighted with black arrows, with truncated and misdirected veins highlighted with asterisks. N = 8 *Phd2^fl/fl^;Nkx2.5-CRE* hearts, all other hearts shown in Supplementary material online, *Figure S14B*. Scale bars = 1 mm. pt = pulmonary trunk. (*E*) E18.5 *Phd2^fl/fl^* and *Phd2^fl/fl^;Nkx2.5-CRE* hearts showing EphB4-2:*LacZ* activity. N = 4 *Phd2^fl/fl^;Nkx2.5-CRE* hearts, all other hearts shown in Supplementary material online, *Figure S15A*. Scale bars = 1 mm. (*F*) Wholemount E15.5 *Phd2^fl/fl^* and *Phd2^fl/fl^;Nkx2.5-CRE* hearts immunostained for venous markers EMCN and NR2F2. Additional biological repeats are shown in Supplementary material online, *Figure S15B*. Scale bars = 1 mm for main images and 0.2 mm for magnified views. (*G*) Transverse sections through E15.5 *Phd2^fl/fl^* and *Phd2^fl/fl^;Nkx2.5-CRE* hearts immunostained for EMCN and NR2F2, with arrows highlighting cardiac veins. Scale bars = 200 µm for main images and 50 µm for magnified views.

Finally, we investigated whether differentiation and maturation of the cardiac veins was affected in our *Phd2^fl/fl^;Nkx2.5-Cre* hearts. Similar to coronary arterial ECs, cardiac vein ECs form from both SV-derived and endocardial-derived ECs via differentiation, although this process occurs slightly later and was therefore not captured by our scRNA-seq analysis. Although the SV ECs from which the dorsal plexus is formed are venous in origin, transcriptomic analysis suggests that these ECs first de-differentiate before re-differentiating into venous coronary ECs.^12^ BMP-SMAD signalling has been implicated in the regulation of vein specification in both systemic and coronary vasculature,^13,26^ in part by activating expression of *Coup-TFII* (*Nr2f2*) and *Ephb4*, both key regulators of venous identity.^12^ Here, we used the expression of the BMP-SMAD-driven Ephb4-2:*LacZ* transgene line to investigate the effects of a hypoxia-mimic myocardium on this developmental pathway (*Figure 7A*).^26,41^ As expected due to its venous origin, the Ephb4-2:*LacZ* transgene is initially strongly expressed in the SV and in the SV-derived plexus. Transgene activity switches off in the plexus around E12.5-E13.0 as the ECs de-differentiate and lose venous identity, although some X-gal staining remains at E13.5 due to the half-life of β-gal activity (*Figure 7B* and^13^). Here, we found that Ephb4-2:*LacZ* activity was similar in E13.5 *Phd2^fl/fl^;Nkx2.5-Cre* hearts and controls, with X-gal staining largely restricted to similarly migrated SV-derived plexuses in both conditions with no statistical differences in the coverage of the dorsal aspect (*Figure 7B, C* and Supplementary material online, *Figure S14A*). This supports our observation that SV-derived sprouting was not expanded by alterations to HIFα stability/increased VEGFA expression and suggests that the ectopic angiogenesis/MEF2 pathway activation seen beyond the plexus did not involve activation of BMP-SMAD signalling.

We next looked at E15.5, a timepoint by which definitive venous coronary vessels are formed. At this stage, both *Phd2^fl/fl^;Nkx2.5-Cre* and *Phd2^fl/fl^* control hearts showed selective activity of the Ephb4-2:*LacZ* transgene in the newly formed cardiac veins, suggesting that the switch to venous fate driven by BMP-SMAD is not ablated by hypoxic-like conditions in the myocardium (*Figure 7D* and Supplementary material online, *Figure S14B*). However, the cardiac veins in *Phd2^fl/fl^;Nkx2.5-Cre* hearts were truncated and mis-patterned, and the large cardiac vein seen on the dorsal aspect of the left ventricle of littermate control hearts was absent in all *Phd2^fl/fl^;Nkx2.5-Cre* hearts. In addition to expression in the differentiating cardiac veins, Ephb4-2:*LacZ* transgene activity was increased on the ventral aspect of both ventricles in all *Phd2^fl/fl^;Nkx2.5-Cre* hearts (*Figure 7D* and Supplementary material online, *Figure S14B*). Whilst our scRNAseq did not find increased numbers of mature vein ECs at E13.5, it did find an increase in *Nr2f2*-expressing capillaries, which may correlate to this later ectopic Ephb4-2:*LacZ* expression. Ectopic transgene activation also occurred at the same regions that experienced increased VEGFA-MEF2 activity in *Phd2^fl/fl^;Nkx2.5-Cre* (*Figure 4I*), suggesting that the ectopic sprouting vessels from the endocardium may result in upregulation of vein-associated genes in response to hypoxia.

Increased Ephb4-2:*LacZ* expression around the pulmonary trunk in a significant subset of *Phd2^fl/fl^;Nkx2.5-Cre* hearts relative to controls also indicates increased formation of small cardiac veins in this region (*Figure 7* and Supplementary material online, *Figures S14* and *S15*). By E18.5, mature EphB4:*LacZ*-expressing cardiac veins were seen in both *Phd2^fl/fl^;Nkx2.5-Cre* and control hearts (*Figure 7E* and Supplementary material online, *Figure S15A*). However, the branching pattern was altered. In *Phd2^fl/fl^* hearts, the large cardiac veins extending towards the apex were predominantly located on the left ventricle, whilst in *Phd2^fl/fl^;Nkx2.5-Cre* hearts these veins extended further towards the right ventricle. However, the ectopic and disparate activity of the enhancer seen on the ventral aspect at E15.5 was largely gone, suggesting this was a transient upregulation of the pathway that does not produce additional cardiac veins (*Figure 7E* and Supplementary material online, *Figure S15A*). Immunostaining for the venous identity genes EMCN and NR2F2 further confirmed the formation of cardiac veins throughout the ventricles in both *Phd2^fl/fl^;Nkx2.5-Cre* and control hearts. Similarly, this analysis also found increased branching and altered venous patterning on the dorsal aspects of *Phd2^fl/fl^;Nkx2.5-Cre* hearts relative to controls (*Figure 7F, G* and Supplementary material online, *Figure S15B*).

Together, these results indicate that hypoxic-like myocardium does not inhibit activity of the BMP4-SMAD1/5 pathway during acquisition of cardiac venous identity, and can transiently drive increased and ectopic transgene activity. However, these hypoxia-mimic conditions lead to disruption to the patterning of the cardiac veins, potentially as a consequence of the thin myocardial wall seen in *Phd2^fl/fl^;Nkx2.5-Cre* hearts.

## 4. Discussion

The link between a hypoxic microenvironment, VEGFA and vascular growth in the systemic vasculature is well established. However, the exact mechanisms by which hypoxia, and the resulting stabilization of HIFα factors, influence coronary vessel growth in the heart have been challenging to ascertain. These are important pathways to understand: hypoxia is an immediate and damaging consequence of myocardial infarction (MI), yet neovascular growth in the ischaemic adult heart is insufficient.

The research here adds to a growing body of literature linking myocardial hypoxia specifically to endocardial-derived coronary vascularization. Wu *et al*.^6^ built on observations of hypoxia-dependent myocardial VEGFA gradients^42^ to demonstrate a direct link between VEGFA-VEGFR2 signalling and endocardial-derived angiogenesis. Supporting this, Sharma *et al*.^8^ later linked the expansion of endocardial-derived coronary angiogenesis that occurs in response to disrupted SV-derived coronary vasculature growth to increased myocardial hypoxia. Our previous studies of the transcriptional link between VEGF signalling and angiogenic gene expression have also indicated a direct connection between hypoxia and endocardial sprouting, with strong activity of VEGFA-MEF2-driven angiogenic enhancers occurring in regions associated with endocardial-derived sprouting.^13,23^ Here we used a genetic model to mimic hypoxia through ectopic stabilization of HIFα in the myocardium. This enabled us to study the consequences of hypoxia without depleting any of the vascular beds in the developing heart, although this approach could not assess the potential role(s) of endothelial-autonomous hypoxia sensing on coronary vessels. Our results found increased levels of VEGFA and other hypoxia-linked paracrine factors in these hearts, alongside increased pro-angiogenic gene expression and expanded activity of the VEGFA-MEF2 angiogenic regulatory pathway in a pattern indicative of increased endocardial-derived sprouting. These observations further emphasize a direct and specific link between hypoxia and endocardial coronary vessel sprouting.

It is still not clear how distinct angiogenic sprouting from the endocardium is achieved in either physiological or hypoxic/VEGFA-abundant environments. Both SV-derived and endocardial-derived coronary sprouting occur via angiogenesis by definition, with new vessels forming from existing ones. Whilst previous work implicates VEGFC and ELABELA as the main signalling ligands for SV-derived coronary angiogenesis,^5^ the SV-derived plexus expresses many genes associated with VEGFA-driven angiogenesis in other vascular beds (e.g. *Esm1*, *Apln*, *Pdgfb*, *Sox7, Sox17*, and *Sox18*^8,9,37,43–45^). Further, our previous work also found transient activity of the VEGFA-MEF2-driven angiogenic pathway at the leading edge of the SV-derived plexus as it migrated across the dorsal aspect, indicating that SV-derived sprouting may have some VEGFA dependency. However, we found no evidence of increased SV-derived plexus sprouting in our *Phd2^fl/fl^;Nkx2.5-Cre* hearts. Although we saw expanded activity of the VEGFA-MEF2-driven transgene on the dorsal apex and lateral regions, this was not in a pattern indicative of an expanded SV-derived plexus. Instead, cross-sectional analysis demonstrated that this increased activity was centred on the endocardium: in control hearts, activity of the VEGF-MEF2 driven pathway in the endocardium was restricted to the regions around the septum, whereas in *Phd2^fl/fl^;Nkx2.5-Cre* hearts this expression was expanded to much of the endocardium and into related vessel sprouts. This lack of hypoxic-impact on the SV-derived plexus was further emphasized by the activity of the Notch1 + 16 and Ephb4-2 enhancers, active in the early SV-derived plexus and not expanded in *Phd2^fl/fl^;Nkx2.5-Cre* hearts. Analysis of an alternate genetic model of hypoxia, using *Mef2c-Cre* to delete VHL and stabilize HIFα, observed both increased endocardial-derived angiogenesis alongside a reduced SV-derived plexus.^36^ While they hypothesized this may be due to a premature migration of the SV-derived vessels into the myocardium, this does not match the pattern of expanded VEGFA expression in these hearts and we saw no evidence of this. Together, our data instead suggests that the genetic pathways regulating angiogenesis from the SV are independent from hypoxia and cognate expanded VEGFA.

Our observations closely align with a previous model in which physiological triggers in the heart exclusively affect endocardial-derived sprouting.^8^ However, although earlier research implicated SOX17 as the specific transcriptional regulator of hypoxia-driven endocardial sprouting,^8^ SOX17 is expressed both in endocardial-derived sprouting ECs and throughout the SV-derived plexus (^8^and this paper). It is therefore highly likely that additional transcription factors are involved in this pathway, permitting SOX17 and other SOXF factors to transactivate differential gene expression patterns in response to signals from either the epicardial surface (in SV-derived vessels) or from within the myocardium (in endocardial-derived vessels). This is supported by our observation that the SOXF-dependent Notch1 + 16 enhancer was not expanded in our *Phd2^fl/fl^;Nkx2.5-Cre* hearts, despite the expanded SOX17 expression seen in the hypoxia-mimic endocardial-derived plexus.^8^

Hypoxia and subsequent increased VEGFA levels have been closely linked with arterial gene expression and differentiation in other vascular beds.^40^ Although we did not detect a clear difference in genes associated with arterio-venous identity in our transcriptomic analysis, there were moderate shifts in the proportions of coronary ECs towards the pre-arterial clusters and away from venous clusters. This correlated with an observed increase in Dll4-12:*LacZ*-positive ECs away from defined vessels in E15.5 and E18.5 *Phd2^fl/fl^;Nkx2.5-Cre* hearts, potentially reflecting increased numbers of pre-arterial or arterial ECs. Although we did not detect obvious differences in Dll4-12:*LacZ*-positive pre-arterial ECs at E13.5, the stage in which they first differentiate, there was considerable variation in expression across litters at this time-point which could have masked small differences. Interestingly, the increase in Dll4-12:*LacZ*-positive ECs in later stage *Phd2^fl/fl^;Nkx2.5-Cre* hearts correlated with a clear reduction in mature coronary arteries. This indicates that while the differentiation of venous ECs into pre-arterial ECs is not significantly influenced by increased hypoxia and subsequently higher VEGFA levels, the ability of arterial-fated ECs to migrate and coalesce into mature arteries is compromised in the hypoxic and hypoxia-like environments in which HIFα is stabilized.

The exact mechanism of how and why the SV-derived endothelial plexus does not expand in response to hypoxic stimuli may be crucial to our understanding of the supressed neovascular response following MI. Lineage tracing studies have confirmed angiogenic sprouting from pre-existing coronary ECs makes a significant contribution to the neovascular response^46^ yet our previous work found that the VEGFA-MEF2 pathway was not active in the blood vessels surrounding the infarction in a mouse model of MI.^13^ It has also been reported that whilst coronary ECs transiently activate Apelin in response to VEGF in the adult heart, they fail to proliferate and give rise to new vessels, highlighting a reduced angiogenic potential of the myocardium compared with skeletal muscle.^18^ The underlying cause of this difference has not been elucidated, but it has been proposed to be a protective mechanism against tumour angiogenesis within the heart. The lack of MEF2-driven angiogenic gene expression has also been associated with potential HDAC-mediated inhibition of MEF2 activity: increased HDAC activity has been reported in the murine heart post-MI,^47^ and anti-HDAC treatment after MI results in improvements in neovascularization and cardiac function.^48^ A better understanding of why SV-derived vessels are not sensitive to hypoxia, and why the hypoxia-responsive VEGFA-MEF2 angiogenic pathway is not activated in the ischaemic adult heart, would therefore be hugely beneficial in the search for therapeutic interventions to improve neovascularization post-MI.

## Supplementary Material

cvag124_Supplementary_Data

## Data Availability

The data underlying this article are available in GEO, accession number GSE292395, at https://www.ncbi.nlm.nih.gov/geo.

## References

[1] Gerhardt H, Golding M, Fruttiger M, Ruhrberg C, Lundkvist A, Abramsson A, Jeltsch M, Mitchell C, Alitalo K, Shima D, Betsholtz C. VEGF guides angiogenic sprouting utilizing endothelial tip cell filopodia. J Cell Biol 2003;161:1163–1177. 12810700 10.1083/jcb.200302047 PMC2172999

[2] Potente M, Gerhardt H, Carmeliet P. Basic and therapeutic aspects of angiogenesis. Cell 2011;146:873–887. 21925313 10.1016/j.cell.2011.08.039

[3] Pitulescu ME, Schmidt I, Giaimo BD, Antoine T, Berkenfeld F, Ferrante F, Park H, Ehling M, Biljes D, Rocha SF, Langen UH, Stehling M, Nagasawa T, Ferrara N, Borggrefe T, Adams RH. Dll4 and notch signalling couples sprouting angiogenesis and artery formation. Nat Cell Biol 2017;19:915–927. 28714968 10.1038/ncb3555

[4] Katz TC, Singh MK, Degenhardt K, Rivera-Feliciano J, Johnson RL, Epstein JA, Tabin CJ. Distinct compartments of the proepicardial organ give rise to coronary vascular endothelial cells. Dev Cell 2012;22:639–650. 22421048 10.1016/j.devcel.2012.01.012 PMC3306604

[5] Chen HI, Sharma B, Akerberg BN, Numi HJ, Kivelä R, Saharinen P, Aghajanian H, McKay AS, Bogard PE, Chang AH, Jacobs AH, Epstein JA, Stankunas K, Alitalo K, Red-Horse K. The sinus venosus contributes to coronary vasculature through VEGFC-stimulated angiogenesis. Development 2014;141:4500–4512. 25377552 10.1242/dev.113639 PMC4302936

[6] Wu B, Zhang Z, Lui W, Chen X, Wang Y, Chamberlain AA, Moreno-Rodriguez RA, Markwald RR, O’Rourke BP, Sharp DJ, Zheng D, Lenz J, Baldwin HS, Chang C-P, Zhou B. Endocardial cells form the coronary arteries by angiogenesis through myocardial-endocardial VEGF signaling. Cell 2012;151:1083–1096. 23178125 10.1016/j.cell.2012.10.023 PMC3508471

[7] Red-Horse K, Ueno H, Weissman IL, Krasnow MA. Coronary arteries form by developmental reprogramming of venous cells. Nature 2010;464:549–553. 20336138 10.1038/nature08873 PMC2924433

[8] Sharma B, Ho L, Ford GH, Chen HI, Goldstone AB, Woo YJ, Quertermous T, Reversade B, Red-Horse K. Alternative progenitor cells compensate to rebuild the coronary vasculature in Elabela- and Apj-deficient hearts. Dev Cell 2017;42:655–666.e3. 28890073 10.1016/j.devcel.2017.08.008 PMC5895086

[9] Cano E, Schwarzkopf J, Kanda M, Lindberg EL, Hollfinger I, Pogontke C, Braeuning C, Fischer C, Hübner N, Gerhardt H. Intramyocardial sprouting tip cells specify coronary arterialization. Circ Res 2024;135:671–684. 39092506 10.1161/CIRCRESAHA.124.324868 PMC11361357

[10] McCracken IR, Dobie R, Bennett M, Passi R, Beqqali A, Henderson NC, Mountford JC, Riley PR, Ponting CP, Smart N, Brittan M, Baker AH. Mapping the developing human cardiac endothelium at single cell resolution identifies MECOM as a regulator of arteriovenous gene expression. Cardiovasc Res 2022;118:2960–2972. 35212715 10.1093/cvr/cvac023 PMC9648824

[11] Raftrey B, Williams IM, Coronado PER, Fan X, Chang AH, Zhao M, Roth R, Trimm E, Racelis R, D’Amato G, Phansalkar R, Nguyen A, Chai T, Gonzalez KM, Zhang Y, Ang LT, Loh KM, Bernstein D, Red-Horse K. Dach1 extends artery networks and protects against cardiac injury. Circ Res 2021;129:702–716. 34383559 10.1161/CIRCRESAHA.120.318271 PMC8448957

[12] Su T, Stanley G, Sinha R, D’Amato G, Das S, Rhee S, Chang AH, Poduri A, Raftrey B, Dinh TT, Roper WA, Li G, Quinn KE, Caron KM, Wu S, Miquerol L, Butcher EC, Weissman I, Quake S, Red-Horse K. Single-cell analysis of early progenitor cells that build coronary arteries. Nature 2018;559:356–362. 29973725 10.1038/s41586-018-0288-7 PMC6053322

[13] Payne S, Gunadasa-Rohling M, Neal A, Redpath AN, Patel J, Chouliaras KM, Ratnayaka I, Smart N, De-Val S. Regulatory pathways governing murine coronary vessel formation are dysregulated in the injured adult heart. Nat Commun 2019;10:3276. 31332177 10.1038/s41467-019-10710-2 PMC6646353

[14] D’Amato G, Phansalkar R, Naftaly JA, Fan X, Amir ZA, Coronado PER, Cowley DO, Quinn KE, Sharma B, Caron KM, Vigilante A, Red-Horse K. Endocardium-to-coronary artery differentiation during heart development and regeneration involves sequential roles of Bmp2 and Cxcl12/Cxcr4. Dev Cell 2022;57:2517–2532.e6. 36347256 10.1016/j.devcel.2022.10.007 PMC9833645

[15] Phansalkar R, Krieger J, Zhao M, Kolluru SS, Jones RC, Quake SR, Weissman I, Bernstein D, Winn VD, D’Amato G, Red-Horse K. Coronary blood vessels from distinct origins converge to equivalent states during mouse and human development. eLife 2021:10:. 10.7554/eLife.70246 PMC8673841 34910626

[16] Zhang H, Pu W, Li G, Huang X, He L, Tian X, Liu Q, Zhang L, Wu SM, Sucov HM, Zhou B. Endocardium minimally contributes to coronary endothelium in the embryonic ventricular free walls. Circ Res 2016;118:1880–1893. 27056912 10.1161/CIRCRESAHA.116.308749

[17] Lupu I-E, De Val S, Smart N. Coronary vessel formation in development and disease: mechanisms and insights for therapy. Nat Rev Cardiol 2020;17:790–806. 32587347 10.1038/s41569-020-0400-1

[18] Kocijan T, Rehman M, Colliva A, Groppa E, Leban M, Vodret S, Volf N, Zucca G, Cappelletto A, Piperno GM, Zentilin L, Giacca M, Benvenuti F, Zhou B, Adams RH, Zacchigna S. Genetic lineage tracing reveals poor angiogenic potential of cardiac endothelial cells. Cardiovasc Res 2021;117:256–270. 31999325 10.1093/cvr/cvaa012 PMC7797216

[19] Mazzone M, Dettori D, de Oliveira RL, Loges S, Schmidt T, Jonckx B, Tian Y-M, Lanahan AA, Pollard P, de Almodovar CR, Smet FD, Vinckier S, Aragonés J, Debackere K, Luttun A, Wyns S, Jordan B, Pisacane A, Gallez B, Lampugnani MG, Dejana E, Simons M, Ratcliffe P, Maxwell P, Carmeliet P. Heterozygous deficiency of PHD2 restores tumor oxygenation and inhibits metastasis via endothelial normalization. Cell 2009;136:839–851. 19217150 10.1016/j.cell.2009.01.020 PMC4037868

[20] Moses KA, DeMayo F, Braun RM, Reecy JL, Schwartz RJ. Embryonic expression of an Nkx2-5/Cre gene using ROSA26 reporter mice. Genesis 2001;31:176–180. 11783008 10.1002/gene.10022

[21] Madisen L, Zwingman TA, Sunkin SM, Oh SW, Zariwala HA, Gu H, Ng LL, Palmiter RD, Hawrylycz MJ, Jones AR, Lein ES, Zeng H. A robust and high-throughput Cre reporting and characterization system for the whole mouse brain. Nat Neurosci 2010;13:133–140. 20023653 10.1038/nn.2467 PMC2840225

[22] Soriano P . Generalized lacZ expression with the ROSA26 Cre reporter strain. Nat Genet 1999;21:70–71. 9916792 10.1038/5007

[23] Sacilotto N, Chouliaras KM, Nikitenko LL, Lu YW, Fritzsche M, Wallace MD, Nornes S, Garcia-Moreno F, Payne S, Bridges E, Liu K, Biggs D, Ratnayaka I, Herbert SP, Molnar Z, Harris AL, Davies B, Bond GL, Bou-Gharios G, Schwarz JJ, De Val S. MEF2 transcription factors are key regulators of sprouting angiogenesis. Genes Dev 2016;30:2297–2309. 27898394 10.1101/gad.290619.116 PMC5110996

[24] Chiang IK-N, Fritzsche M, Pichol-Thievend C, Neal A, Holmes K, Lagendijk A, Overman J, D’Angelo D, Omini A, Hermkens D, Lesieur E, Fossat N, Radziewic T, Liu K, Ratnayaka I, Corada M, Bou-Gharios G, Tam PPL, Carroll J, Dejana E, Schulte-Merker S, Hogan BM, Beltrame M, De Val S, Francois M. Correction: SoxF factors induce Notch1 expression via direct transcriptional regulation during early arterial development. Development 2017;144:3847–3848. 28619820 10.1242/dev.146241 PMC5536923

[25] Sacilotto N, Monteiro R, Fritzsche M, Becker PW, Sanchez-del-Campo L, Liu K, Pinheiro P, Ratnayaka I, Davies B, Goding CR, Patient R, Bou-Gharios G, De-Val S. Analysis of Dll4 regulation reveals a combinatorial role for Sox and Notch in arterial development. Proc Natl Acad Sci U S A 2013;110:11893–11898. 23818617 10.1073/pnas.1300805110 PMC3718163

[26] Neal A, Nornes S, Payne S, Wallace MD, Fritzsche M, Louphrasitthiphol P, Wilkinson RN, Chouliaras KM, Liu K, Plant K, Sholapurkar R, Ratnayaka I, Herzog W, Bond G, Chico T, Bou-Gharios G, De Val S. Venous identity requires BMP signalling through ALK3. Nat Commun 2019;10:453. 30692543 10.1038/s41467-019-08315-w PMC6349860

[27] Zheng GXY, Terry JM, Belgrader P, Ryvkin P, Bent ZW, Wilson R, Ziraldo SB, Wheeler TD, McDermott GP, Zhu J, Gregory MT, Shuga J, Montesclaros L, Underwood JG, Masquelier DA, Nishimura SY, Schnall-Levin M, Wyatt PW, Hindson CM, Bharadwaj R, Wong A, Ness KD, Beppu LW, Deeg HJ, McFarland C, Loeb KR, Valente WJ, Ericson NG, Stevens EA, Radich JP, Mikkelsen TS, Hindson BJ, Bielas JH. Massively parallel digital transcriptional profiling of single cells. Nat Commun 2017;8:14049. 28091601 10.1038/ncomms14049 PMC5241818

[28] McGinnis CS, Murrow LM, Gartner ZJ. DoubletFinder: doublet detection in single-cell RNA sequencing data using artificial nearest neighbors. Cell Syst 2019;8:329–337.e4. 30954475 10.1016/j.cels.2019.03.003 PMC6853612

[29] Wang F, Flanagan J, Su N, Wang L-C, Bui S, Nielson A, Wu X, Vo H-T, Ma X-J, Luo Y. RNAscope. J Mol Diagn 2012;14:22–29. 22166544 10.1016/j.jmoldx.2011.08.002 PMC3338343

[30] Shlush LI, Itzkovitz S, Cohen A, Rutenberg A, Berkovitz R, Yehezkel S, Shahar H, Selig S, Skorecki K. Quantitative digital in situ senescence-associated β-galactosidase assay. BMC Cell Biol 2011;12:16–16. 21496240 10.1186/1471-2121-12-16 PMC3101133

[31] Jaakkola P, Mole DR, Tian Y-M, Wilson MI, Gielbert J, Gaskell SJ, von Kriegsheim A, Hebestreit HF, Mukherji M, Schofield CJ, Maxwell PH, Pugh CW, Ratcliffe PJ. Targeting of HIF-α to the von Hippel-Lindau ubiquitylation Complex by O2-regulated prolyl hydroxylation. Science 2001;292:468–472. 11292861 10.1126/science.1059796

[32] Liu Y, Cox SR, Morita T, Kourembanas S. Hypoxia regulates vascular endothelial growth factor gene expression in endothelial cells: identification of a 5′ enhancer. Circ Res 1995;77:638–643. 7641334 10.1161/01.res.77.3.638

[33] Ramakrishnan S, Anand V, Roy S. Vascular endothelial growth factor signaling in hypoxia and inflammation. J Neuroimmune Pharmacol 2014;9:142–160. 24610033 10.1007/s11481-014-9531-7 PMC4048289

[34] Menendez-Montes I, Escobar B, Palacios B, Gómez MJ, Izquierdo-Garcia JL, Flores L, Jiménez-Borreguero LJ, Aragones J, Ruiz-Cabello J, Torres M, Martin-Puig S. Myocardial VHL-HIF signaling controls an embryonic metabolic switch essential for cardiac maturation. Dev Cell 2016;39:724–739. 27997827 10.1016/j.devcel.2016.11.012

[35] del Toro R, Prahst C, Mathivet T, Siegfried G, Kaminker JS, Larrivee B, Bréant C, Duarte A, Takakura N, Fukamizu A, Penninger J, Eichmann A. Identification and functional analysis of endothelial tip cell-enriched genes. Blood 2010;116:4025–4033. 20705756 10.1182/blood-2010-02-270819 PMC4314527

[36] Vitali HE, Kuschel B, Sherpa C, Jones BW, Jacob N, Madiha SA, Elliott S, Dziennik E, Kreun L, Conatser C, Bhetwal BP, Sharma B. Hypoxia regulate developmental coronary angiogenesis potentially through VEGF-R2- and SOX17-mediated signaling. Dev Dyn 2025;254:174–188. 39360476 10.1002/dvdy.750 PMC11810610

[37] González-Hernández S, Gómez MJ, Sanchez-Cabo F, Méndez-Ferrer S, Muñoz-Cánoves P, Isern J. Sox17 controls emergence and remodeling of nestin-expressing coronary vessels. Circ Res 2020;127:e252–e270. 32921258 10.1161/CIRCRESAHA.120.317121

[38] Tsang KM, Hyun JS, Cheng KT, Vargas M, Mehta D, Ushio-Fukai M, Zou L, Pajcini KV, Rehman J, Malik AB. Embryonic stem cell differentiation to functional arterial endothelial cells through sequential activation of ETV2 and NOTCH1 signaling by HIF1α. Stem Cell Reports 2017;9:796–806. 28781077 10.1016/j.stemcr.2017.07.001 PMC5599266

[39] Le Noble F, Kupatt C. Interdependence of angiogenesis and arteriogenesis in development and disease. Int J Mol Sci 2022;23:3879. 35409246 10.3390/ijms23073879 PMC8999596

[40] Chen D, Schwartz MA, Simons M. Developmental perspectives on arterial fate specification. Front Cell Dev Biol 2021;9:691335. 34249941 10.3389/fcell.2021.691335 PMC8269928

[41] Neal A, Nornes S, Louphrasitthiphol P, Sacilotto N, Preston MD, Fleisinger L, Payne S, De-Val S. ETS factors are required but not sufficient for specific patterns of enhancer activity in different endothelial subtypes. Dev Biol 2021;473:1–14. 33453264 10.1016/j.ydbio.2021.01.002 PMC8026812

[42] Wikenheiser J, Doughman Y, Fisher SA, Watanabe M. Differential levels of tissue hypoxia in the developing chicken heart. Dev Dyn 2006;235:115–123. 16028272 10.1002/dvdy.20499

[43] Zarkada G, Howard JP, Xiao X, Park H, Bizou M, Leclerc S, Künzel SE, Boisseau B, Li J, Cagnone G, Joyal JS, Andelfinger G, Eichmann A, Dubrac A. Specialized endothelial tip cells guide neuroretina vascularization and blood-retina-barrier formation. Dev Cell 2021;56:2237–2251.e6. 34273276 10.1016/j.devcel.2021.06.021 PMC9951594

[44] Tian X, Hu T, Zhang H, He L, Huang X, Liu Q, Yu W, He L, Yang Z, Zhang Z, Zhong TP, Yang X, Yang Z, Yan Y, Baldini A, Sun Y, Lu J, Schwartz RJ, Evans SM, Gittenberger-de Groot AC, Red-Horse K, Zhou B. Subepicardial endothelial cells invade the embryonic ventricle wall to form coronary arteries. Cell Res 2013;23:1075–1090. 23797856 10.1038/cr.2013.83 PMC3760626

[45] Kim K, Kim I-K, Yang JM, Lee E, Koh BI, Song S, Park J, Lee S, Choi C, Kim JW, Kubota Y, Koh GY, Kim I. Soxf transcription factors are positive feedback regulators of VEGF signaling. Circ Res 2016;119:839–852. 27528602 10.1161/CIRCRESAHA.116.308483

[46] He L, Huang X, Kanisicak O, Li Y, Wang Y, Li Y, Pu W, Liu Q, Zhang H, Tian X, Zhao H, Liu X, Zhang S, Nie Y, Hu S, Miao X, Wang QD, Wang F, Chen T, Xu Q, Lui KO, Molkentin JD, Zhou B. Preexisting endothelial cells mediate cardiac neovascularization after injury. J Clin Invest 2017;127:2968–2981. 28650345 10.1172/JCI93868 PMC5531398

[47] Granger A, Abdullah I, Huebner F, Stout A, Wang T, Huebner T, Epstein JA, Gruber PJ. Histone deacetylase inhibition reduces myocardial ischemia-reperfusion injury in mice. FASEB J 2008;22:3549–3560. 18606865 10.1096/fj.08-108548 PMC2537432

[48] Zhang L, Chen B, Zhao Y, Dubielecka PM, Wei L, Qin GJ, Chin YE, Wang Y, Zhao TC. Inhibition of histone deacetylase-induced myocardial repair is mediated by c-kit in infarcted hearts. J Biol Chem 2012;287:39338–39348. 23024362 10.1074/jbc.M112.379115 PMC3501087

