# Supplementary material for: Hypoxia differentially affects coronary vessel formation during heart development
Source: Cardiovasc Res. 2026 Jun 8;122(11):1487–505. doi: 10.1093/cvr/cvag124 (PMC13473972; doi:10.1093/cvr/cvag124)
Supplement: Supplementary file 1 [file cvag124_supplementary_data.pdf]

Payne *et al.* "Hypoxia differentially affects coronary vessel formation during heart development"

Supplementary Data

Tables S1–S4

Figures S1-S15

Supplementary Tables

Supplementary Table S1: Differentially expressed genes across all cells

Differential gene expression analysis results comparing all *Phd2$^{fl/fl}$;Nkx2.5-Cre* with control *Phd2$^{fl/fl}$* cells, showing 37 genes upregulated in *Phd2$^{fl/fl}$;Nkx2.5-Cre* cells and 8 genes downregulated in *Phd2$^{fl/fl}$;Nkx2.5-Cre* cells. Genes were included if adjusted p-value after multiple testing correction < 0.05 and log2 fold change > 0.2.

Gene	p_val	avg_log2FC	pct.1	pct.2	p_val_adj	Direction
Tnnt2	5.83E-07	0.769966	0.245	0.197	0.018815	Upregulated
Pgk1	2.75E-52	0.564618	0.816	0.718	8.86E-48	Upregulated
Mif	4.77E-34	0.527886	0.834	0.754	1.54E-29	Upregulated
Aldoa	1.16E-50	0.520576	0.846	0.745	3.74E-46	Upregulated
Tpi1	5.19E-42	0.502359	0.783	0.673	1.68E-37	Upregulated
Ldha	5.24E-39	0.490589	0.901	0.842	1.69E-34	Upregulated
Ptn	1.71E-11	0.46801	0.402	0.333	5.52E-07	Upregulated
Bnip3	8.76E-83	0.458984	0.295	0.12	2.83E-78	Upregulated
Gapdh	1.08E-21	0.430632	0.937	0.918	3.47E-17	Upregulated
Slc8a1	1.74E-11	0.425151	0.152	0.101	5.62E-07	Upregulated
Nexn	1.19E-09	0.394475	0.194	0.14	3.85E-05	Upregulated
Sorbs2	5.45E-18	0.385761	0.199	0.125	1.76E-13	Upregulated
Sparcl1	1.87E-35	0.380434	0.342	0.209	6.03E-31	Upregulated
Pkm	9.01E-36	0.37744	0.807	0.706	2.91E-31	Upregulated
Cryab	1.45E-06	0.33295	0.146	0.109	0.04686	Upregulated
Vcan	1.61E-08	0.315993	0.491	0.418	5.19E-04	Upregulated
Pgam1	2.39E-23	0.311196	0.787	0.701	7.70E-19	Upregulated
Cfh	2.23E-11	0.299935	0.66	0.619	7.20E-07	Upregulated
Igfbp3	2.73E-10	0.298002	0.25	0.188	8.83E-06	Upregulated
Lbh	8.02E-13	0.279292	0.384	0.298	2.59E-08	Upregulated
Slc16a3	5.16E-65	0.274954	0.249	0.101	1.67E-60	Upregulated
Pfkl	4.29E-55	0.259505	0.534	0.347	1.39E-50	Upregulated
Slc2a1	1.71E-39	0.253781	0.294	0.165	5.52E-35	Upregulated
Gpi1	3.69E-27	0.244252	0.782	0.669	1.19E-22	Upregulated
Dst	7.53E-37	0.243859	0.773	0.636	2.43E-32	Upregulated
Palld	1.17E-21	0.233799	0.372	0.265	3.78E-17	Upregulated
Glo1	1.17E-50	0.232038	0.702	0.535	3.78E-46	Upregulated
Fam162a	6.65E-10	0.223962	0.777	0.701	2.15E-05	Upregulated
Nrg1	2.36E-10	0.219786	0.391	0.319	7.60E-06	Upregulated
Cdh2	2.22E-24	0.216845	0.194	0.108	7.17E-20	Upregulated
Dsp	2.45E-35	0.21093	0.267	0.146	7.91E-31	Upregulated
Ldhb	9.33E-18	0.210919	0.357	0.256	3.01E-13	Upregulated
Ddx3y	6.37E-25	0.210709	0.291	0.19	2.06E-20	Upregulated
Gyg	1.17E-13	0.209222	0.462	0.359	3.78E-09	Upregulated
Atp2a2	1.24E-10	0.206864	0.791	0.704	4.01E-06	Upregulated
Tm4sf1	1.61E-10	0.202622	0.83	0.807	5.20E-06	Upregulated
Bgn	2.77E-12	0.201426	0.677	0.612	8.93E-08	Upregulated
Rps2	2.46E-131	-0.43798	0.968	0.971	7.93E-127	Downregulated

Hspa1b	2.14E-15	-0.40377	0.495	0.559	6.90E-11	Downregulated
Hspa1a	1.28E-12	-0.37829	0.489	0.55	4.12E-08	Downregulated
Xist	7.10E-26	-0.30238	0.549	0.689	2.29E-21	Downregulated
Hsph1	8.11E-09	-0.30149	0.717	0.717	2.62E-04	Downregulated
Jun	1.95E-11	-0.26893	0.925	0.931	6.30E-07	Downregulated
Igfbp5	5.19E-07	-0.24301	0.65	0.685	0.01674	Downregulated
Gm1673	1.40E-16	-0.20656	0.736	0.761	4.53E-12	Downregulated

Supplementary Table S2: Pathway analysis of genes upregulated in *Phd2$^{fl/fl}$;Nkx2.5-Cre* hearts

Gene-set enrichment analysis results from pathway analysis using g:Profiler on genes upregulated in hypoxic conditions at E13.5.

term_id	term_name	p_value
**GO:0006090**	pyruvate metabolic process	1.21E-08
**GO:0019674**	NAD metabolic process	2.30E-08
**GO:0006007**	glucose catabolic process	2.62E-08
**GO:0019320**	hexose catabolic process	1.78E-07
**GO:0046365**	monosaccharide catabolic process	2.46E-07
**GO:0019677**	NAD catabolic process	4.62E-07
**GO:0061718**	glucose catabolic process to pyruvate	4.62E-07
**GO:0061621**	canonical glycolysis	4.62E-07
**GO:0006735**	NADH regeneration	4.62E-07
**GO:0061620**	glycolytic process through glucose-6-phosphate	1.22E-06
**GO:0061615**	glycolytic process through fructose-6-phosphate	4.11E-06
**GO:0046496**	nicotinamide nucleotide metabolic process	4.77E-06
**GO:0019362**	pyridine nucleotide metabolic process	4.77E-06
**GO:0072524**	pyridine-containing compound metabolic process	5.26E-06
**GO:0032787**	monocarboxylic acid metabolic process	2.39E-05
**GO:0006006**	glucose metabolic process	2.75E-05
**GO:0006734**	NADH metabolic process	4.38E-05
**GO:0016052**	carbohydrate catabolic process	6.89E-05
**GO:0006096**	glycolytic process	9.52E-05
**GO:0019364**	pyridine nucleotide catabolic process	1.08E-04
**GO:0009137**	purine nucleoside diphosphate catabolic process	1.23E-04
**GO:0072526**	pyridine-containing compound catabolic process	1.23E-04
**GO:0046032**	ADP catabolic process	1.23E-04
**GO:0009181**	purine ribonucleoside diphosphate catabolic process	1.23E-04
**GO:0019318**	hexose metabolic process	1.52E-04
**GO:0009191**	ribonucleoside diphosphate catabolic process	1.79E-04
**GO:0009134**	nucleoside diphosphate catabolic process	2.01E-04
**GO:0046031**	ADP metabolic process	2.01E-04
**GO:0009135**	purine nucleoside diphosphate metabolic process	2.26E-04
**GO:0009179**	purine ribonucleoside diphosphate metabolic process	2.26E-04
**GO:0005996**	monosaccharide metabolic process	2.66E-04
**GO:0009185**	ribonucleoside diphosphate metabolic process	6.58E-04
**GO:0009132**	nucleoside diphosphate metabolic process	7.99E-04
**GO:0044282**	small molecule catabolic process	9.56E-04
**GO:0009154**	purine ribonucleotide catabolic process	9.65E-04
**GO:0006195**	purine nucleotide catabolic process	0.001159204
**GO:0072523**	purine-containing compound catabolic process	0.001268211
**GO:0046166**	glyceraldehyde-3-phosphate biosynthetic process	0.001510487
**GO:0009261**	ribonucleotide catabolic process	0.001512467
**GO:0019752**	carboxylic acid metabolic process	0.0018921
**GO:0043436**	oxoacid metabolic process	0.002109656
**GO:0006082**	organic acid metabolic process	0.002287316
**GO:0009166**	nucleotide catabolic process	0.002928081

**GO:1901292**	nucleoside phosphate catabolic process	0.00397931
**GO:0005975**	carbohydrate metabolic process	0.005614229
**GO:0019682**	glyceraldehyde-3-phosphate metabolic process	0.005882122
**GO:0046034**	ATP metabolic process	0.00846916
**GO:0006091**	generation of precursor metabolites and energy	0.012531191
**GO:0006163**	purine nucleotide metabolic process	0.01643671
**GO:0046434**	organophosphate catabolic process	0.019425481
**GO:0009205**	purine ribonucleoside triphosphate metabolic process	0.020521511
**GO:0046184**	aldehyde biosynthetic process	0.021375216
**GO:0072521**	purine-containing compound metabolic process	0.023290652
**GO:0009144**	purine nucleoside triphosphate metabolic process	0.025458806
**GO:0009199**	ribonucleoside triphosphate metabolic process	0.026559727
**GO:0006081**	cellular aldehyde metabolic process	0.029980448
**GO:1901136**	carbohydrate derivative catabolic process	0.039837804
**GO:0009141**	nucleoside triphosphate metabolic process	0.043308838
**GO:0009117**	nucleotide metabolic process	0.044876849
**KEGG:00010**	Glycolysis / Gluconeogenesis	3.13E-09
**KEGG:05230**	Central carbon metabolism in cancer	3.28E-04
**KEGG:01230**	Biosynthesis of amino acids	0.007055504
**KEGG:01200**	Carbon metabolism	0.008775507

Supplementary Table S3: Differentially expressed genes across cardiomyocyte clusters

Differential gene expression analysis results comparing *Phd2^fl/fl^;Nkx2.5-Cre* with control *Phd2^fl/fl^* cells from clusters 17 and 18, showing 11 genes upregulated in *Phd2^fl/fl^;Nkx2.5-Cre* cells and 29 genes downregulated in *Phd2^fl/fl^;Nkx2.5-Cre* cells. Due to the low total cell numbers (214), more stringent thresholds were applied, so genes were only included if adjusted p-value after multiple testing correction < 0.01 and log2 fold change > 1.2.

Gene	p_val	avg_log2FC	pct.1	pct.2	p_val_adj	Direction
**Nppa**	1.73E-13	7.433897	0.598	0.139	5.58E-09	Upregulated
**Slit2**	2.18E-10	5.14887	0.348	0.025	7.02E-06	Upregulated
**Gja5**	1.35E-09	3.634036	0.391	0.066	4.36E-05	Upregulated
**Angpt1**	5.39E-11	3.395358	0.457	0.082	1.74E-06	Upregulated
**Bmp10**	1.58E-07	3.370799	0.25	0.016	0.005094	Upregulated
**Mest**	9.82E-16	3.298591	0.837	0.41	3.17E-11	Upregulated
**Sema3a**	1.96E-07	2.97707	0.283	0.033	0.006317	Upregulated
**Cited1**	6.31E-10	2.726146	0.848	0.607	2.04E-05	Upregulated
**Cxcl12**	3.72E-16	2.706652	0.696	0.131	1.2E-11	Upregulated
**Fbln1**	2.7E-09	1.892686	0.641	0.279	8.73E-05	Upregulated
**Pam**	3.66E-10	1.383777	0.946	0.893	1.18E-05	Upregulated
**Ndufa4l2**	6.77E-10	-5.48367	0.011	0.361	2.19E-05	Downregulated
**Nrn1**	4.42E-09	-4.50403	0.022	0.352	0.000143	Downregulated
**Cox4i2**	3.38E-15	-4.46472	0.043	0.557	1.09E-10	Downregulated
**Tbx3**	4.16E-10	-4.44251	0.054	0.426	1.34E-05	Downregulated
**H2-Ab1**	7.39E-13	-3.90586	0.065	0.516	2.39E-08	Downregulated
**Rspo3**	7.92E-10	-3.85515	0.098	0.484	2.56E-05	Downregulated
**Egln3**	1.53E-11	-3.47096	0.087	0.516	4.95E-07	Downregulated
**Mgarp**	8.14E-14	-3.04962	0.12	0.623	2.63E-09	Downregulated
**Slc2a3**	1.24E-13	-2.7975	0.163	0.648	3.99E-09	Downregulated
**Plekha6**	3.55E-09	-2.42607	0.13	0.508	0.000115	Downregulated
**Tenm3**	1.74E-07	-2.33218	0.13	0.451	0.005606	Downregulated
**Bnip3**	2.55E-24	-1.99695	0.739	0.975	8.24E-20	Downregulated
**Plod2**	3.52E-11	-1.8763	0.348	0.738	1.14E-06	Downregulated
**Hk2**	3.07E-17	-1.87087	0.489	0.885	9.9E-13	Downregulated
**Slc2a1**	1.75E-14	-1.75615	0.62	0.934	5.66E-10	Downregulated
**Mt1**	5.55E-12	-1.70343	0.696	0.877	1.79E-07	Downregulated
**Loxl2**	9.94E-16	-1.69633	0.522	0.902	3.21E-11	Downregulated
**Ddah1**	1.17E-09	-1.5401	0.446	0.762	3.79E-05	Downregulated
**Map2k1**	2.83E-09	-1.50462	0.467	0.762	9.13E-05	Downregulated
**Egln1**	3.22E-19	-1.47108	0.837	0.951	1.04E-14	Downregulated
**Slc16a3**	2.52E-14	-1.38224	0.63	0.934	8.14E-10	Downregulated
**P4ha1**	3E-10	-1.38096	0.511	0.811	9.68E-06	Downregulated
**Pdk1**	3.53E-14	-1.33482	0.663	0.91	1.14E-09	Downregulated
**Pfkp**	9.76E-18	-1.29286	0.837	0.975	3.15E-13	Downregulated
**Marcks**	6.02E-09	-1.28445	0.598	0.902	0.000194	Downregulated
**Pgk1**	2.36E-24	-1.28041	0.978	1	7.61E-20	Downregulated

**Bnip3l**	2.82E-19	-1.22906	0.859	1	9.11E-15	Downregulated
**Jmjd6**	8.97E-10	-1.22263	0.598	0.869	2.9E-05	Downregulated
**Pgm1**	4.03E-16	-1.21454	0.772	0.959	1.3E-11	Downregulated

Supplementary Table S4: Top 10 up- and downregulated genes for each cluster of coronary ECs

Differentially expressed genes for each cluster were ranked by lowest p value or highest log2 fold change, and the top 10 up-and down-regulated genes from each list (with a value of >0.1 in both pct.1 and pct.2) are shown in these tables.

Cluster 0 – Capillaries (G1)

Ranked by p value

Gene	p_val	avg_log2FC	p_val_adj
Gde1	0.00011723	0.390050615	1
Leng1	0.00027847	0.272388824	1
Pdpk1	0.00031951	0.336365428	1
Nt5dc3	0.00036651	0.235225613	1
Kitl	0.00037917	0.653991355	1
Rbbp6	0.00050378	0.505719659	1
Wbp1l	0.00054522	0.298302515	1
S100a1	0.00065138	0.293045418	1
Ypel5	0.00098801	0.271896437	1
Zfp655	0.00119233	0.231546291	1
Rps2	8.57E-08	-0.49751659	0.002767
Ube2n	9.28E-06	-0.470006317	0.299742
Ipo11	0.00018916	-0.490648208	1
Ccdc102a	0.00072934	-0.323914943	1
Faf1	0.00128615	-0.277238636	1
Cep83	0.0017621	-0.391576322	1
Anapc1	0.00185757	-0.319123911	1
Ciao2b	0.00226197	-0.342031002	1
Rhot1	0.0026321	-0.269202788	1
Ciao2b	0.00226197	-0.342031002	1

Ranked by Log2FC

Gene	p_val	avg_log2FC	p_val_adj
Klf2	0.004689616	0.725003885	1
Kitl	0.000379165	0.653991355	1
Meg3	0.124498511	0.566063475	1
Hba-a1	0.287058091	0.538321035	1
Gm42418	0.019268232	0.510193699	1
Rbbp6	0.000503776	0.505719659	1
Arhgap21	0.001802563	0.435348407	1
Hist1h1b	0.974159252	0.434916695	1
Ddx3y	0.002848712	0.433708277	1
AY036118	0.055592311	0.431335085	1
Hbb-y	0.567172051	-1.853661647	1
Xist	0.003345007	-0.8270122	1
Cxcl1	0.062312016	-0.591125334	1
Rps2	8.57E-08	-0.49751659	0.002766635
Ipo11	0.000189157	-0.490648208	1
Ube2n	9.28E-06	-0.470006317	0.299741522
D10Wsu102e	0.151085311	-0.464868495	1
Igfbp3	0.831007544	-0.409757013	1
Phlda1	0.067020612	-0.404353045	1
Cep83	0.001762101	-0.391576322	1

Cluster 1 – Cycling capillaries (G2M) I

Ranked by p value

Gene	p_val	avg_log2FC	p_val_adj
Sugp1	6.20E-05	0.24203132	1
Txnip	0.000278716	0.3862888	1
Igf2	0.000303273	0.77567039	1
Sbds	0.000423675	0.349017827	1
Dstyk	0.000480631	0.190215073	1
Uggt2	0.000550018	0.166639742	1
Irx5	0.000695091	0.218097122	1
Kif1c	0.000874866	0.221223692	1
Socs5	0.000934012	0.179392902	1
H19	0.001052546	0.74331054	1
Rps2	4.68E-11	-0.646213199	1.51E-06
Dynll1	0.000145714	-0.331629136	1
Mtf2	0.000256111	-0.305828937	1
Snrpd3	0.000295245	-0.361624668	1
Tmem115	0.000376663	-0.193695617	1
Hmgn5	0.000432631	-0.439857748	1
Atp5f1	0.000679567	-0.309225893	1
**Rpl41**	0.000734855	-0.188007911	1
**Tax1bp3**	0.001223843	-0.329453953	1
**Rps25**	0.001255246	-0.180352122	1

Ranked by Log2FC

Gene	p_val	avg_log2FC	p_val_adj
Cdkn1c	0.005889196	1.049048185	1
Igf2	0.000303273	0.77567039	1
H19	0.001052546	0.74331054	1
Igfbp5	0.012441949	0.740472239	1
Gm42418	0.006286325	0.700558665	1
Hspa1b	0.35804158	0.662159348	1
AY036118	0.356136325	0.634708372	1
Igfbp3	0.377852289	0.578337023	1
Nts	0.489354823	0.557281456	1
Bmp2	0.028958109	0.516571197	1
Fabp4	0.004033197	-0.753906434	1
Rps2	4.68E-11	-0.646213199	1.51E-06
Hmgn5	0.000432631	-0.439857748	1
Khdrbs3	0.062533871	-0.404020934	1
Actn1	0.002926682	-0.378688117	1
Tma7	0.001730115	-0.37544394	1
Meox2	0.039765899	-0.365470792	1
Snrpd3	0.000295245	-0.361624668	1
Cdr2	0.002326507	-0.357215652	1
Myl12a	0.00153072	-0.353867451	1

Cluster 2 – Cycling capillaries (G2M) II

Ranked by p value

Gene	p_val	avg_log2FC	p_val_adj
Plvap	0.000205	0.388661	1
Mvb12b	0.000425	0.235572	1
Prpf3	0.000465	0.28021	1
Map3k1	0.000636	0.376033	1
Nol9	0.000706	0.323818	1
Lmf2	0.000782	0.301696	1
Celf1	0.001375	0.478418	1
Cdc42ep1	0.001447	0.378949	1
Nelfb	0.00145	0.254816	1
Inpp5a	0.001526	0.24337	1
Rps2	7.18E-08	-0.64573	0.002318
Rps25	0.001115	-0.23947	1
Hint2	0.002375	-0.33962	1
Rpl41	0.002616	-0.18317	1
Peg3	0.002851	-0.87483	1
Rnf220	0.003174	-0.2932	1
Rplp1	0.005094	-0.16596	1
Adamts1	0.006038	-0.5465	1
Pck2	0.00612	-0.28738	1
Eif2b1	0.0066	-0.24067	1

Ranked by Log2FC

Gene	p_val	avg_log2FC	p_val_adj
Cdkn1c	0.218081	0.978784202	1
Igf2	0.016468	0.870311067	1
Gpihbp1	0.003234	0.851538423	1
H19	0.009149	0.788964926	1
Mdk	0.160978	0.623917553	1
Apoe	0.234516	0.523936274	1
Ccnd1	0.069655	0.500791803	1
Bst2	0.043076	0.481641551	1
Celf1	0.001375	0.478418309	1
Siva1	0.022822	0.45129336	1
Hspa1a	0.265946	-0.957860989	1
Peg3	0.002851	-0.874834702	1
Lgals1	0.013413	-0.822280109	1
Rps2	7.18E-08	-0.645726595	0.002318
Hspb1	0.535309	-0.624419269	1
Hist1h3c	0.0729	-0.611836473	1
Casp3	0.011168	-0.551256991	1
Adamts1	0.006038	-0.546498283	1
Hspa1b	0.067881	-0.533781183	1
Gadd45g	0.007104	-0.516875375	1

Cluster 3 – Cycling capillaries (S)

Ranked by p value

Gene	p_val	avg_log2FC	p_val_adj
Arhgap42	3.94E-05	0.41966577	1
Fbxl3	0.000203	0.316617736	1
Nom1	0.000252	0.495964242	1
Dgkh	0.000411	0.502505782	1
A430106G13Rik	0.000415	0.126505997	1
Ptgr2	0.00046	0.219378439	1
Gpsm3	0.000521	0.279574407	1
A630072M18Rik	0.000745	0.212660187	1
Hipk2	0.000785	0.358344944	1
Mapk6	0.000789	0.352950167	1
Rps2	1.65E-07	-0.547120526	0.00534
Rplp2	0.000263	-0.25675798	1
Rpl36a	0.000571	-0.384246547	1
Rps26	0.001141	-0.258849648	1
Rpl31	0.001426	-0.276450886	1
Rpl36	0.001426	-0.353627745	1
Uqcr10	0.002022	-0.421236534	1
Slc25a4	0.002398	-0.318748639	1
Rpl38	0.002838	-0.353345982	1
Mcm6	0.003769	-0.486236239	1

Ranked by Log2FC

Gene	p_val	avg_log2FC	p_val_adj
Hist1h1b	0.886983	0.722487383	1
Igfbp3	0.085272	0.705670361	1
Prnd	0.005544	0.655083323	1
Sat1	0.017064	0.633576131	1
Filip1l	0.010444	0.603631256	1
Neurl3	0.076208	0.602881781	1
Hist1h1e	0.047593	0.572414219	1
Igf2	0.095273	0.556477231	1
Hspa1a	0.780404	0.527924532	1
Itm2a	0.064762	0.51367167	1
Lgals1	0.204271	-0.551189662	1
Rps2	1.65E-07	-0.547120526	0.00534
Edn1	0.314449	-0.54360743	1
Top2a	0.289974	-0.536447741	1
Ube2c	0.183903	-0.529443437	1
Junb	0.055708	-0.504691134	1
Mcm6	0.003769	-0.486236239	1
Snhg6	0.020293	-0.483629789	1
Bok	0.027197	-0.466545574	1
Ppih	0.037297	-0.462537922	1

Cluster 4 – Pre-arterial ECs

Ranked by p value

Gene	p_val	avg_log2FC	p_val_adj
Eif3m	0.000119	0.554738775	1
Tmem259	0.000188	0.326529927	1
Nhsl1	0.000245	0.414126956	1
Washc1	0.000431	0.327404959	1
Cnot2	0.000754	0.427832238	1
Sertad2	0.00077	0.3228216	1
D1Ertd622e	0.000778	0.527539427	1
Akr1a1	0.000785	0.362995178	1
Ppp1cb	0.000787	0.432257916	1
Gm4258	0.00096	0.490973903	1
Lamb1	0.000233	-0.64519466	1
Plat	0.000637	-0.644919333	1
Rhoc	0.000868	-0.517840713	1
Fam162a	0.001309	-0.464373887	1
Snhg6	0.002611	-0.476571392	1
Ppwd1	0.003108	-0.290945868	1
Nes	0.003946	-0.446447793	1
Sema3f	0.004284	-0.306099168	1
Dnajc24	0.004293	-0.384834972	1
Trib1	0.004855	-0.731839602	1

Ranked by Log2FC

Gene	p_val	avg_log2FC	p_val_adj
Tiparp	0.009744	0.86783055	1
Cavin2	0.015429	0.606246872	1
Btbd3	0.023565	0.561620114	1
Eif3m	0.000119	0.554738775	1
Cdc42ep3	0.073479	0.54450207	1
Clec14a	0.00328	0.5434638	1
Cldn5	0.016727	0.533343382	1
Hist1h2bc	0.190422	0.528619448	1
D1Ertd622e	0.000778	0.527539427	1
Abcg2	0.014897	0.520565794	1
Gm26917	0.12925	-3.095267775	1
Nts	0.014544	-1.884371705	1
AY036118	0.010038	-1.020831048	1
Fos	0.914494	-0.901906786	1
Egr1	0.020563	-0.858840253	1
Cxcl1	0.095591	-0.837843391	1
Tnc	0.005688	-0.785981855	1
Igfbp5	0.152472	-0.741471561	1
Trib1	0.004855	-0.731839602	1
Gadd45g	0.096854	-0.726784992	1

Cluster 5 – Tip cells

Ranked by p value

Gene	p_val	avg_log2FC	p_val_adj
Nckap1	0.00064	0.63026736	1
Btnl9	0.001587	0.741823723	1
Wdr36	0.001589	0.478788519	1
Fnip1	0.001967	0.550363037	1
Armcx4	0.001987	0.439326755	1
C1galt1	0.00236	0.407872862	1
Tro	0.002515	0.615841405	1
Sidt2	0.002865	0.363869815	1
Lama4	0.003327	0.648724764	1
Agps	0.004004	0.459190136	1
Rps2	4.74E-06	-0.443972202	0.153091
Rpl27	0.000417	-0.349904108	1
Rpl30	0.001058	-0.242215307	1
Gipc1	0.001315	-0.620148394	1
Ndufa4	0.001355	-0.529450154	1
Cd47	0.001356	-0.521404893	1
Sh3gl3	0.001436	-0.658069152	1
Rpl11	0.001496	-0.294767887	1
Ubac2	0.00154	-0.49390739	1
Rpp21	0.001649	-0.630830191	1

Ranked by Log2FC

Gene	p_val	avg_log2FC	p_val_adj
Igfbp5	0.01801	0.981049397	1
Kdm6b	0.011774	0.804367729	1
Btnl9	0.001587	0.741823723	1
Dab2	0.034824	0.73791772	1
Cox19	0.266407	0.699966939	1
Ift122	0.183222	0.692890979	1
Atad2b	0.016298	0.676233777	1
Fn1	0.013995	0.671981039	1
Adamts9	0.028446	0.671640355	1
Nsrp1	0.077285	0.667201968	1
Xist	0.09093	-0.747910089	1
Sde2	0.020343	-0.720800367	1
Sh3gl3	0.001436	-0.658069152	1
Zfp512	0.644441	-0.643466585	1
Rpp21	0.001649	-0.630830191	1
Gipc1	0.001315	-0.620148394	1
Ece1	0.003257	-0.611939817	1
Ppp1r11	0.002116	-0.606942239	1
Fabp4	0.045185	-0.599863737	1
Odc1	0.083285	-0.598406728	1

Cluster 6 – Venous ECs

Ranked by p value

Gene	p_val	avg_log2FC	p_val_adj
Wtip	0.00029	0.790020353	1
Agrn	0.000664	1.064052124	1
Nsd3	0.001013	0.874296003	1
Pak1ip1	0.001194	0.751742648	1
Diaph1	0.001209	0.640771408	1
Trappc6b	0.001357	0.672180863	1
Gna12	0.001411	0.620251361	1
Ptprg	0.001904	0.720076814	1
Parvb	0.002228	0.572123339	1
Ctdnep1	0.002331	0.669643628	1
Rps12	0.002144	-0.334220978	1
Rps2	0.002436	-0.658655664	1
Gpr180	0.0085	-0.754015522	1
Tjp1	0.009014	-0.780757121	1
Psmb3	0.010079	-0.774203726	1
Snrpe	0.010141	-0.593443475	1
Hnrnpa2b1	0.011329	-0.468482407	1
Hmgb2	0.012615	-0.838513448	1
Rplp2	0.012631	-0.337848501	1
Nudt4	0.014513	-0.720094626	1

Ranked by Log2FC

Gene	p_val	avg_log2FC	p_val_adj
Ccn2	0.058643	1.428921609	1
Peg3	0.112643	1.33874735	1
Igf2r	0.015921	1.115063762	1
Sox17	0.771611	1.083109485	1
Igf1	0.010474	1.072954376	1
Cdkn1c	0.141857	1.066965392	1
Agrn	0.000664	1.064052124	1
Cttnbp2nl	0.006032	1.044407039	1
Igfbp5	0.274563	1.041370175	1
Socs2	0.042166	0.977220331	1
Fos	0.762667	-1.892962345	1
Xist	0.071594	-0.910318708	1
Mrpl18	0.133962	-0.892184712	1
Hmgb2	0.012615	-0.838513448	1
Itch	0.030453	-0.824762922	1
Smc3	0.049188	-0.814839112	1
Tjp1	0.009014	-0.780757121	1
Npr3	0.166771	-0.779990691	1
Ggta1	0.282903	-0.77522344	1
Psmb3	0.010079	-0.774203726	1

Supplementary Figures

Supplementary Figure S1: Nkx2.5-CRE activity in the early embryo

A Representative *Nkx2.5-CRE;ROSA26-lacZ* embryos indicate the activity of *Nkx2.5-CRE* (detected by blue X-Gal stain) in the heart during embryonic development. Images show an E9.5 wholemount embryo, E11.5 wholemount embryo and dissected heart, and a sagittal section through an E11.5 embryo. Scale bars = 1mm, ht = heart; ra = right atrium; la = left atrium; rv = right ventricle; lv = left ventricle; oft = outflow tract. **B** Transverse sections through an *Nkx2.5-CRE;ROSA26-lacZ* E15.5 heart. Scale bars = 500µm for main images and 100 µm for magnified views i-iii. **C** Immunostaining for Endomucin (EMCN) and RFP in an E13.5 *Nkx2.5-CRE;ROSA26-tdTomato* heart, where RFP expression indicates CRE activity, alongside a littermate Cre-negative control *ROSA26-tdTomato* heart. Magnified images show RFP-negative ECs (white arrows) and two RFP-positive endocardial ECs (yellow arrows, bottom panel). Scale bars = 0.5mm (whole sections) and 10µm (magnified boxes). **D** Observed and expected numbers of pups genotyped at postnatal (P) days 10-20, from 56 litters. **E** Transverse sections through littermate P6 *Phd2*$^{fl/fl}$ and *Phd*$^{fl/fl}$*;Nkx2.5-CRE* hearts. Blue XGal staining shows arterial activity of Dll4-12:*LacZ* transgene. Scale bars = 1mm.

Alt text: Microscope images showing activity of the Nkx2.5-CRE line and postnatal heart sections, with subfigures labelled A-E. A and B are microscope images of blue X-Gal staining representing CRE activity in embryos and embryonic hearts. C is confocal images of red RFP expression representing CRE activity in the E13.5 heart, with magnified views highlighting RFP-negative endothelial cells with white arrows and RFP-positive endothelial cells with yellow arrows. D is microscope images of postnatal heart sections and E is a table showing expected and observed numbers of hypoxia mimic and control pups that survived to P10-20.

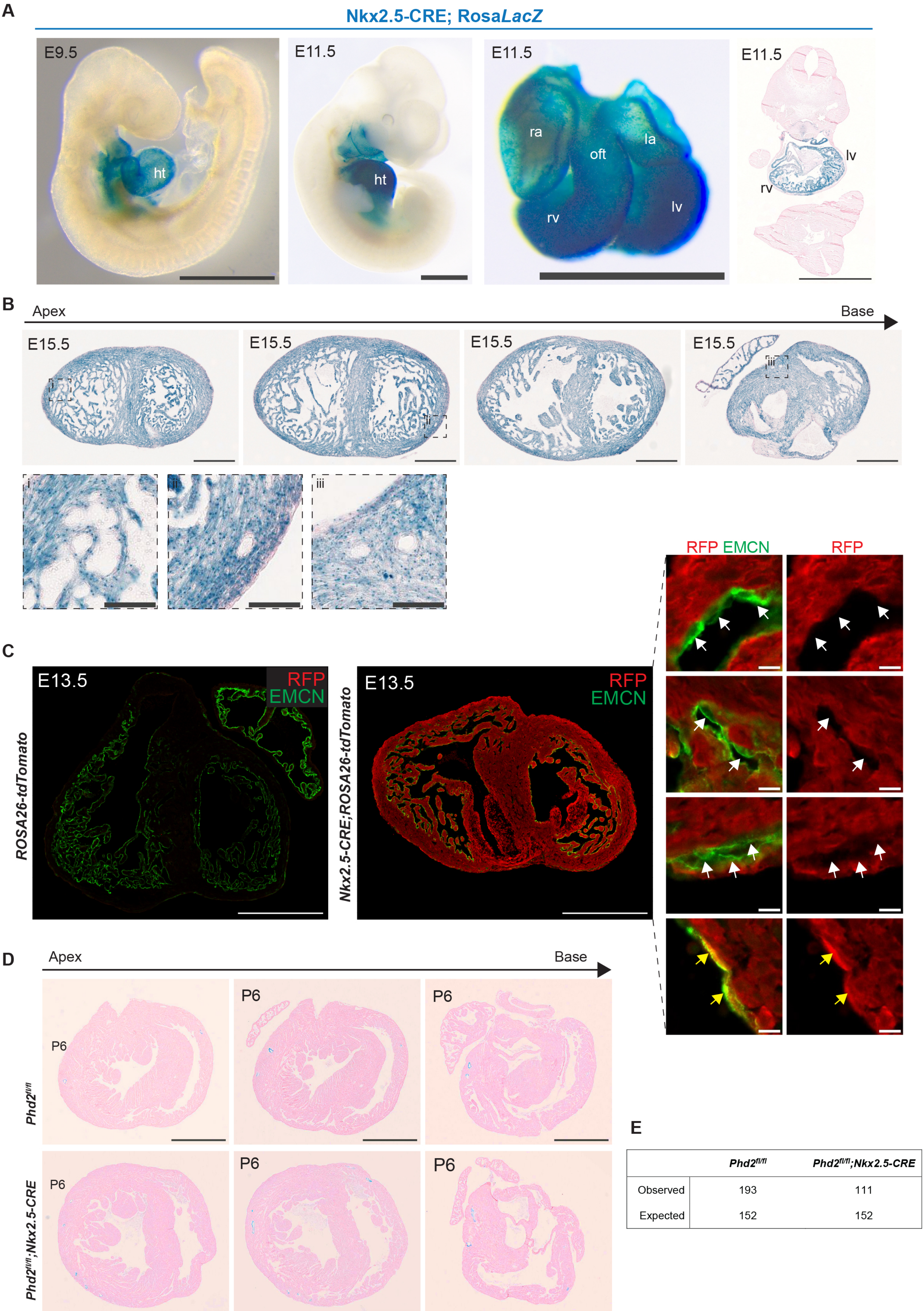

	Phd2[fl/fl]	*Phd2[fl/fl];Nkx2.5-CRE*
Observed	193	111
Expected	152	152

Supplementary Figure S2: Vascular development in hypoxic myocardium

A Mean estimated number of spots per *Vegfa*-positive cell in E13.5 *Phd2*$^{fl/fl}$ and *Phd2*$^{fl/fl}$*;Nkx2.5-CRE* heart sections, divided into ventricle and septum regions, calculated using QuPath software from *Vegfa* RNAScope data shown in Figure 1C-D. Unpaired t tests indicated no significant difference between *Phd2*$^{fl/fl}$ and *Phd2*$^{fl/fl}$*;Nkx2.5-CRE* results for either region (p=0.238 for ventricles, p=0.927 for septum). **B-D** Dorsal (**B-C**) and ventral (**D**) views of E12.5 (**B**) and E13.5 (**C-D**) *Phd2*$^{fl/fl}$ and *Phd2*$^{fl/fl}$*;Nkx2.5-Cre* hearts immunostained for the endothelial marker CD31, see also Figure 1H. Scale bars = 200μm for main images and 50μm for magnified views. **E** Quantification of the extension of the SV-derived vascular plexus as a percentage of the total dorsal area of E13.5 hearts. p=0.0535 (unpaired *t* test), error bars show SEM, N=2 hearts per genotype. **F** Quantification of the number of blood islands on the ventral aspect of E13.5 *Phd2*$^{fl/fl}$ and *Phd2*$^{fl/fl}$*;Nkx2.5-Cre* hearts. NS = not significant (unpaired *t* test), error bars show SEM, N=5 *Phd2*$^{fl/fl}$*;Nkx2.5-Cre* hearts and N= 7 *Phd2*$^{fl/fl}$ hearts. **G** Immunostaining for GLUT1 on transverse sections through E13.5 *Phd2*$^{fl/fl}$ and *Phd*$^{fl/fl}$*;Nkx2.5-CRE* ventricles. Scale bars = 50μm. Bar chart shows mean fluorescent signal across ventricles and septum from N=5 *Phd*$^{fl/fl}$*;Nkx2.5-CRE* and N=3 *Phd2*$^{fl/fl}$ hearts, 1-3 sections per heart. * = p<0.05, ** = p<0.01, unpaired t test.

Alt text: Confocal wholemount and section images further characterising the genetic mouse model being used to mimic myocardial hypoxia, along with graphs and a table to quantify imaging data, with subfigures labelled A-G.

A

	Phd2$^{fl/fl}$	*Phd2$^{fl/fl}$;Nkx2.5-CRE*
Ventricles	37.3	50.9
Septum	54.1	55.7

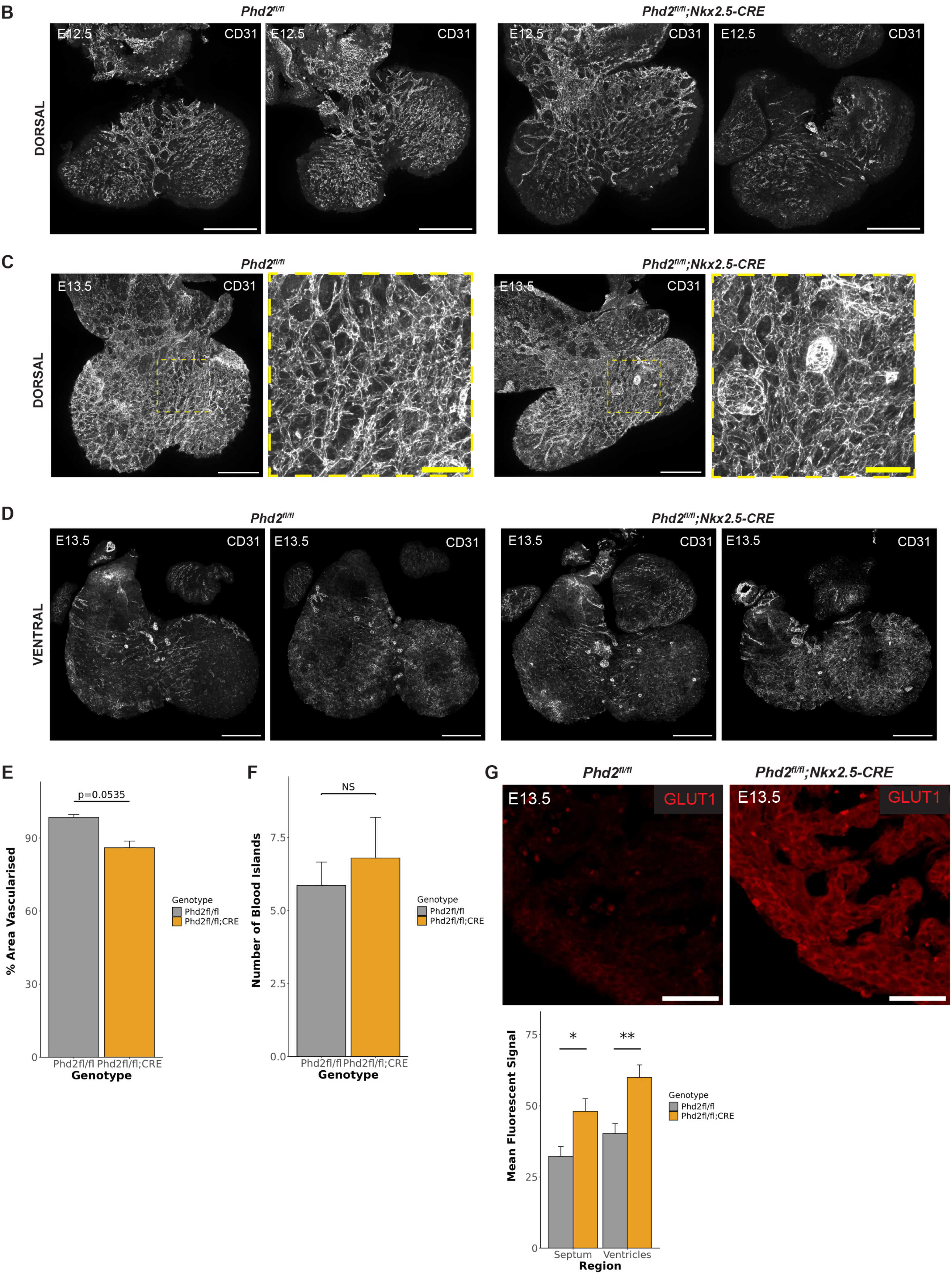

Supplementary Figure S3: Quality control for RNAseq dataset

A Sequencing statistics for the *Phd2*$^{fl/fl}$ and *Phd2*$^{fl/fl}$*;Nkx2.5-CRE* groups of cells. **B** Number of reads per cell, number of genes expressed per cell, and the percentage of genes mapped to the mitochondrial genome, shown per cluster for all the cells collected. **C** Number of reads per cell, number of genes expressed per cell, and the percentage of mitochondrial genes shown per cluster for the coronary EC-only dataset.

Alt text: Tables and violin plots showing quality control metrics for the single cell RNA-seq experiment, divided into subfigures A-C. A shows a table of sequencing metrics for cells from the control and hypoxia-mimic hearts. B and C show violin plots of reads per cell, genes per cell and percentage mitochondrial genes across clusters, for all cells and coronary endothelial cells, respectively.

A

	Phd2$^{fl/fl}$	*Phd2$^{fl/fl}$;Nkx2.5-Cre*
**Sequencing**		
Number of Reads	349950236	296927485
Valid Barcodes	96.9%	96.7%
Sequencing Saturation	53.3%	50.3%
Q30 Bases in Barcode	96.9%	96.9%
Q30 Bases in RNA Read	95.7%	95.7%
Q30 Bases in UMI	97.2%	97.2%
**Mapping**		
Reads Mapped to Genome	94.7%	94.2%
Reads Mapped Confidently to Genome	91.6%	91.1%
Reads Mapped Confidently to Intergenic Regions	6.1%	6.0%
Reads Mapped Confidently to Intronic Regions	31.3%	31.4%
Reads Mapped Confidently to Exonic Regions	54.2%	53.6%
Reads Mapped Confidently to Transcriptome	49.5%	49.0%
Reads Mapped Antisense to Gene	3.6%	3.5%
**Cells**		
Estimated Number of Cells	5403	3200
Fraction Reads in Cells	91.7%	90.7%
Mean Reads per Cell	64770	92790
Median Genes per Cell	3538	4620
Total Genes Detected	22680	22282
Median UMI Counts per Cell	10842	17678

B

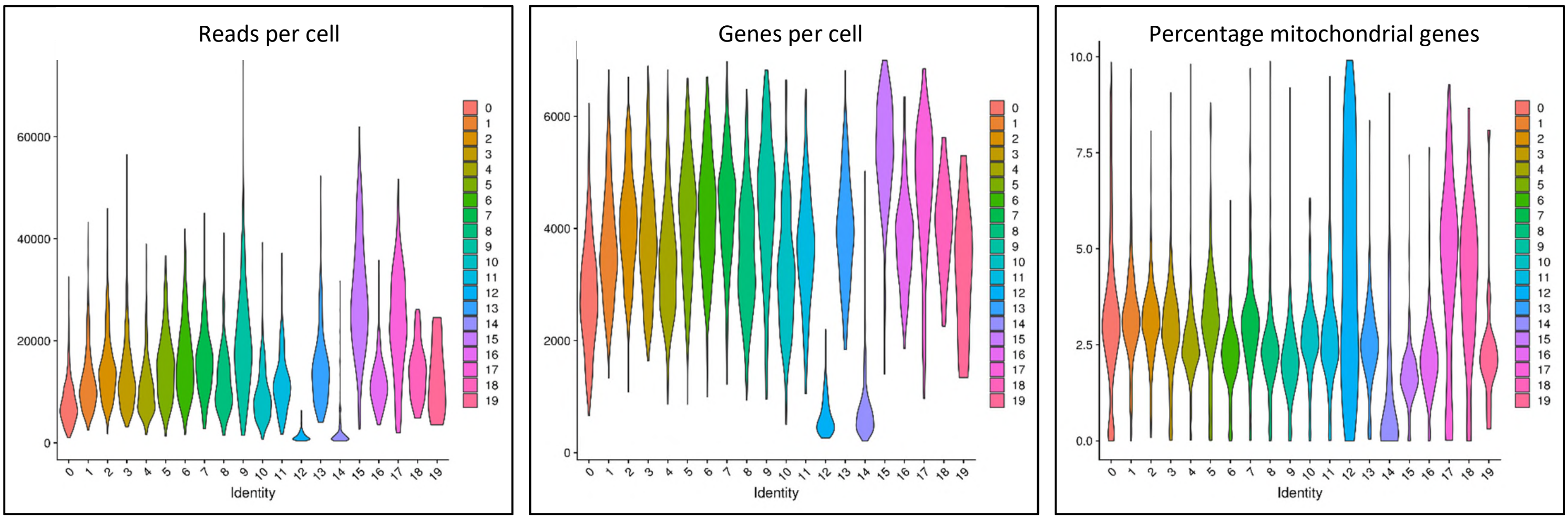

C

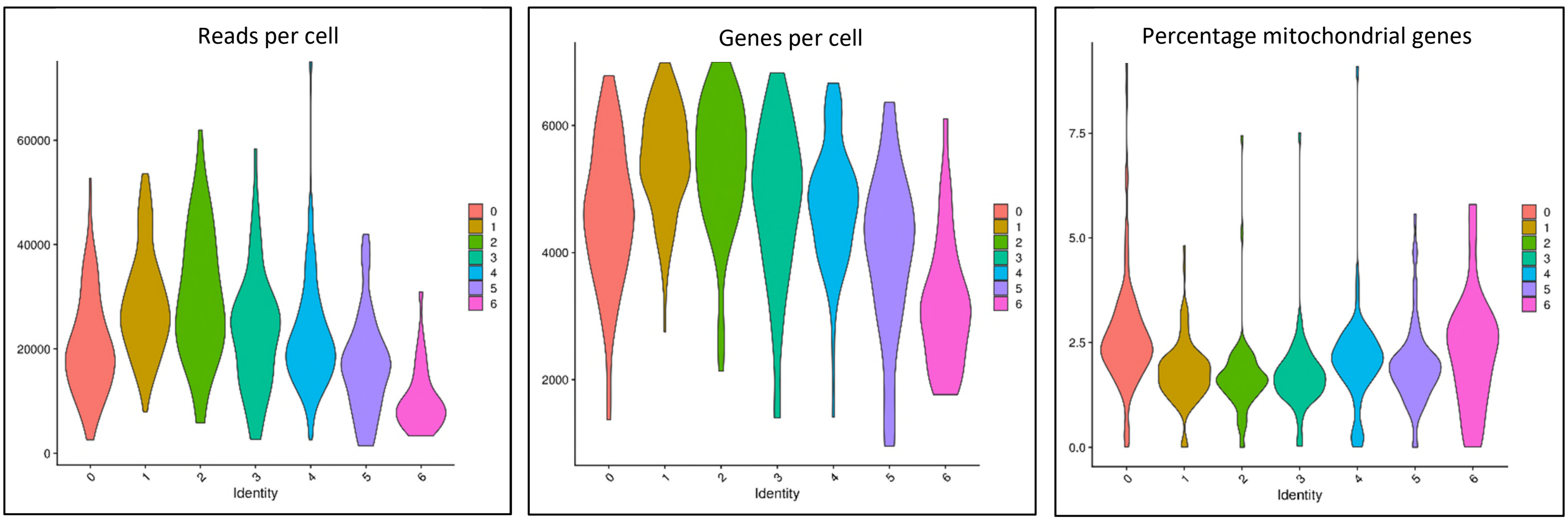

Supplementary Figure S4: SOX17 expression in the developing coronary vasculature

Ventral and dorsal views of E12.5 (**A**) and E13.5 (**B**) hearts with immunostaining for SOX17 protein and the endothelial marker Endomucin (EMCN). Magnified views show nuclear SOX17 throughout the developing vasculature and within round blood islands on the ventral aspect. Scale bars = 200µm for main images and 50µm for magnified views.

Alt text: Wholemount confocal images of embryonic hearts, showing ventral and dorsal views, with subfigures labelled A-B. Hearts are immunostained for endothelial EMCN, shown in white, and the transcription factor SOX17, shown in magenta. Next to each wholemount image is a magnified region showing blood islands or the vascular plexus. Subfigure A shows E12.5 hearts and B shows E13.5 hearts.

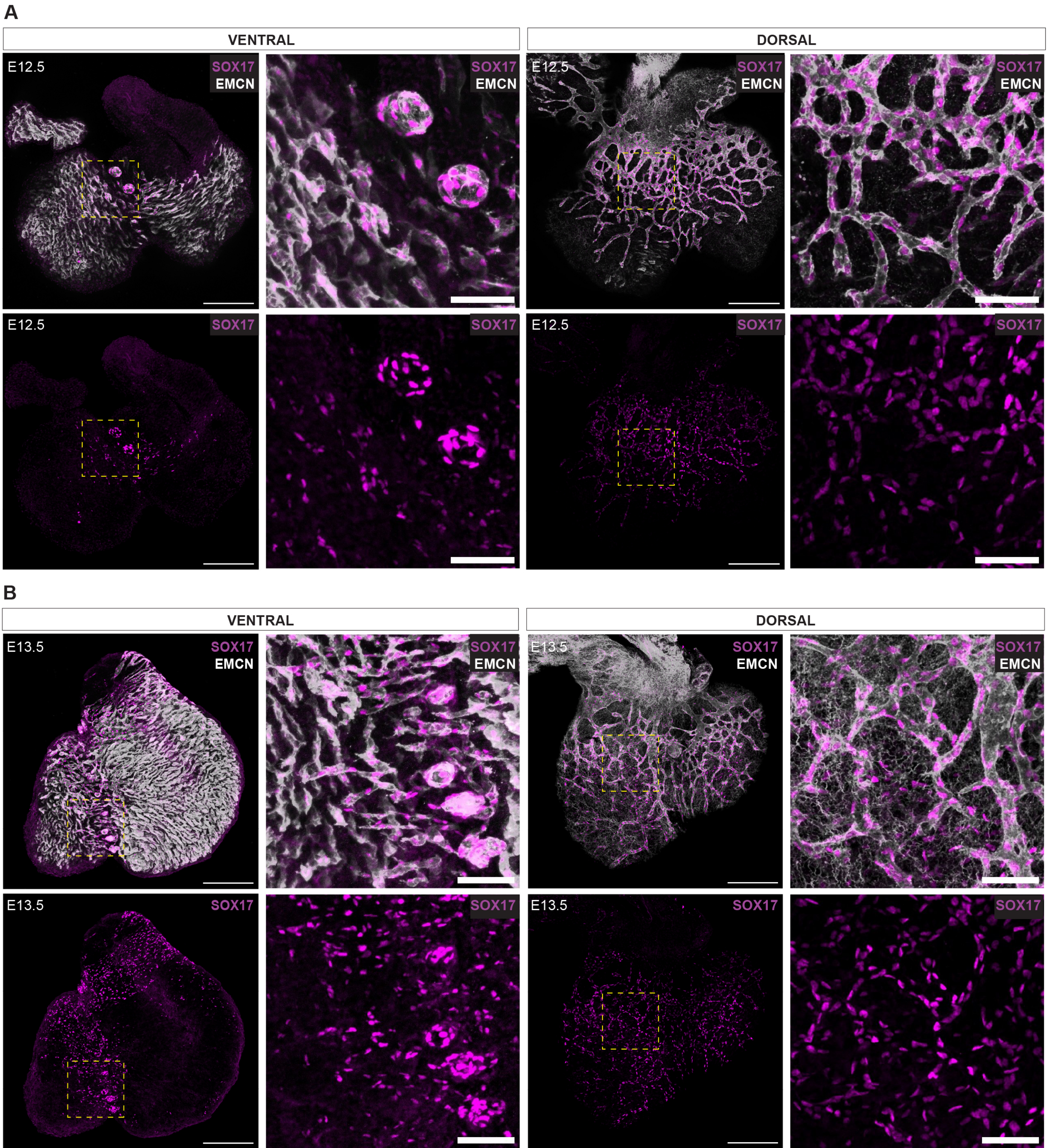
A
VENTRAL
DORSAL
E12.5
SOX17
EMCN
B
E13.5

Supplementary Figure S5: E11.75 and E12.5 HLX-3:*LacZ* hearts

A Additional *Phd2*$^{fl/fl}$ and *Phd2*$^{fl/fl}$*;Nkx2.5-CRE* hearts collected at E11.75 expressing the HLX-3:*LacZ* transgene, collected across two litters. The hearts shown in Figure 4C were also from Litter 1. **B** Two additional litters of *Phd2*$^{fl/fl}$ and *Phd2*$^{fl/fl}$*;Nkx2.5-CRE* E12.5 hearts. The hearts in Litter 2 were stained for longer than other hearts, so the blue XGal stain is stronger.

All scale bars = 1mm.

Alt text: Microscope images of wholemount embryonic control and hypoxia-mimic hearts, showing ventral and dorsal views, with activity of the angiogenic HLX-3:*LacZ* transgene shown by blue X-Gal staining. The figure is divided into subfigures A and B, showing images of E11.75 and E12.5 hearts respectively. For each developmental stage, hearts are separated into individual litters.

Hlx-3:*LacZ* Activity: Angiogenic ECs

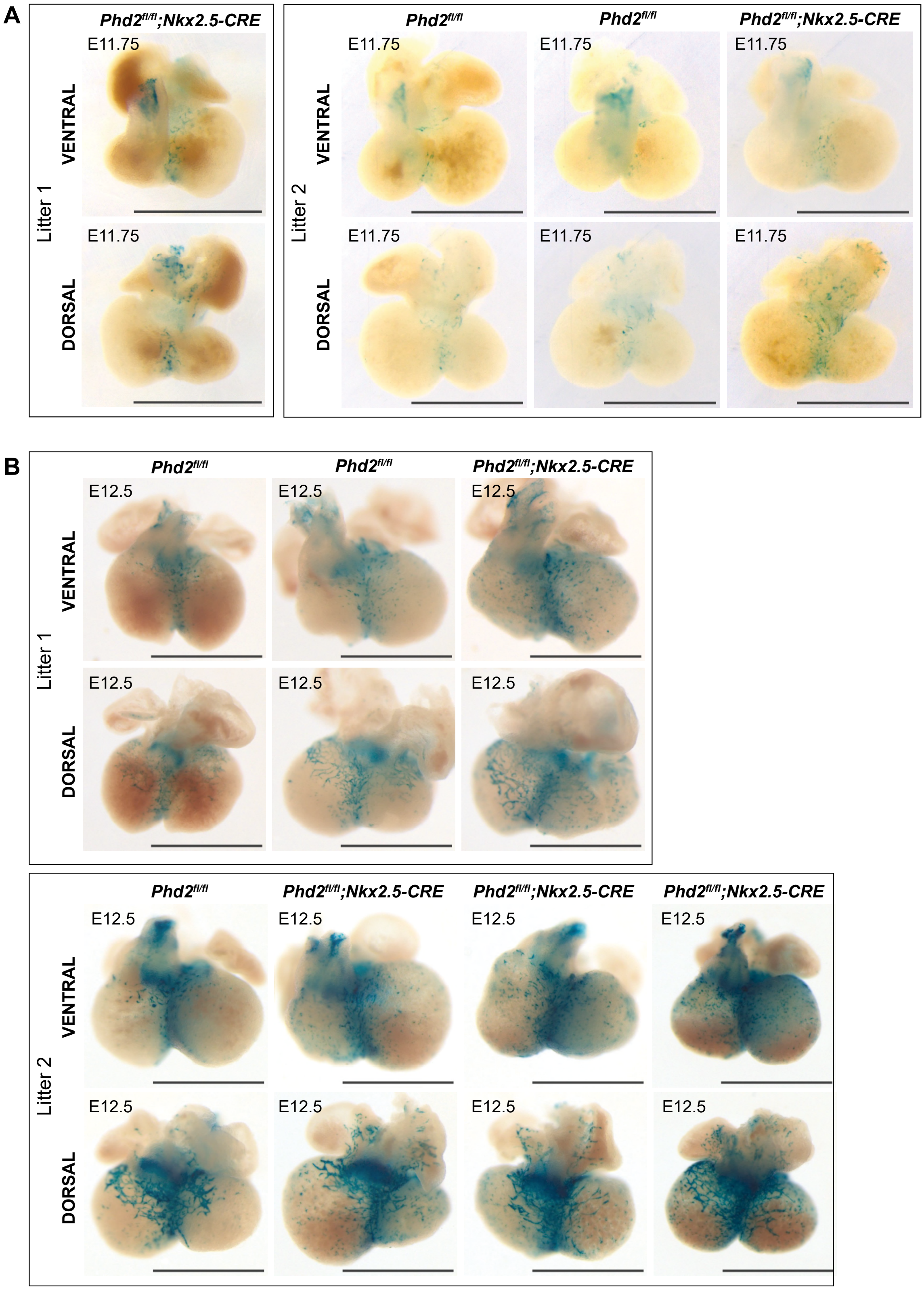

Supplementary Figure S6: E13.5 HLX-3:*LacZ* hearts

3 litters of *Phd2*$^{fl/fl}$ and *Phd2*$^{fl/fl}$*;Nkx2.5-CRE* hearts collected at E13.5 expressing the HLX-3:*LacZ* transgene, in addition to the littermate hearts shown in Figure 4F. Scale bars = 1mm.

Alt text: Microscope images of wholemount E13.5 control and hypoxia-mimic hearts, with activity of the angiogenic HLX-3:*LacZ* transgene shown by blue X-Gal staining on ventral and dorsal aspects. Hearts are separated into three individual litters.

Hlx-3:*LacZ* Activity: Angiogenic ECs

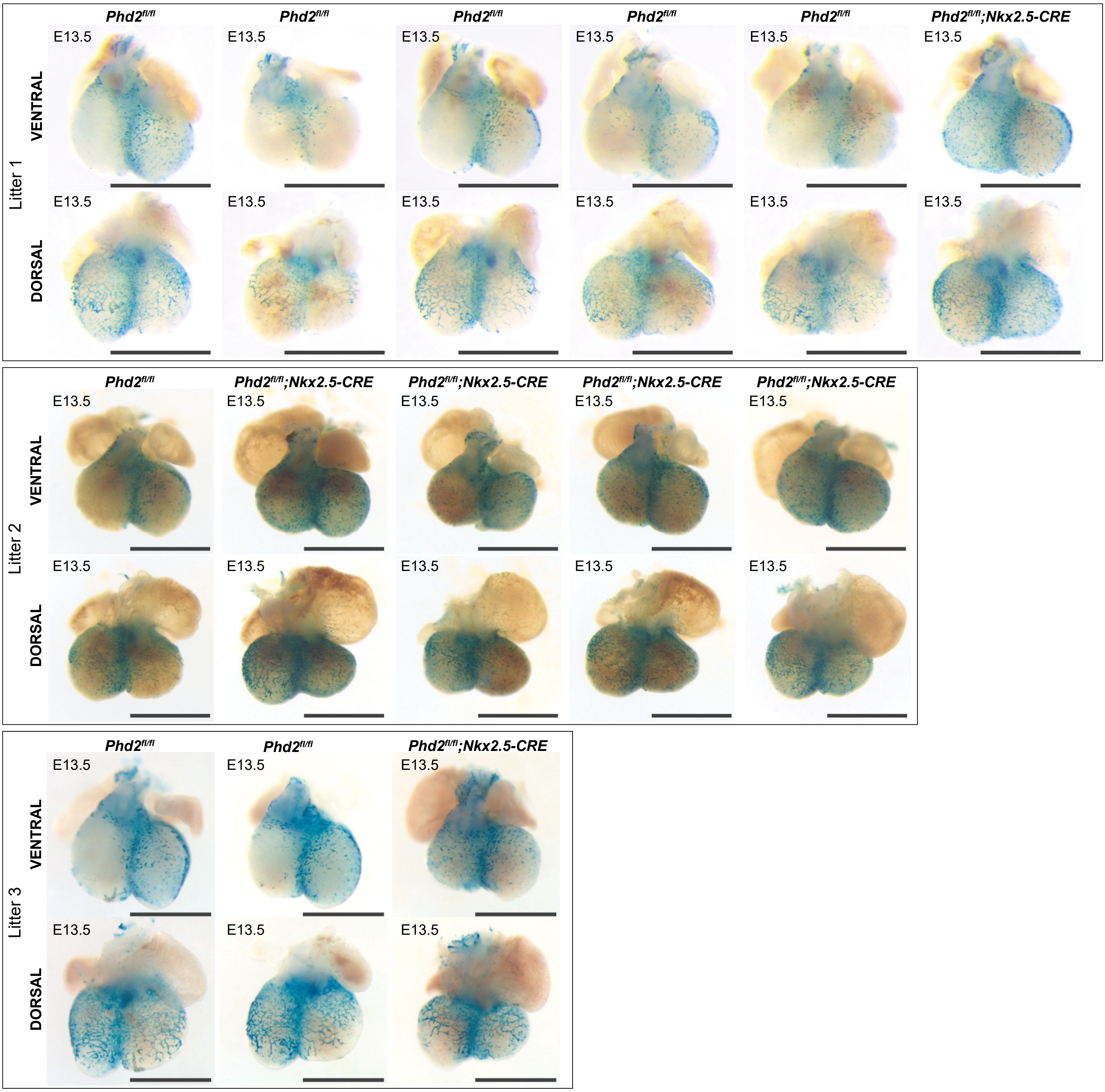

Supplementary Figure S7: E15.5 HLX-3:*LacZ* hearts

A All additional hearts collected at E15.5 across 5 litters, hearts shown in main Figure 4I were from Litter 1. Litter 5 contained 3 heterozygous *Phd2*$^{fl/+}$*;Nkx2.5-CRE* hearts, whose expression is consistent with the *Phd2*$^{fl/fl}$ hearts and therefore show that Cre activity is not affecting HLX-3:*LacZ* activity. Scale bars = 1mm. **B** Transverse sections through a *Phd2*$^{fl/fl}$ and *Phd2*$^{fl/fl}$*;Nkx2.5-CRE* heart, with magnified image of the interventricular septum (IVS). Scale bars = 0.2mm.

Alt text: Wholemount and section microscope images comparing E15.5 control and hypoxia-mimic hearts, divided into subfigures A-B. Activity of the angiogenic HLX-3:*LacZ* transgene is shown by blue X-Gal staining. A shows ventral and dorsal views of wholemount hearts separated into five individual litters. B shows transverse sections through a control and hypoxia-mimic heart, with magnified images of the interventricular septum region.

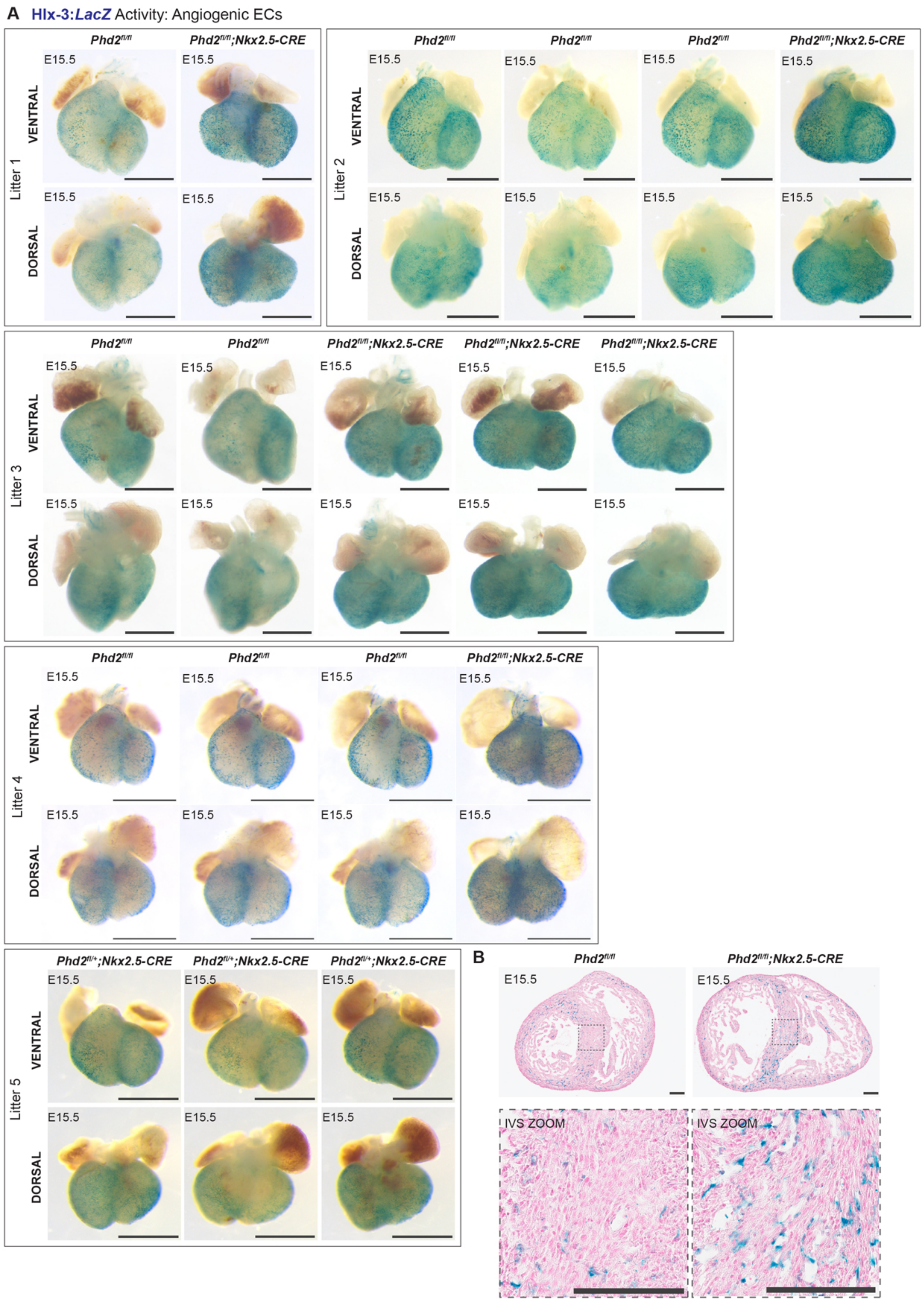

A Hlx-3:LacZ Activity: Angiogenic ECs
Litter 1
Phd2fl/fl
Phd2fl/fl;Nkx2.5-CRE
VENTRAL
DORSAL
E15.5
Litter 2
Phd2fl/fl
Phd2fl/fl
Phd2fl/fl
Phd2fl/fl;Nkx2.5-CRE
Litter 3
Phd2fl/fl
Phd2fl/fl
Phd2fl/fl;Nkx2.5-CRE
Phd2fl/fl;Nkx2.5-CRE
Phd2fl/fl;Nkx2.5-CRE
Litter 4
Phd2fl/fl
Phd2fl/fl
Phd2fl/fl
Phd2fl/fl;Nkx2.5-CRE
Litter 5
Phd2fl/+;Nkx2.5-CRE
Phd2fl/+;Nkx2.5-CRE
Phd2fl/+;Nkx2.5-CRE
B
Phd2fl/fl
Phd2fl/fl;Nkx2.5-CRE
E15.5
IVS ZOOM

Supplementary Figure S8: E12.5 and E13.5 NOTCH1+16:*LacZ* hearts

A Additional *Phd2*$^{fl/fl}$ and *Phd2*$^{fl/fl}$*;Nkx2.5-CRE* hearts collected at E12.5 expressing the NOTCH1+16:*LacZ* transgene, collected across four litters. Litter 1 also contained the two hearts shown in Figure 5B. Scale bars = 1mm. **B** Additional hearts collected at E13.5 expressing the NOTCH1+16:*LacZ* transgene. The two hearts shown in Figure 5C were from Litter 1. Scale bars = 1mm. **C** Transverse sections through two E13.5 control *Phd2*$^{fl/fl}$ and two *Phd2*$^{fl/fl}$*;Nkx2.5-CRE* hearts showing NOTCH1+16:*LacZ* transgene activity. The sections in top two panels come from the hearts shown in Figure 5C, the sections in bottom two panels come from Supplemental Figure S8B Litter 1. Scale bars = 0.2mm.

Alt text. Wholemount and section microscope images comparing NOTCH1+16:*LacZ* transgene activity in the sinus-venosus derived coronary vessels between control and hypoxia-mimic hearts, divided into subfigures A-C. Activity of the transgene is shown by blue X-Gal staining. A shows ventral and dorsal views of wholemount 12.5 hearts separated into four individual litters. B shows wholemount E13.5 hearts separated into two litters. C shows blue transgene activity on transverse sections through a control and hypoxia-mimic E13.5 heart.

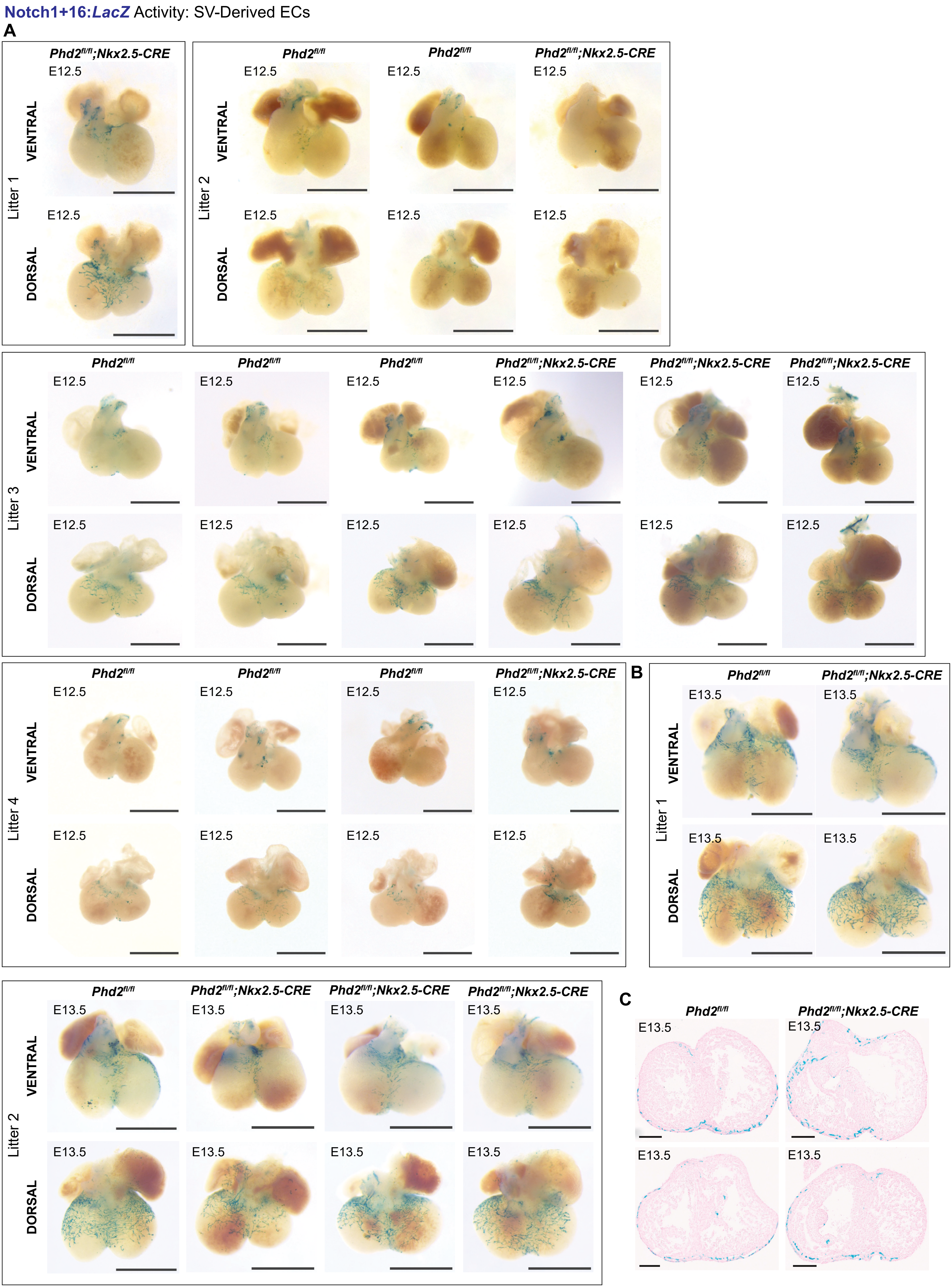

Notch1+16:LacZ Activity: SV-Derived ECs
A
B
C
Litter 1
Litter 2
Litter 3
Litter 4
VENTRAL
DORSAL
Phd2fl/fl
Phd2fl/fl;Nkx2.5-CRE
E12.5
E13.5

Supplementary Figure S9: E15.5 NOTCH1+16:*LacZ* hearts

Additional *Phd2*$^{fl/fl}$ and *Phd2*$^{fl/fl}$*;Nkx2.5-CRE* hearts expressing the NOTCH1+16:*LacZ* transgene, collected at E15.5. Litter 1 also contained the hearts shown in Figure 5F. Scale bars = 1mm.

Alt text: Microscope images of ventral and dorsal views of wholemount E15.5 hearts, comparing blue NOTCH1:*LacZ* transgene activity in control and hypoxia-mimic littermate hearts. Hearts are divided into four litters.

Notch1+16:*LacZ* Activity: SV-Derived ECs

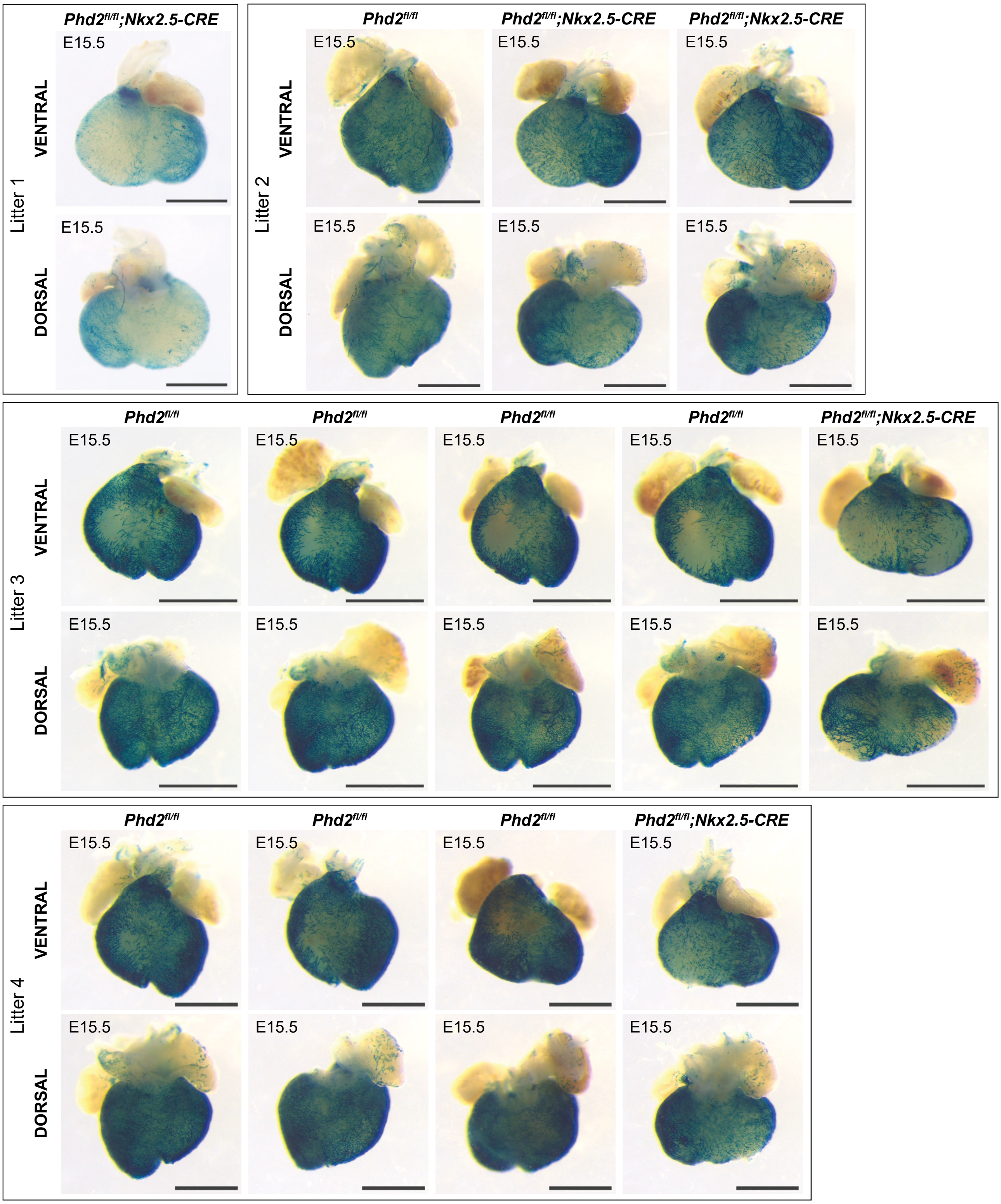

Supplementary Figure S10: E13.5 Dll4-12:*LacZ* hearts

Additional *Phd2*$^{fl/fl}$ and *Phd2*$^{fl/fl}$*;Nkx2.5-CRE* hearts expressing the Dll4-12:*LacZ* transgene, collected at E13.5. The hearts shown in main Figure 6B were also from Litter 1. Scale bars = 1mm.

Alt text: Microscope images of ventral and dorsal views of wholemount E13.5 hearts, with blue X-Gal staining showing activity of the arterial-specific Dll4-12:LacZ transgene in control and hypoxia-mimic hearts. Black boxes separate the hearts into three litters.

Dll4-12:*LacZ* Activity: Arterial ECs

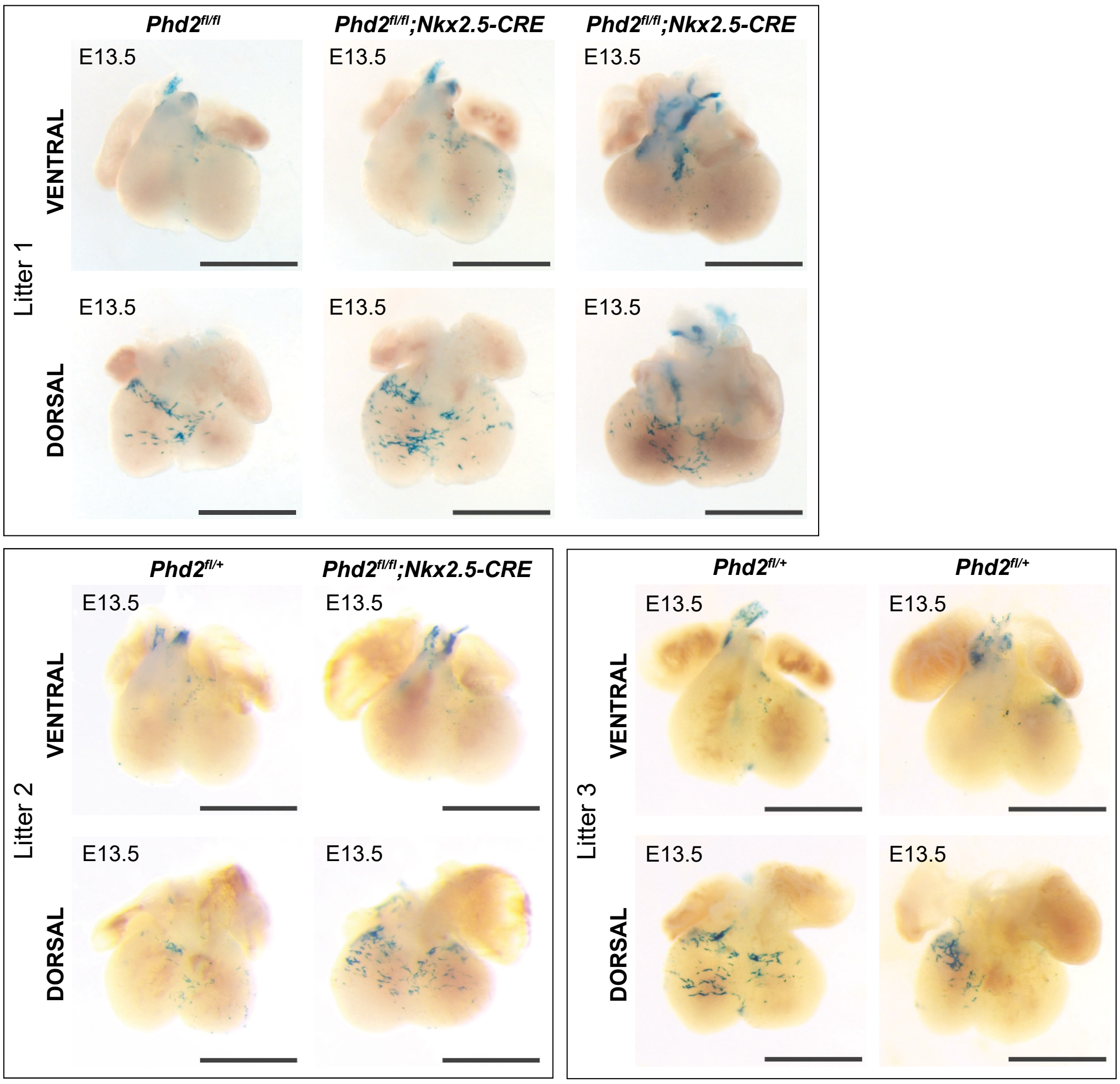

Supplementary Figure S11: E15.5 Dll4-12:*LacZ* hearts

Additional 5 litters of E15.5 hearts to those shown in Figure 6D, showing expression of the Dll4-12:*LacZ* transgene on different genetic backgrounds. The heterozygous *Phd2*$^{fl/+}$*;Nkx2.5-CRE* hearts show more similar activity to their CRE-negative littermate hearts, and therefore indicate there is no effect of CRE expression on the enhancer activity. Black arrows indicate left anterior descending artery. Scale bars = 1mm.

Alt text: Microscope images of ventral and dorsal views of wholemount E15.5 control and hypoxia-mimic hearts, with blue X-Gal staining showing activity of the arterial-specific Dll4-12:LacZ transgene. Hearts are divided into five individual litters, and clearly differentiated left anterior descending coronary arteries are highlighted with black arrows.

Dll4-12:*LacZ* Activity: Arterial ECs

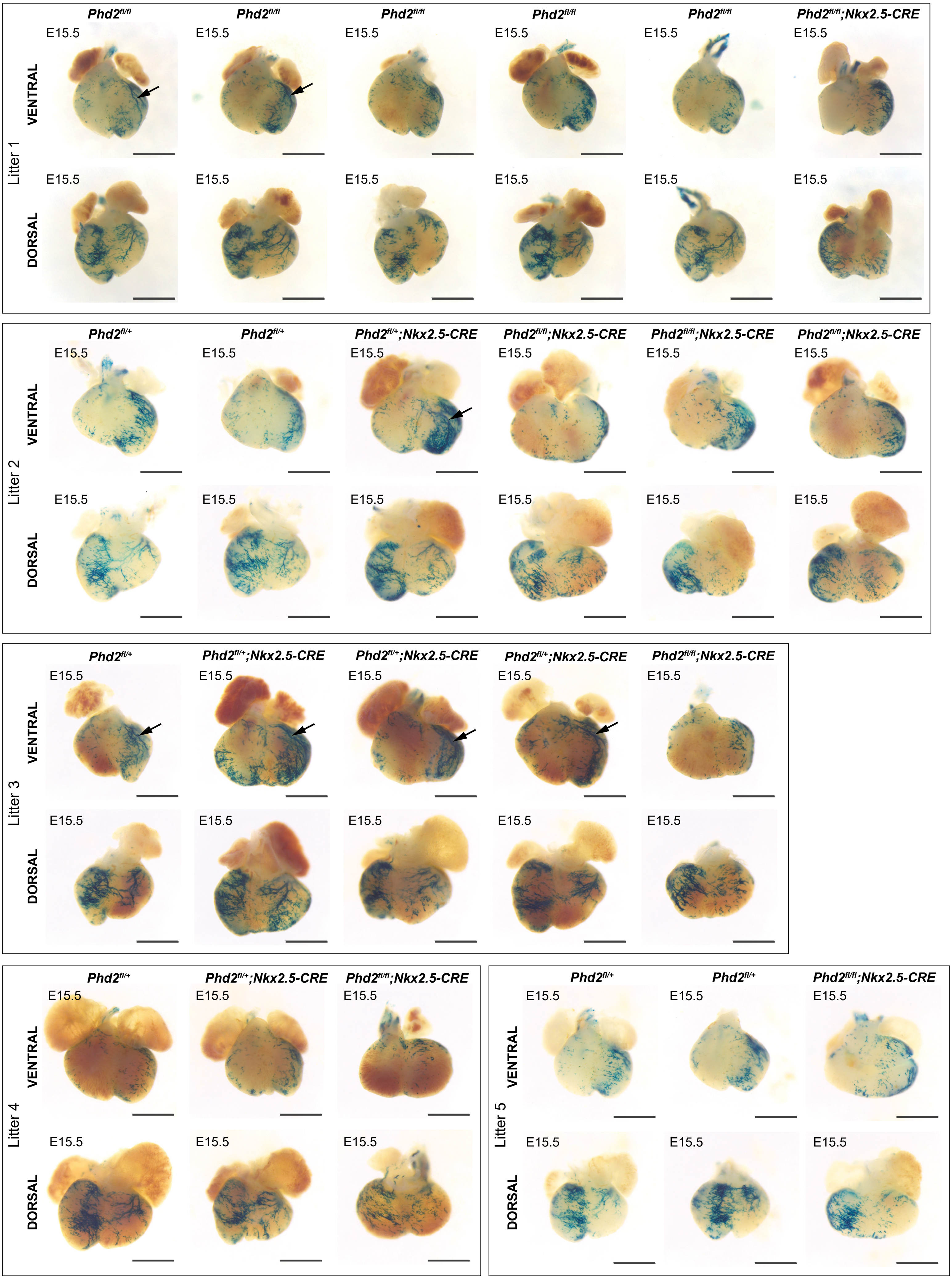

Supplementary Figure S12: E15.5-18.5 Dll4-12:*LacZ* hearts

A Images of Dll4-12:*LacZ* activity in *Phd2*$^{fl/fl}$ and *Phd2*$^{fl/fl}$*;Nkx2.5-CRE* hearts viewed from the top, in addition to those shown in Figure 6E. **B** Transverse sections through two E15.5 *Phd2*$^{fl/fl}$ and two *Phd2*$^{fl/fl}$*;Nkx2.5-CRE* hearts, showing Dll4-12:*LacZ* transgene. The sections in the top two panels are from the hearts shown in Figure 6D, the sections in the two bottom panels are from hearts shown in Supplementary Figure S11, Litter 1. Scale bars = 0.2mm. **C** 3 additional litters of E18.5 hearts to those shown in Figure 6F, showing expression of the Dll4-12:*LacZ* transgene on different genetic backgrounds. The heterozygous *Phd2*$^{fl/+}$*;Nkx2.5-CRE* hearts are controls for CRE activity. Black arrows indicate left anterior descending artery. **D** Additional biological replicates of wholemount E17.5 hearts immunostained for smooth muscle MYH11 and venous EMCN to those shown in Figure 6H. Scale bars = 1mm for main images and 0.2mm for magnified views.

Alt text: Microscope images of wholemount hearts and transverse heart sections, with subfigures labelled A-D. A contains top views of E15.5 control and hypoxia-mimic hearts with blue X-gal staining showing arterial Dll4-12:*LacZ* transgene activity. B contains transverse sections through two each of control and hypoxia-mimic hearts with blue Dll4-12:*LacZ* transgene activity. C shows microscope images of ventral and dorsal views of E18.5 control and hypoxia-mimic hearts with blue Dll4-12:*LacZ* transgene activity, with black arrows highlighting the left anterior descending coronary artery on ventral views. D shows confocal wholemount images of E17.5 control and hypoxia-mimic hearts immunostained for venous EMCN, shown in green, and arterial MYH11, shown in magenta.

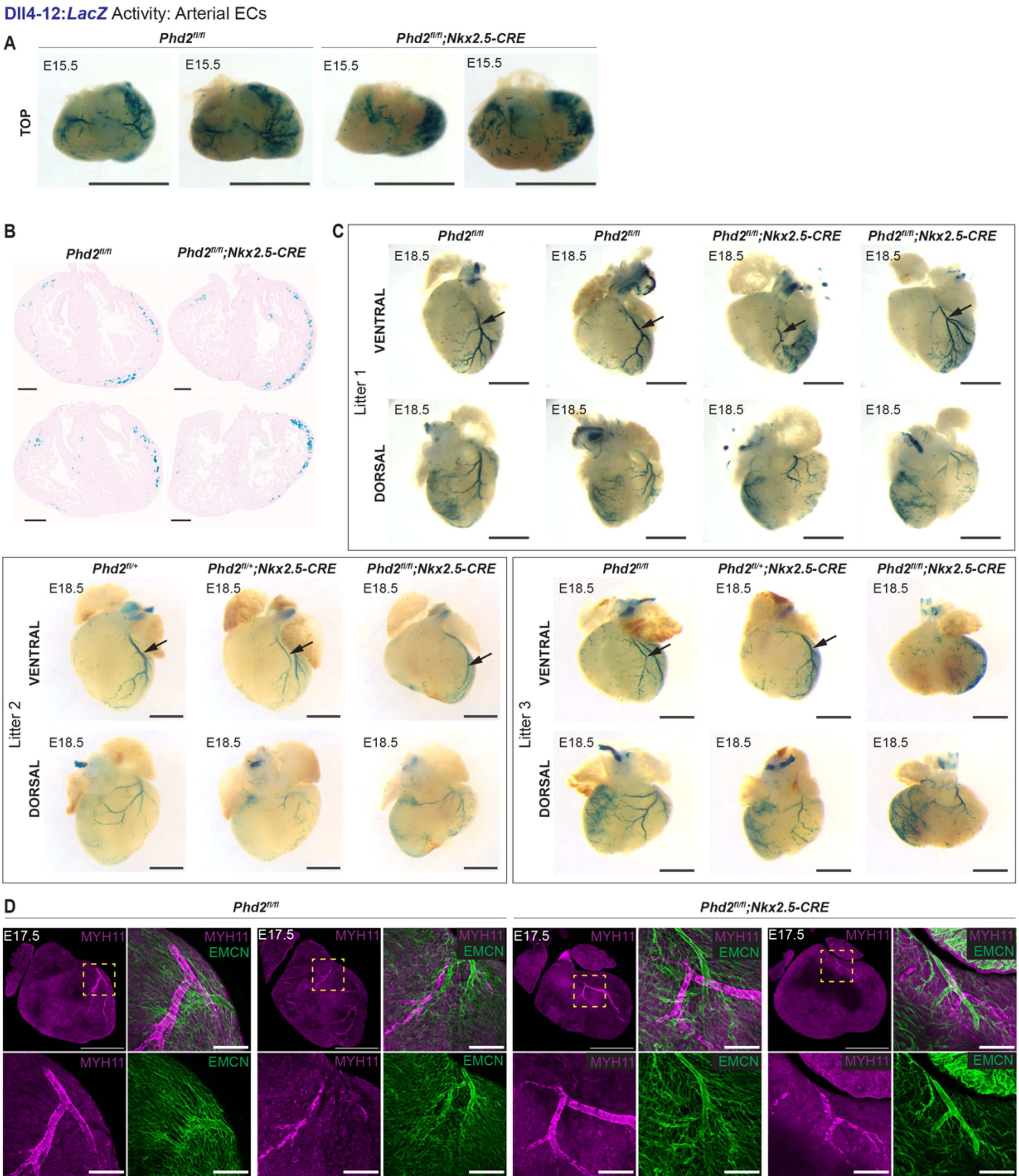

Dll4-12:LacZ Activity: Arterial ECs
A
Phd2fl/fl
Phd2fl/fl;Nkx2.5-CRE
TOP
E15.5
B
Phd2fl/fl
Phd2fl/fl;Nkx2.5-CRE
C
Phd2fl/fl
Phd2fl/fl;Nkx2.5-CRE
Litter 1
VENTRAL
DORSAL
E18.5
Litter 2
Phd2fl/+
Phd2fl/+;Nkx2.5-CRE
Phd2fl/fl;Nkx2.5-CRE
Litter 3
Phd2fl/fl
Phd2fl/+;Nkx2.5-CRE
Phd2fl/fl;Nkx2.5-CRE
D
Phd2fl/fl
Phd2fl/fl;Nkx2.5-CRE
E17.5
MYH11
EMCN

Supplementary Figure S13: P6-P8 Dll4-12:*LacZ* hearts

Postnatal activity of Dll4-12:*LacZ* in P6-P8 hearts shows similar activity in hypoxic myocardium and controls. Black arrows indicate left anterior descending artery.

Alt text: Microscope images of ventral and dorsal views of wholemount postnatal P6-P8 hearts, comparing blue Dll4-12:*LacZ* transgene activity in control and hypoxia-mimic littermate hearts. Hearts are divided into six litters, and black arrows indicate left anterior descending coronary arteries.

Dll4-12:*LacZ* Activity: Arterial ECs

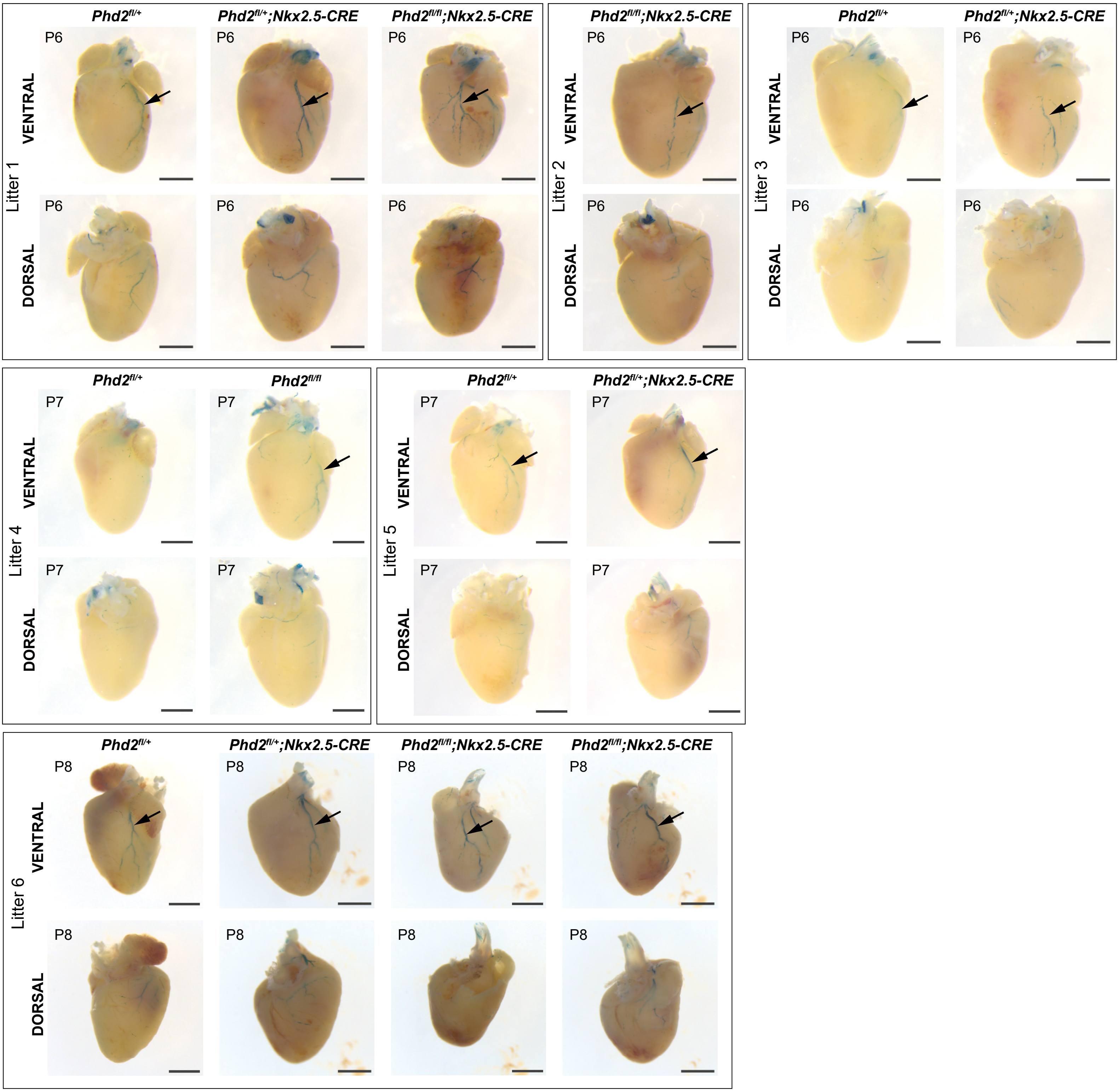

Supplementary Figure S14: E13.5 and E15.5 EphB4-2:*LacZ* hearts

A E13.5 hearts showing expression of the EphB4-2:*LacZ* transgene on *Phd2*$^{fl/fl}$ and *Phd2*$^{fl/fl}$*;Nkx2.5-CRE* backgrounds, in addition to those shown in Figure 7B. The hearts in Figure 7B were also from Litter 1. Scale bars = 1mm. **B** Activity of EphB4-2:*LacZ* in E15.5 hearts, in addition to those in Figure 7D. Scale bars = 1mm. **C** Transverse sections through the *Phd2*$^{fl/fl}$ and *Phd2*$^{fl/fl}$*;Nkx2.5-CRE* hearts shown in Figure 7D showing activity of the EphB4-2:*LacZ* transgene. Scale bars = 0.2mm.

Alt text: Microscope images of E13.5 and E15.5 control and hypoxia-mimic hearts, with blue X-Gal staining showing activity of the venous EphB4-2:*LacZ* transgene, divided into subfigures A-C. A and B show ventral and dorsal views of wholemount E13.5 and E15.5 hearts respectively, with hearts divided by black outline boxes into individual litters. C shows transverse sections through a control and hypoxia-mimic E15.5 heart.

EphB4-2:*LacZ* Activity: Venous ECs

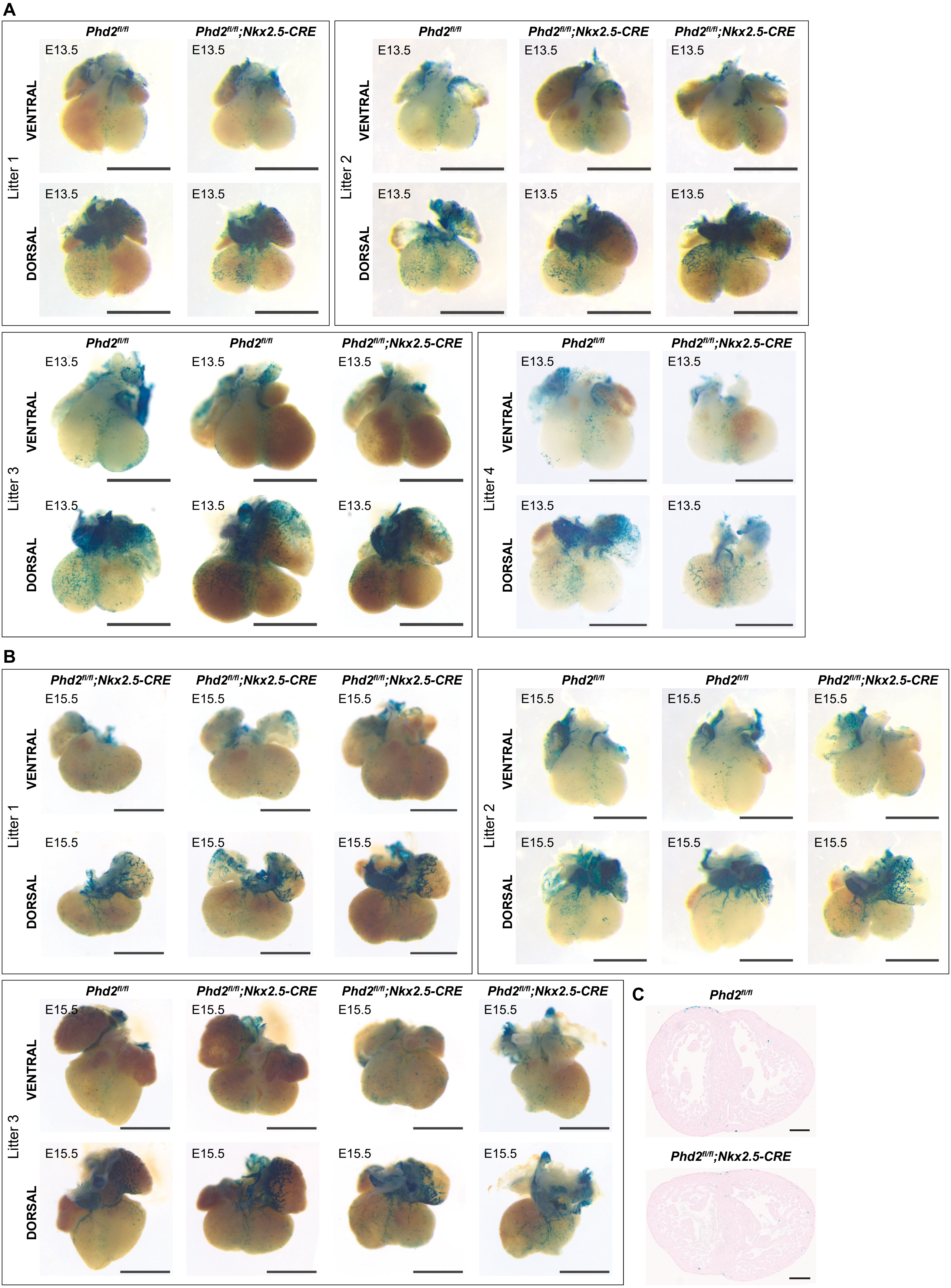

Supplementary Figure S15: E18.5 EphB4-2:*LacZ* hearts

A Additional *Phd2*$^{fl/fl}$ and *Phd2*$^{fl/fl}$*;Nkx2.5-CRE* hearts expressing the EphB4-2:*LacZ* transgene, collected at E18.5. Litter 1 also contained the hearts shown in Figure 7E. Scale bars = 1mm. **B** Additional biological replicates for the E15.5 wholemount hearts shown in Figure 7F, immunostained for venous NR2F2 and EMCN. Scale bars = 1mm for main images and 0.2mm for magnified views.

Alt text: Microscope images of wholemount embryonic hearts, divided into subfigures A-B. A shows ventral and dorsal views of E18.5 hearts with blue X-Gal staining of venous EphB4-2:*LacZ* transgene activity in control and hypoxia-mimic littermate hearts. B contains confocal images of two control and two hypoxia-mimic E15.5 hearts immunostained for the venous markers EMCN, shown in white, and NR2F2, shown in magenta. Magnified views of cardiac veins are shown for each heart.

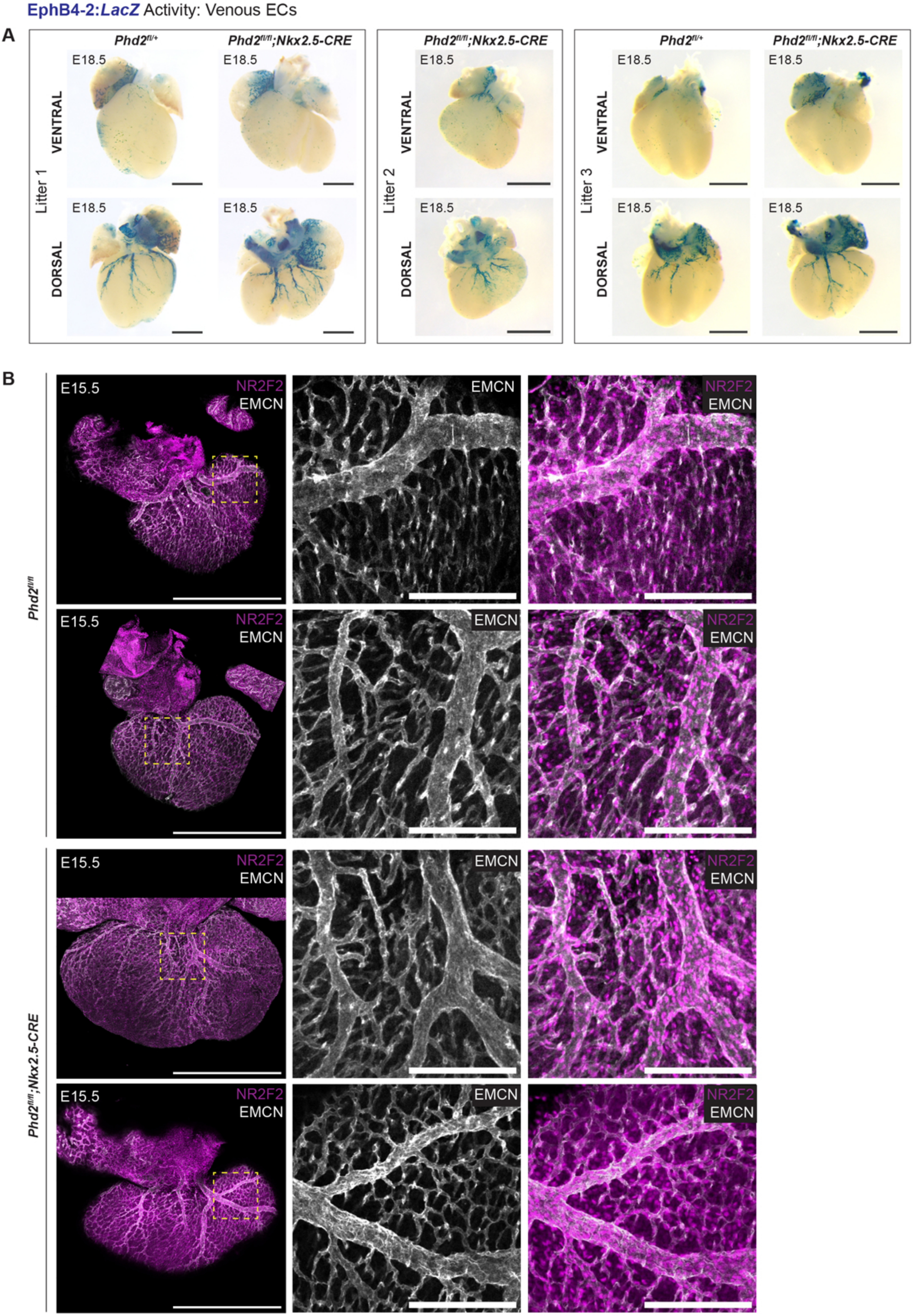

EphB4-2:LacZ Activity: Venous ECs
A
Phd2fl/+
Phd2fl/fl;Nkx2.5-CRE
Phd2fl/fl;Nkx2.5-CRE
Phd2fl/+
Phd2fl/fl;Nkx2.5-CRE
Litter 1
Litter 2
Litter 3
VENTRAL
DORSAL
E18.5
B
Phd2fl/fl
Phd2fl/fl;Nkx2.5-CRE
E15.5
NR2F2
EMCN
